# In-Depth Profiling of *O*-Glycan Isomers in Human Cells Using C18 Nanoliquid Chromatography–Mass Spectrometry and Glycogenomics

**DOI:** 10.1021/acs.analchem.1c05068

**Published:** 2022-03-04

**Authors:** Noortje de Haan, Yoshiki Narimatsu, Mikkel Koed Møller Aasted, Ida S. B. Larsen, Irina N. Marinova, Sally Dabelsteen, Sergey Y. Vakhrushev, Hans H. Wandall

**Affiliations:** † Copenhagen Center for Glycomics, University of Copenhagen, Copenhagen 2200, Denmark; ‡ Department of Odontology, University of Copenhagen, Copenhagen 2200, Denmark

## Abstract

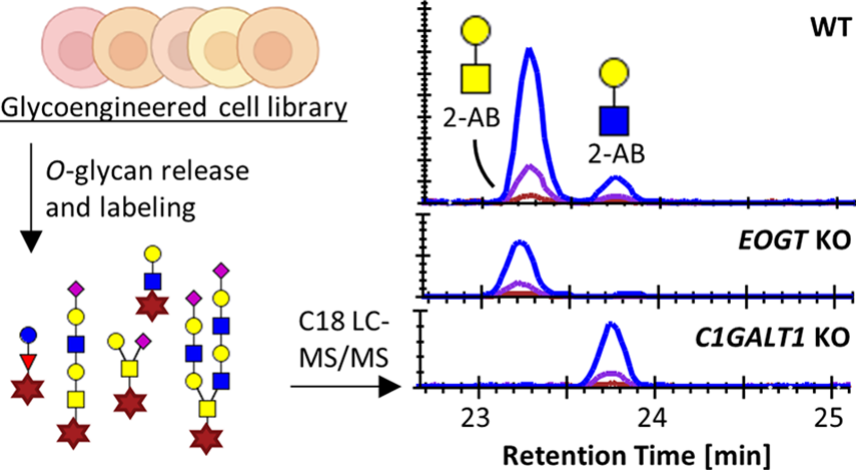

*O*-Glycosylation is an omnipresent modification of the human proteome affecting many cellular functions, including protein cleavage, protein folding, and cellular signaling, interactions, and trafficking. The functions are governed by differentially regulated *O*-glycan types and terminal structures. It is therefore essential to develop analytical methods that facilitate the annotation of *O*-glycans in biological material. While various successful strategies for the in-depth profiling of released *O*-glycans have been reported, these methods are often limitedly accessible to the nonspecialist or challenged by the high abundance of *O*-glycan structural isomers. Here, we developed a high-throughput sample preparation approach for the nonreductive release and characterization of *O*-glycans from human cell material. Reducing-end labeling allowed efficient isomer separation and detection using C18 nanoliquid chromatography coupled to Orbitrap mass spectrometry. Using the method in combination with a library of genetically glycoengineered cells displaying defined *O*-glycan types and structures, we were able to annotate individual *O*-glycan structural isomers from a complex mixture. Applying the method in a model system of human keratinocytes, we found a wide variety of *O*-glycan structures, including *O*-fucose, *O*-glucose, *O*-GlcNAc, and *O*-GalNAc glycosylation, with the latter carrying both elongated core1 and core2 structures and varying numbers of fucoses and sialic acids. The method, including the now well-characterized standards, provides the opportunity to study glycomic changes in human tissue and disease models using rather mainstream analytical equipment.

## Introduction

Glycosylation of proteins is a post-translational modification orchestrated by hundreds of different enzymes, spawning a multitude of glycosylation types and structures.^1,2^ An important subclass of protein glycosylation in human tissue is *O*-glycosylation, targeting serine, threonine, and in rare cases, tyrosine residues. *O*-Glycan types are classified by their initiating monosaccharide, including *O*-Fuc (fucose), *O*-Man (mannose), *O*-Glc (glucose), *O*-Xyl (xylose), *O*-Gal (galactose), *O*-GalNAc (*N*-acetylgalactosamine), and *O*-GlcNAc (*N*-acetylglucosamine). Mature *O*-glycans consist of polysaccharide chains with varied and often branched structures expressed in a tissue- and differentiation-specific manner.^2^ The functions of *O*-glycans range from the protection of epithelial surfaces to regulation of protein cleavage and folding, and modulation of signaling and cell–cell and cell–matrix interactions.^1,2^

The urge to understand the differential expression, and to define *O*-glycan structure–function relationships, has accelerated the development of analytical strategies targeting these molecules in recent years.^3^ However, the efforts have been challenged by the absence of an enzyme for the unbiased release of *O*-glycans from their protein carrier as well as by the immense abundance of (isomeric) *O*-glycan structures.

While chemical release strategies based on reductive β-elimination have been indispensable for the analysis of free *O*-glycans by mass spectrometry (MS),^4−8^ the inherent protection of the reducing end *via* reduction prevents the functionalization of the glycans required for a diverse array of analytical techniques. Recently, great progress was made in the chemical release and labeling of *O*-glycans, keeping glycan degradation to a minimum.^9,10^ While these developments are important, it remains challenging to separate and characterize *O*-glycan isomers and to integrate the analysis in a high-throughput setup of complex samples often with limited tissue material available for the analysis.

Current methods for the structural annotation of *O*-glycans are largely based on liquid or gas phase separation (e.g., liquid chromatography (LC), capillary electrophoresis (CE), or ion mobility (IM)) in combination with exoglycosidase treatment and/or MS fragmentation.^3−8,11^ While the coanalysis of well-defined standards is key to the facile assignment of *O*-glycans, obtaining all standards needed to cover the full range of glycoforms is not trivial. Fortunately, most steps in glycan biosynthesis follow strict and rather well-defined pathways.^1^ Knowledge on and genetic manipulation of these pathways can be employed for the annotation of glycan structures.^12^ Recently, diverse arrays of glycan-engineered human cells were developed, which have the potential to be implemented as standards in analytical workflows.^13,14^

Here, we further developed the minimally destructive, nonreductive release of *O*-glycans from proteins in cell lysates and combined this with glycoengineered cells to establish standards needed for structural annotation. The method allows multiplexed sample preparation in a 96-well format as well as the sequential release of *N*- and *O*-glycans from the same sample. Uniform labeling of the glycan’s reducing end enables efficient C18 nanoLC–MS/MS analysis using a standard proteomics setup and features glycan isomer separation. Combining the method with the characterization of an array of glycoengineered cell lines resulted in the structural annotation of *O*-glycans derived from human keratinocytes, including the annotation of different *O*-glycan types and *O*-GalNAc core elongation. The method is well accessible for proteomics laboratories and easy to adapt to different types of samples, including tissues and biofluids.

## Experimental Section

### Chemicals and Samples

Details about the chemicals used can be found in the Supporting Experimental Section. All glycoengineered isogenic HEK293 cells used in this study (Table 1) are available as part of the cell-based glycan array resource.^13,15^ The N/TERT-1 immortalized human keratinocytes were kindly provided by James G. Rheinwald’s lab, Harvard Institute of Medicine, Brigham & Women’s Hospital,^16^ and the HaCaT keratinocytes were kindly provided by Norbert Fusenig and Petra Boucamp, DKFZ, Heidelberg.^17^ The keratinocytes knockout (KO) library (Table 1) was generated using CRISPR/Cas9 technology as described previously (Supporting Experimental Section).^14,18^ For glycan analysis, the cell pellets were resuspended in lysis buffer (∼5 × 10^5^/25 μL, unless stated otherwise) and lysed using a sonic probe for 1.5 min with 5 s on/off cycles and 60% power. Next, the lysed material was incubated at 60 °C for 30 min with agitation. Fetuin from fetal bovine serum was solubilized in lysis buffer at a concentration of 1 μg/μL (optimization of release conditions) or 0.2 μg/μL (as quality control in sample batches) and further treated like the cell material. 2-Aminobenzamide (2-AB)-labeled GlcNAc and GalNAc standards were prepared as described below and used in a final concentration of 25 fmol/μL.

**Table 1:** Cell Types and Their Glycan-Engineered Variants Subjected to *O*-Glycan Profiling

cell type	genetic modification	expected *O*-glycan phenotype^1,13^
HEK293	wild type	
	*C1GALT1* KO	loss of *O*-GalNAc core1 and core2
	*GCNT1*, *ST6GALNAC2*/*3*/*4*, *ST3GAL1*/*2* KO	loss of *O*-GalNAc core2, no core1 sialylation
	*GCNT1*, *ST6GALNAC2*/*3*/*4* KO	loss of *O*-GalNAc core2, no GalNAc-linked sialylation
	*GCNT1*, *ST6GALNAC2*/*3*/*4* KO, ST6GALNAC3 KI	loss of *O*-GalNAc core2, enhanced GalNAc-linked sialylation
	*COSMC* KO, B3GNT6 KI	loss of *O*-GalNAc core1 and core2, enhanced core3 formation
	*GCNT1*	loss of *O*-GalNAc core2
	*B4GALT1*/*2*/*3*/*4* KO	loss of type 2 LacNAc elongation
	*ST3GAL1*/*2*/*3*/*4*/*5*/*6*, *ST6GAL1*/*2* KO	loss of galactose-linked sialylation
		
N/TERT-1	wild type	
	*POFUT1* KO	reduced *O*-fucose type
	*POGLUT1* KO	reduced *O*-glucose type
	*EOGT* KO	loss of extracellular *O*-GlcNAc type
	*C1GALT1* KO	loss of *O*-GalNAc core1 and core2
	*GCNT1* KO	reduced *O*-GalNAc core2
		
HaCaT	wild type	
	*POMT1* KO	reduced *O*-mannose type
	*POMT1/2* KO	reduced *O*-mannose type

### Protein Blotting and *N*-Glycan Release

Cell lysate proteins or protein standards were blotted on the polyvinylidenefluoride (PVDF) membranes (MultiScreenHTS IP Filter Plate, 0.45 μm, Millipore) as described previously^4^ (Supporting Experimental Section). Briefly, 25 μL of each sample was loaded on the PVDF membranes and *N*-glycans were released using 2 U PNGase F in 30 μL of water. *N*-Glycans were eluted from the membrane in a total volume of 150 μL of water and dried at 30 °C in a vacuum concentrator.

### Optimization of *O*-Glycan Release and Labeling

The de-*N*-glycosylated proteins on the PVDF membrane were rewetted with 10 μL of water, and 15 μL of release reagent (33% hydroxylamine and 33% 1,8-diazabicyclo(5.4.0)undec-7-ene (DBU) in water) was added. The samples were shaken for 30 s at room temperature and incubated for 1 h at 37 °C in a moisture box. During the optimization of the reaction, samples were incubated with final concentrations of 20% hydroxylamine and 20% DBU, 10% hydroxylamine and 40% DBU, or 0% hydroxylamine and 20% DBU for 1 h at 37 °C.^9^ The pH of these conditions was determined using pH paper to increase between 11 and 14 with decreasing concentrations of hydroxylamine, independent of the DBU concentration. The *O*-glycans were recovered from the membrane by 2 min centrifugation at 1000*g*, and 1 mL of acetonitrile (ACN) containing 2 mg of magnetic hydrazide beads (MagSi-S Hydrazide beads 1 μm, magtivio B.V., Nuth, The Netherlands) was added. For the optimization of the hydrazide purification, 0.5, 1, 2, 4, or 6 mg of beads was used per sample. The samples were incubated with the beads for 5 min at room temperature and placed on a magnetic separator for 5 min. After two washes with 200 μL of ACN, the *O*-glycans were eluted from the hydrazide beads in 50 μL of 2-AB reagent (500 mM 2-AB, 116 mM 2-methylpyridine borane complex in 45:45:10 metanol:water:acetic acid). The 2-AB labeling reaction was incubated for 2.5 h at 50 °C, 1 mL of ACN was added, and the glycans were purified by cotton hydrophilic interaction chromatography (HILIC) solid phase extraction (SPE) and eluted in 50 μL of water.^19^ The labeled *O*-glycans were further purified using porous graphitic carbon (PGC) SPE (Supporting Experimental Section).^4^ Finally, the samples were dried and reconstituted in 20 μL of water for MS analysis.

### Liquid Chromatography–Mass Spectrometry

Two microliters per sample (10% of total) was injected per analysis. The glycans were separated by nanoflow liquid chromatography (nanoLC) using a single analytical column setup packed with Reprosil-Pure-AQ C18 phase (Dr. Maisch, 1.9 μm in particle size, 19–21 cm in column length) in an EASY-nLC 1200 UHPLC (Thermo Fisher Scientific) using a PicoFrit Emitter (New Objectives, 75 μm in inner diameter). The emitter was interfaced to an Orbitrap Fusion Lumos MS (Thermo Fisher Scientific) via a nanoSpray Flex ion source. Details on the LC–MS/MS methods can be found in the Supporting Experimental Section.

### Data Analysis

MS1 feature detection in the raw files was performed using the Minora Feature Detector node in Thermo Proteome Discoverer 2.2.0.388 (Thermo Fisher Scientific Inc.). For parameters and filtering, see the Supporting Experimental Section. The [M + H] values of the resulting features were imported into GlycoWorkbench 2.1 (build 146)^20^ and matched to glycan compositions with 0 to 8 hexoses, 0 to 8 *N*-acetylhexosamines, 0 to 3 fucoses, 0 to 4 *N*-acetylneuraminic acids, and a 2-AB label. An additional matching was performed to glycan compositions with 0 to 6 hexoses, 0 to 6 *N*-acetylhexosamines, 0 to 2 fucoses, 0 to 2 *N*-acetylneuraminic acids, 0 to 3 pentoses, and a 2-AB label. The complete list of identified compositions was imported into Skyline 21.1.0.146 (ProteoWizard) using the Molecule Interface. For settings and quality control parameters, see the Supporting Experimental Section. The MS/MS spectra were manually assigned for each MS1 feature in at least one sample (Supporting Figures S1 and S2 and Supporting Table S1). MS1-assigned glycans that were not targeted for MS fragmentation during the first DDA run were specifically targeted in the second run of a selected set of samples. Finally, total area normalization was performed for the complete set of glycans as well as for the subset of *O*-GalNAc glycans to obtain the relative abundances per glycan in each sample.

## Results and Discussion

We developed a high-throughput sample preparation method for the analysis of *O*-glycans released from proteins in complex mixtures by reversed-phase LC–MS and introduced glycoengineered cells as standards for their structural assignment.^13,14^ The sample preparation is partly based on the integrated release of *N*- and *O*-glycans from cells and tissues after protein immobilization on a PVDF membrane in a 96-well plate format as described by Zhang et al.,^4^ in combination with the nonreductive, minimally destructive chemical release of *O*-glycans as described by Kameyama et al.^9^ Importantly, the nonreductive β-elimination used for the liberation of the *O*-linked glycans allows for functionalization of the reducing ends of the glycans.^9^ We exploited this feature by labeling the released *O*-glycans with 2-AB, which facilitated isomer separation on a C18 nanoflow LC column.

### Optimization of *O*-Glycan Release by Nonreductive β-Elimination

First, the glycoproteins from biological material were immobilized, and *N*-glycans were enzymatically released, a procedure that was adapted from previous reports.^4,6,21^ Next, *O*-glycans were chemically released by β-elimination at a high pH (pH 11) using hydroxylamine to reversibly protect the reducing end of the glycans from peeling.^22^ Conventional β-elimination protocols prevent peeling by the permanent reduction of the reducing end during the release of the *O*-glycans. While effective, such approaches limit further labeling of the glycans.^3^ Recently, a protocol was developed for the efficient release of *O*-glycans in only 20 min at 50 °C using the organic super base DBU in combination with hydroxylamine.^9^ To integrate this protocol with the PVDF immobilization of glycoproteins, the incubation temperature was reduced to 37 °C, while the incubation time was prolonged to 1 h.^9^ Different concentrations of hydroxylamine and DBU were evaluated using fetuin as a standard, while the peeling rates and total signal intensity were monitored (Supporting Figure S3). The four most abundant *O*-GalNAc glycans reported on fetuin are core1 glycans with the compositions H1N1, H1N1S1, and H1N1S2 and the core2 glycan H2N2S2 (where H indicates hexoses, N indicates *N*-acetylhexosamine, and S indicates *N*-acetylneuraminic acid).^23^ All structures were found in the current analyses. Peeling of sialylated core1 and core2 glycans results in the disaccharide H1S1, of which the relative abundance was monitored and compared to the total sum of identified glycans. In the absence of hydroxylamine, H1S1 represented over 90% (standard deviation, ±0.6%) of the quantified glycans. Using 10 or 20% hydroxylamine reduced the peeling drastically to 17% (±0.9%) and 9% (±0.5%), respectively. The current peeling rate is slightly higher than the 3% reported for the original method^9^ and in the same range as reported for reductive β-elimination protocols (0–10%).^3^ It is at the lower side of the range reported for other nonreductive β-elimination approaches (0–60%).^3^

### Optimization of *O*-Glycan Purification by Hydrazide Beads

Next, the released *O*-glycans were recovered from the PVDF membrane and enriched from the reaction mixture *via* their reversible binding to magnetic hydrazide beads. The optimal amount of hydrazide beads was investigated using HaCaT wild-type (HaCaT^WT^) cells, for which the relative abundance between glycoforms as well as the absolute signal intensity was monitored (Supporting Figure S4). While the lower amounts of beads resulted in skewing of the profile, underrepresenting the smaller glycoforms, a plateau was reached using ≥2 mg of beads, with the maximum signal intensity obtained using 2 mg of beads. While the lower intensity with less beads can be explained by the limited capacity of the beads (something that is also supported by the skewed glycosylation profile at lower amounts), the intensity loss at higher amounts is likely caused by sample losses due to an increase in void volume with sample handling using more than 2 mg of beads in the current format. After hydrazide capture and washing, the *O*-glycans were directly eluted with the 2-AB labeling reagent. Tagging of the glycans with an aromatic label enhances both reversed-phase retention and protonation/desolvation in the ion source.^24^ During the elution, different aldehyde reactive labels can be introduced, which might be beneficial to enhance MS or fluorescence sensitivity,^24−26^ allowing separation on different platforms such as HILIC or CE,^26,27^ or to introduce isotope labels for multiplexed analysis.^28,29^ More hydrophobic labels carrying tertiary amines, such as procainamide, have previously been optimized for improved MS sensitivity^24,26^ and can alternatively be used in the current workflow. The C18 separation behavior of these labels has however yet to be determined. HILIC SPE was suggested as an alternative to the hydrazide cleanup.^9^ However, small and nonlabeled glycans have a lower retention on HILIC materials than the larger hydrophilic structures, introducing the selective loss of mono- and disaccharides. Furthermore, the hydrazide beads allow the direct elution in the labeling reagent.

### Repeatable *O*-Glycan Profiling of Total Cell Lysates

**Figure 1:**
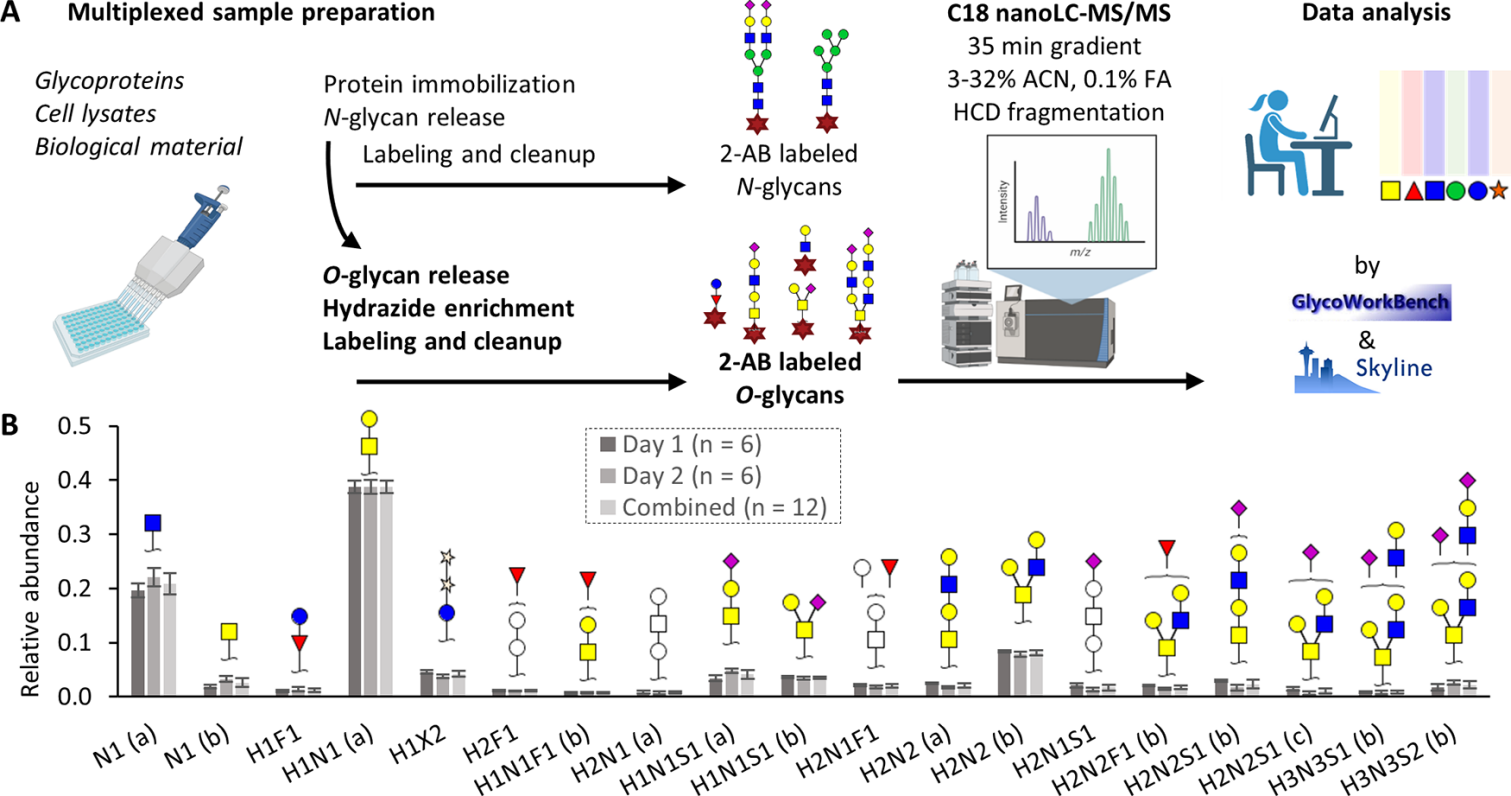
Intra- and interday repeatability of the optimized method. (A) Workflow of the optimized method. A scheme detailing the chemistry used for the *O*-glycan release, hydrazide enrichment, and 2-AB labeling can be found in Supporting Figure S5. (B) The *O*-glycans from approximately 2.5 × 10^5^ HaCaT^WT^ cells were released, labeled, and analyzed by C18 LC–MS/MS for two times with six technical replicates on two successive days. Displayed are average relative intensities for the glycans with a relative abundance above 1% per day, with error bars representing the standard deviations. Graphics in panel (A) were created using https://biorender.com/. H, hexose; N, *N*-acetylhexosamine; F, fucose; S, *N*-acetylneuraminic acid.

The complete protocol (Figure 1A and Supporting Figure S5) from cell lysis to LC–MS/MS analysis was applied on 12 HaCaT cell pellets containing approximately 2.5 × 10^5^ cells each, divided over two successive days (six samples each day). All steps were performed in a 96-well plate format using 12-channel pipets for efficient sample handling and could be completed in 1.5 working days. This resulted in the identification of 15 *O*-glycan compositions with a relative intensity above 1% and 19 different structures, considering the C18-separated isomers (Supporting Table S2). Relative quantification of the 19 structures (elaborated structural annotation is described in the sections below) resulted in glycosylation profiles with high intra- and interday repeatability (Figure 1B). The highest abundant glycan, H1N1, showed a relative abundance of 39% with a coefficient of variation (CV) of 3% over 2 days. All glycans with a relative abundance above 5% featured CVs below 10%, while the average CV of the glycans with relative abundances ≤5% and ≥1% was 25% (Supporting Table S2). These values are comparable to the performances previously described for comprehensive *N*- and *O*-glycan analysis by MS.^4,30,31^ The complementary HILIC and PGC SPE of the 2-AB-labeled *O*-glycans derived from biological material are key to remove interferences and make the samples compatible with the C18 LC–MS analysis. For purified glycoproteins, a sole HILIC SPE step usually suffices.^9^ The minimum sample input amount for the optimized method was about 1 × 10^5^ cells (evaluated for HEK293^WT^) or 1 μg of fetuin standard (Supporting Figure S6). As different cells/biological samples have different glycosylation characteristics, with HEK293 cells known to have a low glycosylation content,^32^ the minimum number of cells required will be specific for each cell type used. The sensitivity reported here is in accordance with previous reports for the high-throughput preparation of *O*-glycans using approximately 5 × 10^5^ cells per sample.^4^

### Structural Identification of (Isomeric) *O*-Glycans

We found in total 29 different *O*-glycan compositions corresponding to at least 51 structures in three different cell types: the widely used HEK293 cells and two human keratinocyte cell lines, N/TERT-1 and HaCaT (Supporting Tables S3 and S4). Annotation of the various structures was based on a combination of MS fragmentation, genetic glycoengineering of cells (Table 1), and literature knowledge of known biosynthetic pathways (Supporting Figure S7).^1,13^

#### C18 Separation of Monomeric *O*-GlcNAc and *O*-GalNac (Tn Antigen)

**Figure 2:**
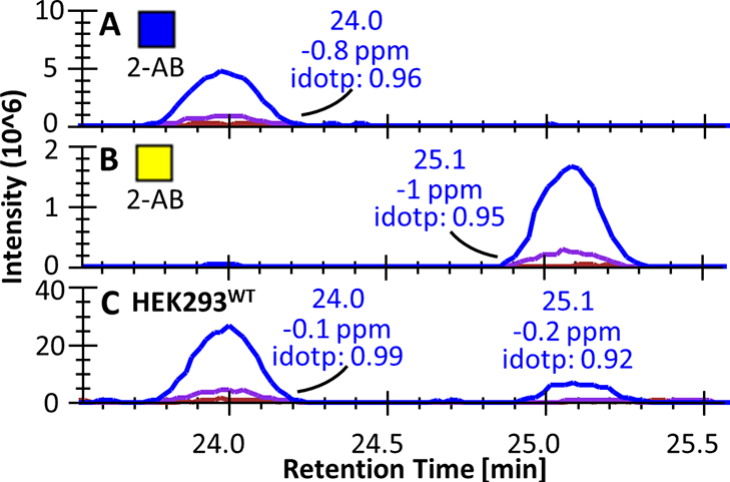
Chromatographic separation of two HexNAc isomers. The two HexNAc standards (A) GlcNAc and (B) GalNAc were 2-AB-labeled and analyzed separately. (C) Both species were found in the HEK293^WT^ total cell lysate. Idotp, isotopic dot product between theoretical and observed isotopic pattern. The blue, purple, and red lines correspond to the extracted ion chromatograms of the mono-isotopic mass, the second isotopologue, and the third isotopologue, respectively.

The optimized procedure allowed the labeling and retention of sugars as small as monomeric *N*-acetylhexosamine (HexNAc) residues derived from biological samples. Comparing the elution behavior of commercial GlcNAc and GalNAc standards to the HexNAcs observed in the cells aided the assignment of GlcNAc to the first eluting isomer and GalNAc to the second (Figure 2). The WT material of the investigated cells showed a 4 to 6 times higher abundance of monomeric GlcNAc as compared to GalNAc. While the separation of HexNAc isomers was shown before using, e.g., IM–MS,^11^ these epimers are notoriously difficult to analyze and cannot be discriminated using conventional methods for *O*-glycan analysis based on, e.g., permethylation and matrix-assisted laser desorption/ionization (MALDI)-MS.^30^ The separation of these isomers is biologically important as they represent distinct biosynthetic pathways. Whereas *O*-GlcNAc glycosylation can either be initiated in the nucleus/cytoplasm by OGT or in the endoplasmic reticulum by EOGT, *O*-GalNAc glycosylation is differentially regulated by 20 glycosyltransferases in the Golgi apparatus.^2^ Furthermore, cell surface-expressed monomeric *O*-GalNAc is a known tumor antigen (Tn antigen) and accurately monitoring this glycan is of high interest in cancer research.^33^

#### Identification of the *O*-Glycan Type

**Figure 3:**
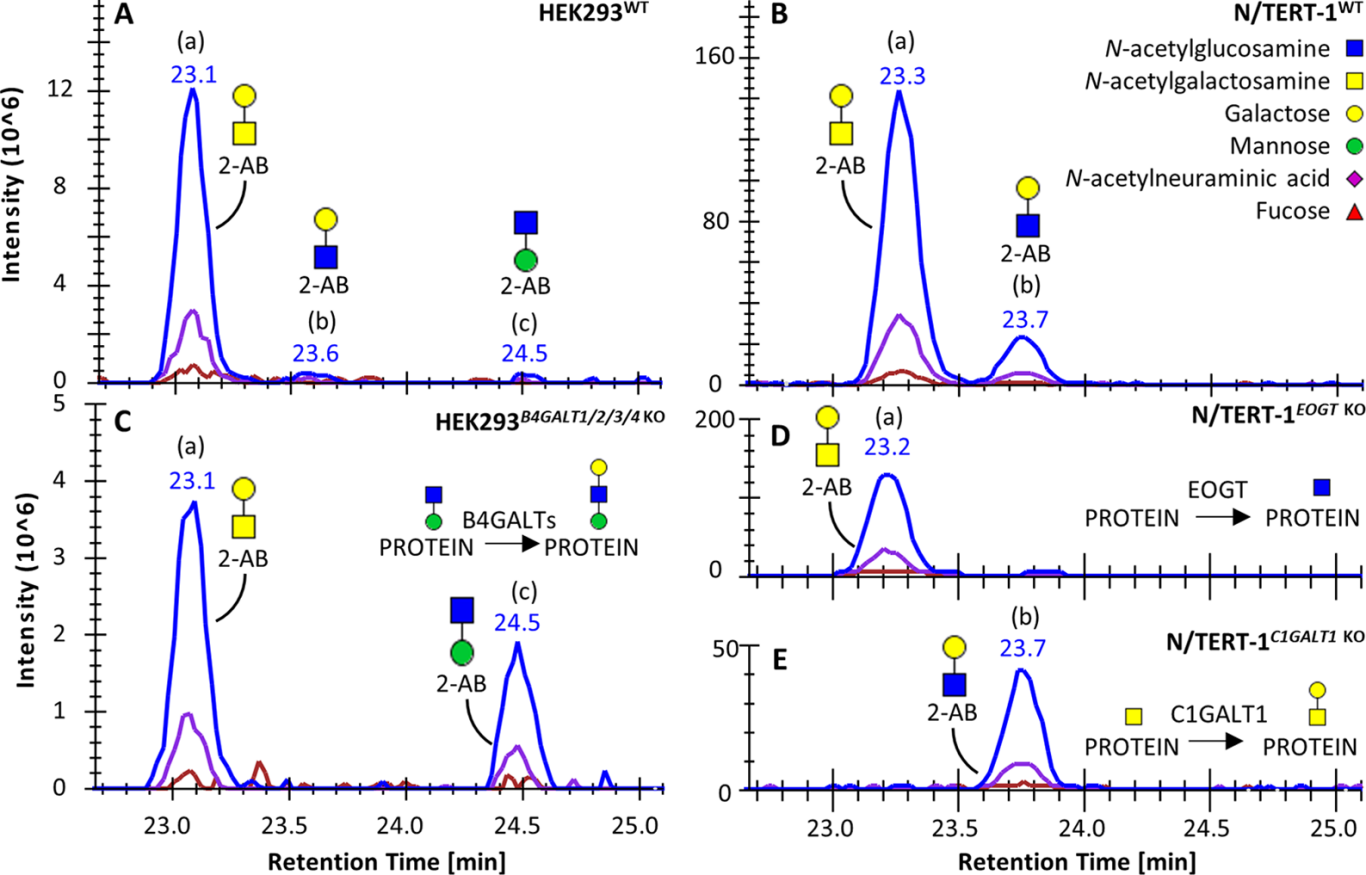
Extracted ion chromatograms for glycan composition H1N1 (*m*/*z* 504.219). Three differently eluting H1N1 isomers (a, b, and c) were observed in (A) the HEK293^WT^ and (B) N/TERT-1^WT^ samples. The (C) HEK293^*B4GALT1*/*2*/*3*/*4* KO^, (D) N/TERT-1^*EOGT* KO^, and (E) N/TERT-1^*C1GALT1* KO^ samples aided in the annotation of these isomers, in combination with MS fragmentation (Supporting Figures S1 and S2).

The first step in the annotation of the detected *O*-glycans is their assignment to an *O*-glycan type based on the initiating monosaccharide that was originally linked to the protein backbone. The various types of *O*-glycosylation include *O*-GalNAc, *O*-Fuc, *O*-GlcNAc, *O*-Man, *O*-Glc, *O*-Xyl, and *O*-Gal, all regulated by one or several specific enzymes in the secretory pathway.^1^ Higher-energy C-trap dissociation (HCD) fragmentation of the 2-AB-labeled sugars results in the Y_1_ ion, consisting of the initiating monosaccharide and the 2-AB label, to be among the most abundant fragments (Supporting Table S1). Fuc-, Hexose (Hex)-, and HexNAc-initiated glycans were found in the different cell lines and could be annotated to specific glycosylation types based on the glycan-engineered cell material (Supporting Tables S3 and S4). For example, the three isomers found for the disaccharide H1N1 (a, b, and c) were identified to belong to the *O*-GalNAc, *O*-GlcNAc, and *O*-Man pathways (Figure 3). While H1N1 (a) and H1N1 (b) were initiated by a HexNAc, H1N1 (c) was initiated by a hexose (Supporting Figures S1A,B and S2E,F). Furthermore, H1N1 (a) and (b) were abundant in the N/TERT-1^WT^ material, but H1N1 (a) was absent in the N/TERT-1^*C1GALT1* KO^, while H1N1 (b) was absent in the N/TERT-1^*EOGT* KO^. As C1GALT1 is responsible for *O*-GalNAc core1 synthesis and EOGT for the initiation of extracellular *O*-GlcNAc, H1N1 (a) and (b) were assigned to the *O*-GalNAc and *O*-GlcNAc pathways, respectively. Finally, H1N1 (c) was identified as an *O*-Man glycan, described to be abundant in the HEK293 material in the form of a trisaccharide, Gal-GlcNAc-Man-*O.*^34^ The abundance of H1N1 (c) increased in the HEK293^*B4GALT1*/*2/3*/*4* KO^. As these enzymes are responsible for the formation of type 2 LacNAc chains, this suggests that the observed disaccharide represents the GlcNAc-Man-*O* structure. Notably, H1N1 (b) can also be derived as a peeling product from core3, core4, and 3′-arm elongated core1 and core2 structures. As these types of glycans are limitedly present in the samples presented, we here consider the contribution of peeling negligible.

**Figure 4:**
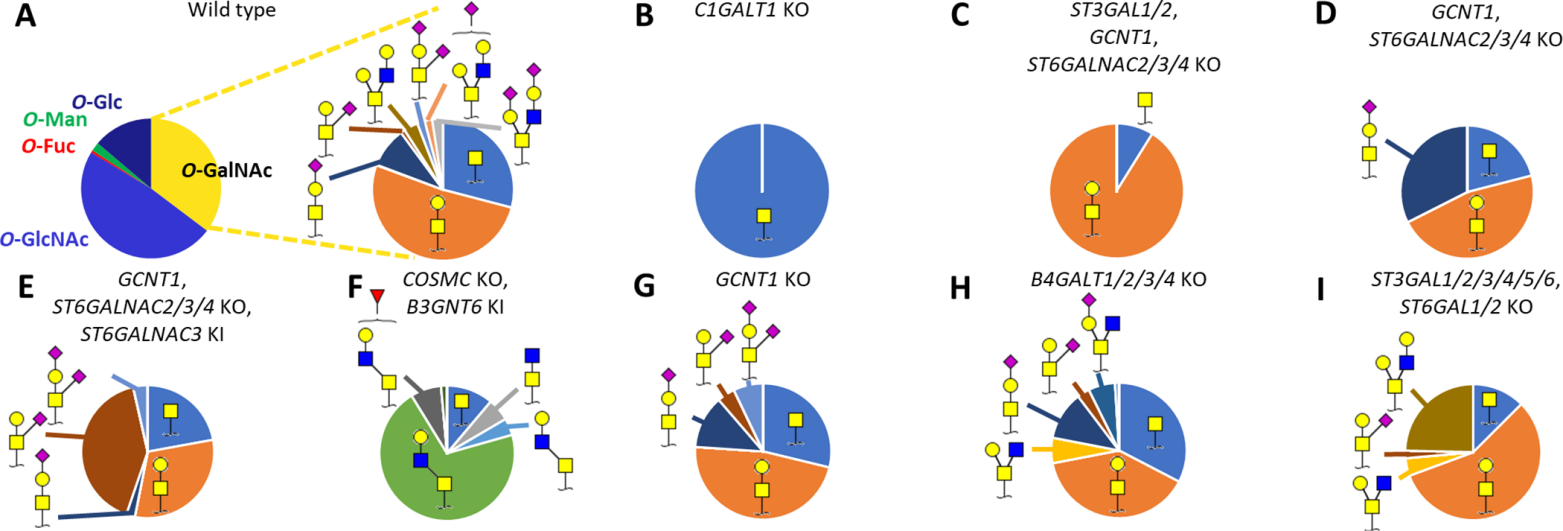
*O*-Glycan profiles of glycoengineered HEK293 total cell lysates. (A) Pie diagrams indicate the average relative intensity of *O*-glycan types and individual *O*-GalNAc glycoforms for HEK293^WT^ (*n* = 3). For the glycoengineered cells (B–I), only *O*-GalNAc glycans are displayed. The most abundant glycan structures are annotated per sample. Detailed information on all glycan abundances and structures can be found in Supporting Table S5 and Figure S8.

Using the approach described above, the 22 observed glycans in the HEK293 material were confidently assigned to an *O*-glycan type, showing the presence of *O*-GalNAc, *O*-Fuc, *O*-GlcNAc, *O*-Man, and *O*-Glc glycans (Figure 4). For the keratinocyte material, most of the 45 structures could be classified to one of these types as well, although some ambiguities remained as the current set of glycoengineered cells does not cover the complete array of glycan biosynthetic pathways (Supporting Table S4).

#### Annotation of *O*-GalNAc-Type Glycans

While the *O*-Fuc, *O*-GlcNAc, *O*-Man, and *O*-Glc glycans were only represented by one to three different structures, *O*-GalNAc-type glycosylation featured an abundance of (isomeric) glycans. The structures of the 15 different *O*-GalNAc glycans found in the HEK293 material were annotated by MS fragmentation in combination with the glycoengineered cell material that serves to predict the *O*-glycan structure from the gene KO/KI design (Figure 4 and Supporting Table S3). For example, the locations of the *N*-acetylneuraminic acid for the core1 compositions H1N1S1 (a) and (b) were assigned to the galactose and GalNAc, respectively, based on the diagnostic Y ion at *m*/*z* 633.262 in the fragmentation spectrum of H1N1S1 (b), representing the *N*-acetylneuraminic acid linked to the core GalNAc (Supporting Figure S1I,J). Furthermore, H1N1S1 (b) was absent in the HEK293^*GCNT1*, *ST6GALNAC2/3/4* KO^ material (eliminating the sialyltransferase genes related to GalNAc-linked sialylation) and recovered or enhanced in the same genetic background but with *ST6GALNAC3* KI (Figure 4D,E). Core2 *O*-GalNAc glycans were assigned based on their absence with the KO of the core2 synthase GCNT1, while (elongated) core3 structures, not observed in other HEK293 samples, emerged in the core3-enhanced cells (*B3GNT6* KI) (Figure 4F,G). The depth of identification for the *O*-glycans found in the HEK293^WT^ material exceeded other reports on this cell type, which were based on MALDI-MS and therefore omit the separation of isomers yet reporting the same compositional findings.^32^

**Figure 5:**
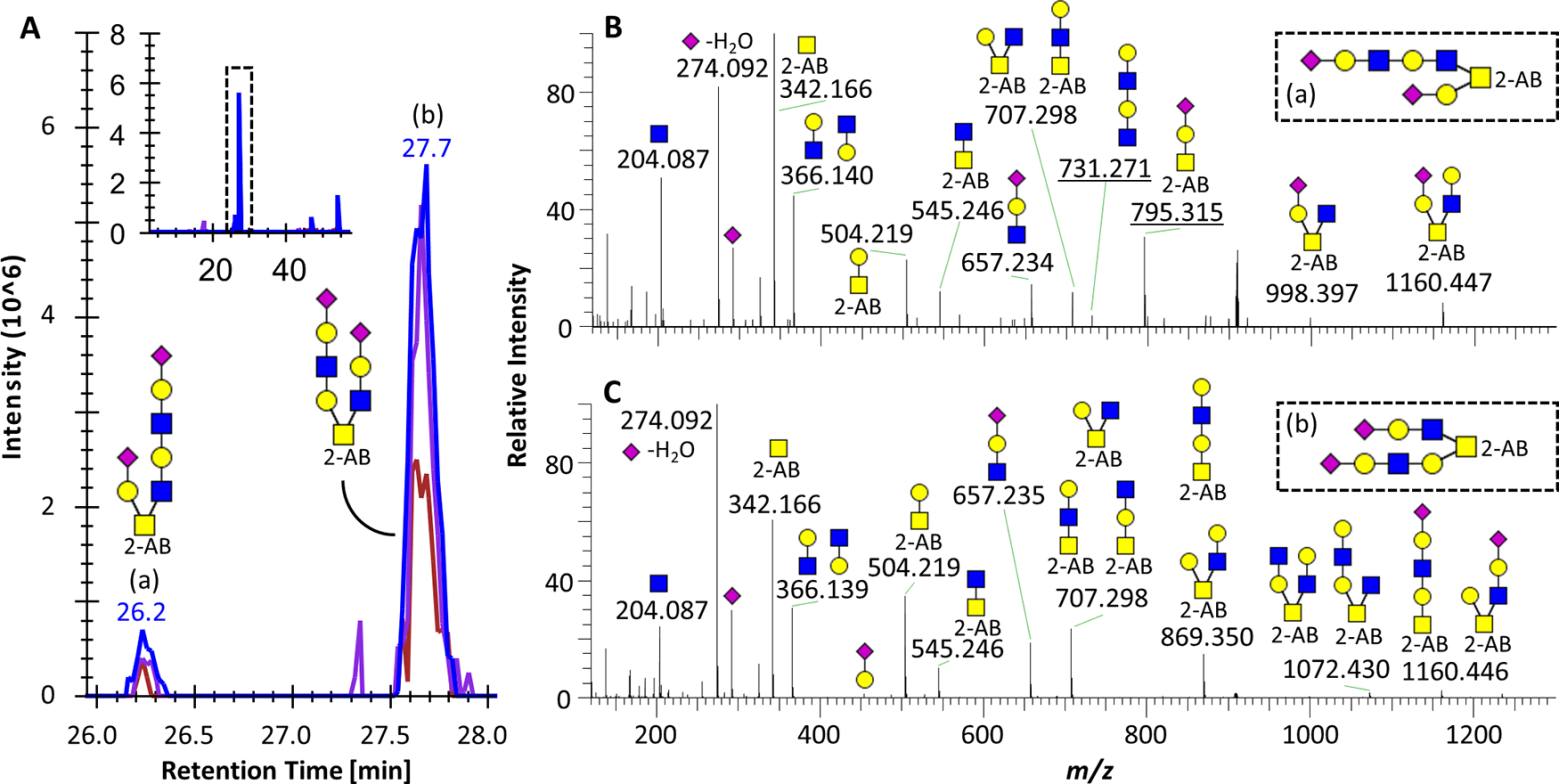
Structural characterization of two elongated *O*-GalNAc core2 isomers. (A) EICs for glycan composition H3N3S2 (*m*/*z* 908.841, 2+) in the HaCaT^WT^ sample, indicating the presence of two isomers (a and b). The inset shows the full elution range, highlighting the retention time area of the two isomers. (B) MS/MS spectrum of the first eluting species. (C) MS/MS spectrum of the second eluting species. All annotated peaks are 1+, glycan cartoons represent B and Y ions, and underlined *m*/*z* values in panel (B) indicate the diagnostic ions that allow the differentiation between the two structures.

The keratinocytes featured more complex *O*-GalNAc-type glycosylation, of which the cores were largely assigned using the *O*-GalNAc core1 and core2 KO and the HEK293 core3 KI material as standards (Supporting Tables S4 and S6 and Figures S9 and S10). Notably, the N/TERT-1^*GCNT1* KO^ did not completely abolish core2 *O*-GalNAc glycans as also GCNT4 is expressed in keratinocytes.^35^ Furthermore, structural features were derived from MS fragmentation as exemplified for the isomeric variants of the glycan composition H3N3S2 (a and b, Figure 5). While both structures were suggested to be core2 *O*-GalNAc glycans by the presence of Y-ions at *m*/*z* 504.219 and 545.245, representing Hex-HexNAc-2-AB and HexNAc-HexNAc-2-AB, respectively, H3N3S2 (a) carried one of its *N*-acetylneuraminic acids directly on the galactose of the 1,3 branch (NeuAc-Hex-HexNAc-2-AB at *m*/*z* 795.315, Figure 5). Additionally, H3N3S2 (a) featured an oxonium ion at *m*/*z* 731.271, indicative of a LacNAc-elongated 1,6-branch. This ion was not present for H3N3S2 (b), making an extended 1,3-branch more likely. To further interrogate on the structure of these elongated glycans, glycoengineered material can be used that targets the synthesis of LacNAc repeats and sialic acid capping (Supporting Figure S7). Notably, a minority of the di-, tri-, and tetrasaccharides found in the keratinocyte material may be derived from peeling of the elongated structures,^3^ as we determined the peeling rate to be just below 10% using fetuin core1 glycans. The potential interference of *N*-glycans with identical monosaccharide compositions to the *O*-glycans was excluded based on the sequential analysis of the 2-AB-labeled *N*-glycans derived from the same samples (Supporting Figure S11). While no direct comparison was made between the depth of structural identification between the current method and state-of-the-art approaches based on PGC LC and negative mode MS/MS, we observed a similar extent of separation between LacNAc, fucose, and sialic acid locations and improved identification of isomeric mono- and disaccharides.^6,21,36^ Positive mode HCD as employed in the current study resulted in limited information regarding glycosidic linkages as compared to negative mode fragmentation or MS/MS of permethylated glycans.^34,36^ The latter can in the future be addressed by the inclusion of a wider variety of genetically engineered glycan standards.

### Methodological Considerations

In this study, the implementation of genetically glycoengineered cells aided in the assignment of many (isomeric) *O*-glycan structures. These now well-defined cellular standards can in the future be coanalyzed with new samples of interest for the facile annotation of the covered *O*-glycans. Often, the combination of retention time, accurate mass, and isotopic pattern matching to a standard is enough for the confident annotation of glycoforms. This allows for more rapid glycoform identification as compared to manual MS/MS annotation while, at the same time, delivering complementary information. As compared to exoglycosidase approaches, the use of glycoengineered standards provides a wider coverage of possible glycosylation features. Furthermore, well-characterized standards can be measured in parallel to the samples of interest, not requiring the consumption of possibly precious samples to perform multiple exoglycosidase treatments. All cell materials used in this study, as well as other glycoengineered variants, are available to the community upon request.^13−15^ Notably, to forestall variations in retention time within or between measurement sequences, the reference sample should be included repeatedly. To further enhance the accessibility of the standards, specific isolated proteins, such as mucins, can be produced from the genetically engineered cells,^37^ or retention times of the annotated structures can be converted to glucose units. The latter has been abundantly used during HILIC-fluorescence profiling of glycans^38^ and has also been proven successful for the standardization of permethylated glycans analyzed by C18 LC–MS.^34^

While the current work did not aim to cover the complete wealth of *O*-glycan structures found in human glycobiology, we showed that glycan-engineered standards are a way into understanding and assigning glycan structures present in a biological system of interest. To cover a wider range of *O*-glycan structures, alternative cell systems might be considered, e.g., neuronal or colorectal cells,^10,36^ as well as different genetically engineered cells targeting, for example, fucosyl- and sialyltransferases.

### Conclusions

We developed an easy-to-implement method for the characterization of *O*-glycans from cells and tissues. The individual methodological components were here for the first time combined into a method that features high-throughput and accessible sample preparation for *O*-glycans from biological material, facilitating isomer separation by C18 nanoLC-MS/MS. The structural characterization of isomeric *O*-glycans was aided by the implementation of genetically glycoengineered human cells. This material can in the future be used as a reference standard for facile annotation of glycan structures. We foresee the application of the presented method to study the potential change of specific glycan structures during tissue differentiation and disease development, as well as for a detailed analysis of the *in vivo* specificities of glycosyltransferases in a cellular system.

## Supporting Information Available

Additional experimental details, HCD-MS/MS spectra of the glycans, details on method optimization, and glycan relative abundances (PDF)
Details on the glycan structural annotation, relative abundances of the glycans, and overview of the mass spectrometry raw data (XLSX)

## Notes

The mass spectrometry data have been deposited to the ProteomeXchange Consortium via the PRIDE^39^ partner repository with the dataset identifier PXD029644.
