# Supplementary material for: In-Depth Profiling of O-Glycan Isomers in Human Cells Using C18 Nanoliquid Chromatography–Mass Spectrometry and Glycogenomics
Source: Anal Chem. 2022 Mar 4;94(10):4343–51. doi: 10.1021/acs.analchem.1c05068 (PMC8928149; doi:10.1021/acs.analchem.1c05068)
Supplement: Supplementary file 1 — ac1c05068_si_001.pdf [file ac1c05068_si_001.pdf]

Supporting Material for:

In-Depth Profiling of *O*-Glycan Isomers in Human Cells Using C18 nanoLC-MS and Glycogenomics

Noortje de Haan[a,*], Yoshiki Narimatsu[a], Mikkel Koed Møller Aasted[a], Ida SB Larsen[a], Irina N Marinova[a], Sally Dabelsteen[b], Sergey Y Vakhrushev[a], Hans H Wandall [a,*]

[a] Copenhagen Center for Glycomics, University of Copenhagen, Copenhagen, 2200, Denmark

[b] Department of Odontology, University of Copenhagen, Copenhagen, 2200, Denmark

* Corresponding authors; email: ndehaan@sund.ku.dk; hhw@sund.ku.dk

Content

Supporting Experimental Section.

Supporting Figure S1. HCD-MS/MS of HEK293 *O*-glycans.

Supporting Figure S2. HCD-MS/MS of keratinocyte *O*-glycans.

Supporting Figure S3. *O*-Glycans released from fetuin using different concentrations of hydroxylamine.

Supporting Figure S4. *O*-Glycans from HaCaT[WT] cells purified using different amounts of hydrazide beads.

Supporting Figure S5. Scheme representing the *O*-glycan release, clean-up and labeling.

Supporting Figure S6. *O*-glycans released from different amounts of input material.

Supporting Figure S7. Schematic overview of *O*-glycan biosynthesis.

Supporting Figure S8. Relative intensities of HEK293 *O*-glycans.

Supporting Figure S9. Relative intensities of N/TERT-1 *O*-glycans.

Supporting Figure S10. Relative intensities of HaCaT *O*-glycans.

Supporting Figure S11. Examples of isomeric *N*- and *O*-glycan compositions detected in N/TERT-1[WT].

Supporting Table S1. Regularly observed ions in HCD spectra of 2-AB labeled glycans.

Supporting Table S2. Intra- and interday repeatability of the optimized method.

Supporting Table S3. Identification and annotation of HEK293 *O*-glycans.

Supporting Table S4. Identification and annotation of keratinocyte *O*-glycans.

Supporting Table S5. Relative intensities of the HEK293 *O*-glycans.

Supporting Table S6. Relative intensities of the keratinocyte *O*-glycans.

Supporting Table S7. Description of the original data files uploaded to ProteomeXchange.

Supporting Experimental Section

Chemicals

LC-MS grade water, ethanol, methanol, acetic acid, acetonitrile (ACN), guanidine hydrochloride (GuHCl), dithiothreitol (DTT), Nonidet P-40 alternative, 50% hydroxylamine, 1,8-diazabicyclo(5.4.0)undec-7-ene (DBU), 2-methylpyridine borane complex (PB), *N*-Acetyl-D-glucosamine (GlcNAC) and *N*-Acetyl-D-galactosamine (GalNAc) standards, bovine fetuin and trifluoroacetic acid (TFA) were purchased from Merck (Darmstadt, Germany). Lyophilized PNGase F was obtained from Roche Diagnostics (Mannheim, Germany, MagSi-S Hydrazide beads 1 µm were from magtivio B.V. (Nuth, The Netherlands) and the 100% cotton thread (8/4) was from Bumbo (Brønderslev, Denmark). Bulk sorbent Carbograph was obtained from Grace Discovery sciences (Columbia, USA). Lysis buffer consisted of 50 mM Tris HCl, 100 mM NaCl and 1x cOmplete™ protease inhibitor (EDTA-free; Roche). 2-Aminobenzamide (2-AB), 80% acetonitrile with 0.1% formic acid (v/v), and water with 0.1% formic acid (v/v) (Optima™ LC/MS Grade) were from Thermo Fisher Scientific.

Keratinocyte KO library generation

N/TERT-1 and HaCaT KO libraries were generated using CRISPR/Cas9 technology by targeting *POMT1*, *POMT1* and *2* (for HaCaT), *POFUT1*, *POGLUT1*, *EOGT*, *C1GALT1* or *GCNT1* (for N/TERT-1) gene exons by validated gRNAs[1] or gRNAs predicted by GPP.[2] gRNAs were cloned using oligos (TAGC, Denmark) into lentiCRISPR-v2-Puro plasmid backbone (Addgene #52961).[3] Lentivirus was produced in HEK293T cells. For transfection, 200 µL OPTI-MEM (Gibco), 8 µL of 1 mg/mL PEI (Sigma), 0.8 µg LentiCRISPR-V2-gRNA plasmid, 0.6 µg pCMV-VSV-G plasmid (Addgene #8454), and 0.6 µg psPAX2 plasmid (Addgene #12260) were mixed and added to adherent HEK293T cells. After 24 h the transfection medium was replaced by K-SFM for N/TERT-1 transduction and DMEM 10% FCS (DMEM-10) for HaCaT transduction, and medium containing viral particles was collected 48/72 h post-transfection. Filtered virus-containing medium was mixed 1:1 with fresh complete K-SFM or DMEM-10 and polybrene (Sigma), and used to transduce N/TERT-1 cells or HaCaT cells overnight.[4] Selection of KO cell lines started 48-96 hours after transduction, with 1 µg/ml puromycin (Gibco). Cells were single cloned in 96-well plates and KO clones were identified by IDAA using ABI PRISMTM 3010 Genetic Analyzer (Thermo Fisher) and Sanger sequencing (GATC, Germany). Two to five clones were selected for each gene with out-of-frame indel formation. IDAA results were analyzed using Peak Scanner Software V1.0 (Thermo Fisher).[5]

Cell culture and harvest

HEK293 cells were cultured in suspension with serum-free F17 culture media (Invitrogen) containing 0.1% Kolliphor P188 (SIGMA) and 4 mM GlutaMax at 37 ˚C and 5% CO_2 under constant agitation (120 rpm). Cells were seeded at $5*10^5$ cells/mL, cultured for 2 days and harvested. Cultured HEK293 cells were washed twice with ice cold PBS, followed by centrifugation for 10 min at 400 xg and removal of the supernatant.

The keratinocytes were cultured on p100 culture plates. N/TERT-1 cells were cultured in K-SFM (Gibco) supplemented with 25 µg/mL BPE (Gibco), 0.2 ng/mL EGF (Thermo Scientific), and 0.3 mmol/L CaCl2 (Sigma) at 37 °C with 5% CO_2 as previously described.[6] N/TERT-1 cells were grown to 80% confluency and the medium was shifted to 1:1 vol/vol K-SFM/DF-K (DMEM/F12) supplemented with 25 µg/mL BPE, 0.2 ng/mL EGF, and 2 mM L-glutamine (Thermo Scientific). HaCaT cells were grown in DMEM with 10% FCS (Hyclone) to complete confluency. After cell culture, the keratinocytes were washed once (N/TERT-1) or

twice (HaCaT) with 5 mL ice-cold PBS and harvested by scraping in 2x 200 µL PBS, followed by centrifugation for 10 min at 600 xg and removal of the supernatant. All cell pellets were stored at -80 °C until resuspension in lysis buffer.

Protein blotting and N-glycan release

PVDF membrane filter plates were preconditioned by one wash with 200 µL 70% ethanol and two washes with 200 µL water using a plate centrifuge for 1 min at 500 xg, then the membranes were pre-wetted with 5 µL 70% ethanol. Cell lysate proteins or protein standards were blotted on the PVDF membranes by loading 25 µL sample per well followed by 20 min shaking at RT. Seventy-five microliter 7.7 M GuHCl and 6.7 mM DTT was added to each sample, shaken for 5 min at RT and incubated 30 min at 60 °C in a moisture box. After the samples cooled down to RT, they were spun for 2 min at 1000 xg and washed two times with 200 µL water, one time with 100 µL 0.01% NP40 and three times with 200 µL water using a plate centrifuge for 2 min at 1000 xg. Fifteen microliter water, containing 2 U PNGase F, was added to each sample and incubated for 15 min at 37 °C in the moisture box. Another 15 µL water was added and the samples were incubated overnight at 37 °C in the moisture box. The next day, *N*-glycans were eluted three times with 40 µL water and dried at 30 °C in a vacuum concentrator.

N-glycan labeling

The dried *N*-glycans were reconstituted in 50 µL 2-AB reagent and labeled and HILIC purified as described for the *O*-glycans. Ten microliter of the eluates was diluted in 10 µL water for MS analysis.

Cotton HILIC and PGC SPE

The cotton tips were prepared by loading 3 mm cotton strand (0.8-1 mg) into 200 µL pipet tips. The cotton tips were conditioned three times with 150 µL water and three times with 150 µL ACN. Forty times 150 µL sample was loaded by repeated pipetting, then the tips were washed with tree times 150 µL ACN and the glycans were eluted five times in 50 µL water. The labeled *O*-glycans were further purified using PGC SPE. The HILIC eluate was mixed with 30 µL 0.27% TFA (final concentration: 0.1%). PGC columns were prepared by washing a filter plate (96-well PP filter plate, Orochem Technologies (Naperville, IL, USA)) with 100 µL methanol and two times 100 µL water. Three milligram PGC material in methanol was loaded per filter and washed three times with 100 µL 80% ACN and 0.1% TFA and three times with 0.1% TFA using a plate centrifuge for 30 s at 500 xg. Eighty microliter sample was loaded on each column and incubated 10 min with agitation. The columns were washed two times with 100 µL 0.1% TFA and the *O*-glycans were eluted three times with 40 µL 60% ACN and 0.1% TFA. The samples were dried and reconstituted in 20 µL water for MS analysis.

Liquid chromatography-mass spectrometry

Two microliter per sample (10% of total) was injected per analysis. The glycans were separated by nano-flow liquid chromatography (nanoLC) using a single analytical column setup packed with Reprosil-Pure-AQ C18 phase (Dr. Maisch, 1.9 µm particle size, 19–21 cm column length) in an EASY-nLC 1200 UHPLC (Thermo Fisher Scientific) using a PicoFrit Emitter (New Objectives, 75 µm inner diameter). The emitter was interfaced to an Orbitrap Fusion Lumos MS (Thermo Fisher Scientific) via a nanoSpray Flex ion source. An 1 h method was used with a gradient from 3% to 32% of solvent B in 35 min, from 32% to 100% B in the next 10 min and 100% B for the last 15 min at 200 nL/min (solvent A: 0.1% formic acid in water; solvent

B: 0.1% formic acid in 80% ACN). A precursor MS scan (*m/z* 200-1700, positive polarity) was acquired in the Orbitrap at a nominal resolution of 120,000, followed by Orbitrap higher-energy C-trap dissociation (HCD)-MS/MS at a nominal resolution of 50,000 of the 10 most abundant precursors in the MS spectrum (charge states 1 to 4). A minimum MS signal threshold of 30,000 was used to trigger data-dependent fragmentation events. HCD was performed with an energy of 27% ± 5%, applying a 20 s dynamic exclusion window.

Data analysis

MS1 feature detection in the raw files was performed using the Minora Feature Detector node in Thermo Proteome Discoverer 2.2.0.388 (Thermo Fisher Scientific lnc.). Parameters for feature detection were as follows; Minimal trace length: 5; Minimal number of isotopes: 2; Maximum RT difference of isotope pattern multiplets: 0.05 min; No ID linking. The identified LC-MS features (defined by *m/z*, retention time (RT) and charge) were filtered based on RT (> 10 min, < 35 min) and charge (≥ 1, ≤ 4). Duplicate values were removed based on the rounding of all values to their third digit. The [M+H] values of the resulting features were imported into GlycoWorkbench 2.1 (build 146) [7] and matched to glycan compositions with 0 to 8 hexoses, 0 to 8 *N*-acetyl hexosamines, 0 to 3 fucoses, 0 to 4 *N*-acetyl neuraminic acids and a 2-AB label. An additional matching was performed to glycan compositions with 0 to 6 hexoses, 0 to 6 *N*-acetyl hexosamines, 0 to 2 fucoses, 0 to 2 *N*-acetyl neuraminic acids, 0 to 3 pentoses and a 2-AB label. The complete list of identified compositions was imported into Skyline 21.1.0.146 (ProteoWizard), using the Molecule Interface. Extracted ion chromatograms were generated for the first three isotopologues of each glycan, as well as for the MS/MS features at *m/z* 285.1445, 301.1394, 342.1660 and 271.1288, representing 2-AB-labelled fucose (Fuc; F), hexose (Hex; H), *N*-acetyl hexosamine (HexNAc; N), and pentose (Xyl; X), respectively. Chromatographic peaks were manually selected based on accurate mass (> -1 ppm, < 1 ppm), isotopic dot product (idotp; > 0.85) and a minimal signal intensity of $1*10^6$ in at least one of the samples and integrated for all samples. MS/MS spectra were manually assigned for each MS1 feature in at least one sample. Diagnostic ions used to assign structural features can be found in **Supporting Table 2**. MS1-assigned glycans that were not targeted for MS fragmentation during the first DDA run were specifically targeted in a second run in a select set of samples. Finally, total area normalization was performed for the complete set of glycans as well as for the subset of *O*-GalNAc glycans, to obtain the relative abundancies per glycan in each sample.

Supporting references

1. Narimatsu, Y.; Joshi, H. J., et al., A validated gRNA library for CRISPR/Cas9 targeting of the human glycosyltransferase genome. *Glycobiology* **2018,** *28* (5), 295-305.
2. Doench, J. G., CRISPR/Cas9 gene editing special issue. *FEBS J* **2016,** *283* (17), 3160-1.
3. Sanjana, N. E.; Shalem, O.; Zhang, F., Improved vectors and genome-wide libraries for CRISPR screening. *Nat Methods* **2014,** *11* (8), 783-784.
4. Ehrhardt, C.; Schmolke, M., et al., Polyethylenimine, a cost-effective transfection reagent. *Signal Transduction* **2006,** *6*, 179-184.
5. Konig, S.; Yang, Z., et al., Fast and Quantitative Identification of Ex Vivo Precise Genome Targeting-Induced Indel Events by IDAA. *Methods Mol Biol* **2019,** *1961*, 45-66.
6. Dickson, M. A.; Hahn, W. C., et al., Human keratinocytes that express hTERT and also bypass a p16(INK4a)-enforced mechanism that limits life span become immortal yet retain normal growth and differentiation characteristics. *Mol Cell Biol* **2000,** *20* (4), 1436-47.
7. Damerell, D.; Ceroni, A., et al., The GlycanBuilder and GlycoWorkbench glycoinformatics tools: updates and new developments. *Biol Chem* **2012,** *393* (11), 1357-62.

A H1N1 (a)

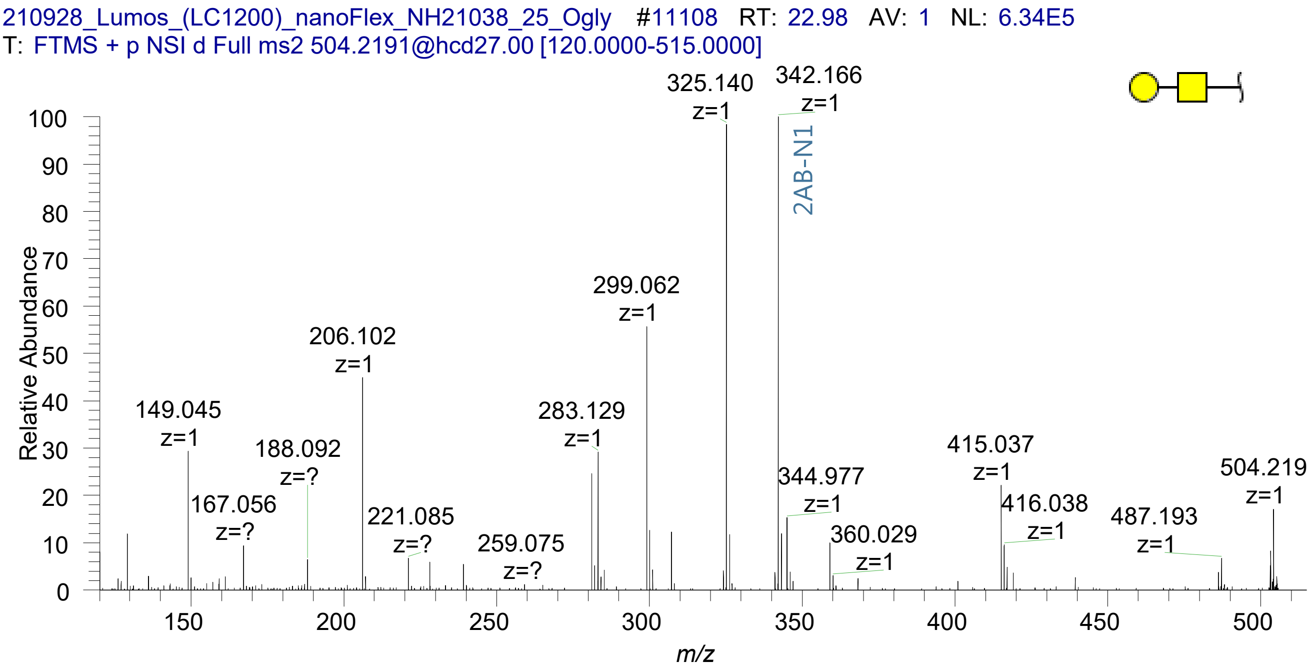

B H1N1 (c)

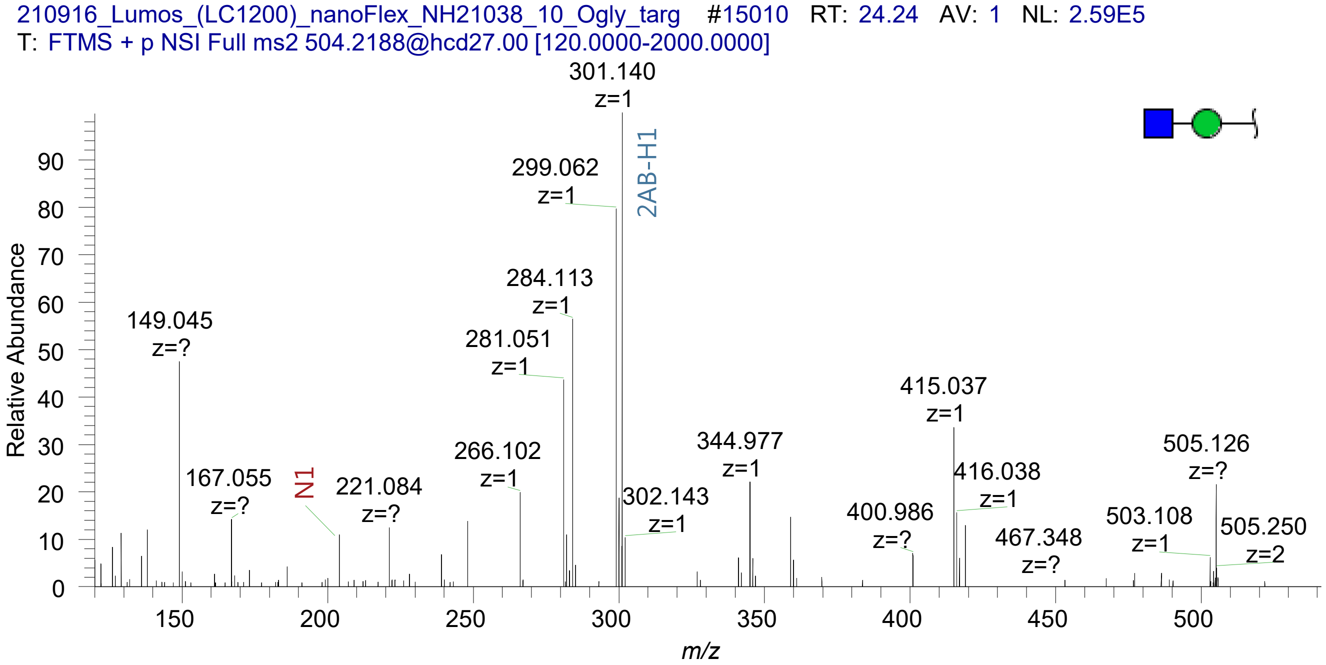

Supporting Figure S1. HCD-MS/MS of HEK293 *O*-glycans. Y-ions are indicated by compositions in blue, oxonium ions in red. H: Hexose, N: *N*-acetylhexosamine, F: fucose, S: *N*-acetylneuraminic acid, 2-AB: reducing end label. **Supporting Table S1** lists the ions used for annotation of the proposed structures. The depiction of the glycans indicates monosaccharide identity and connectivity, but not linkage. Additional evidence for the proposed depictions can be found in **Supporting Table S3 and S4**.

C N2

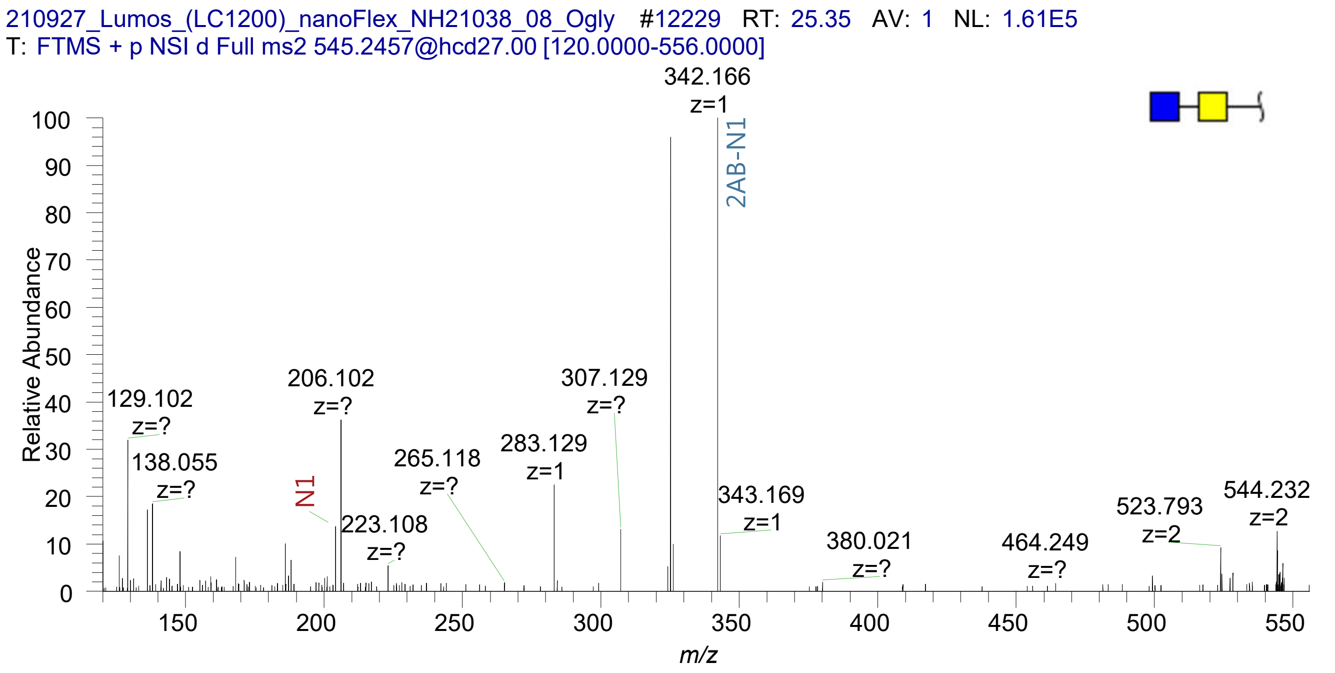

D H1X2

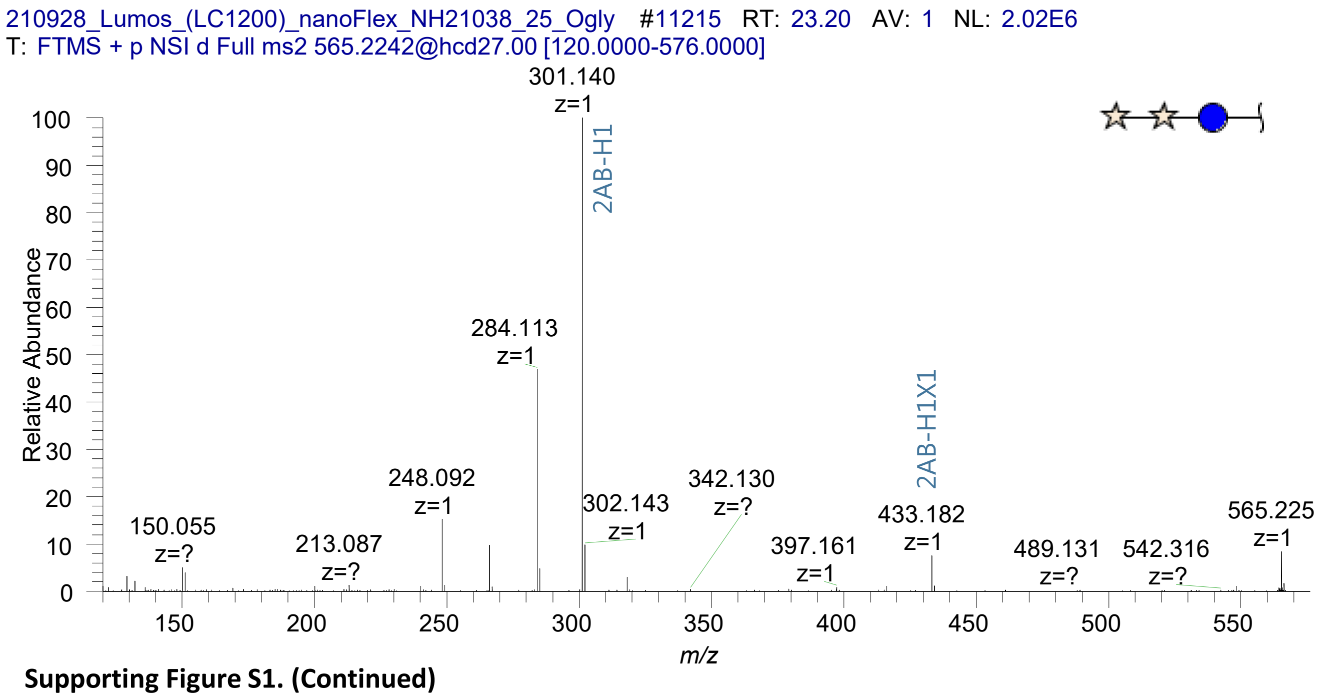

Supporting Figure S1. (Continued)

E H2N1 (b)

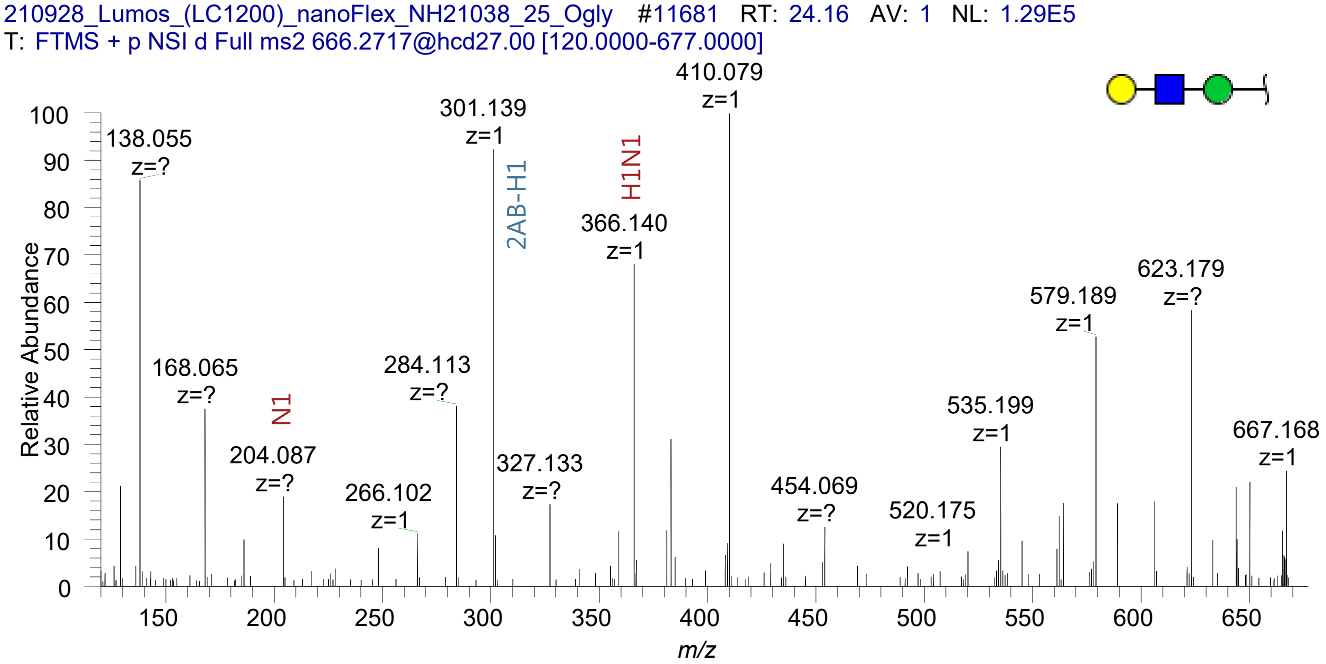

Supporting Figure S1. (Continued)

F H1N2 (a)

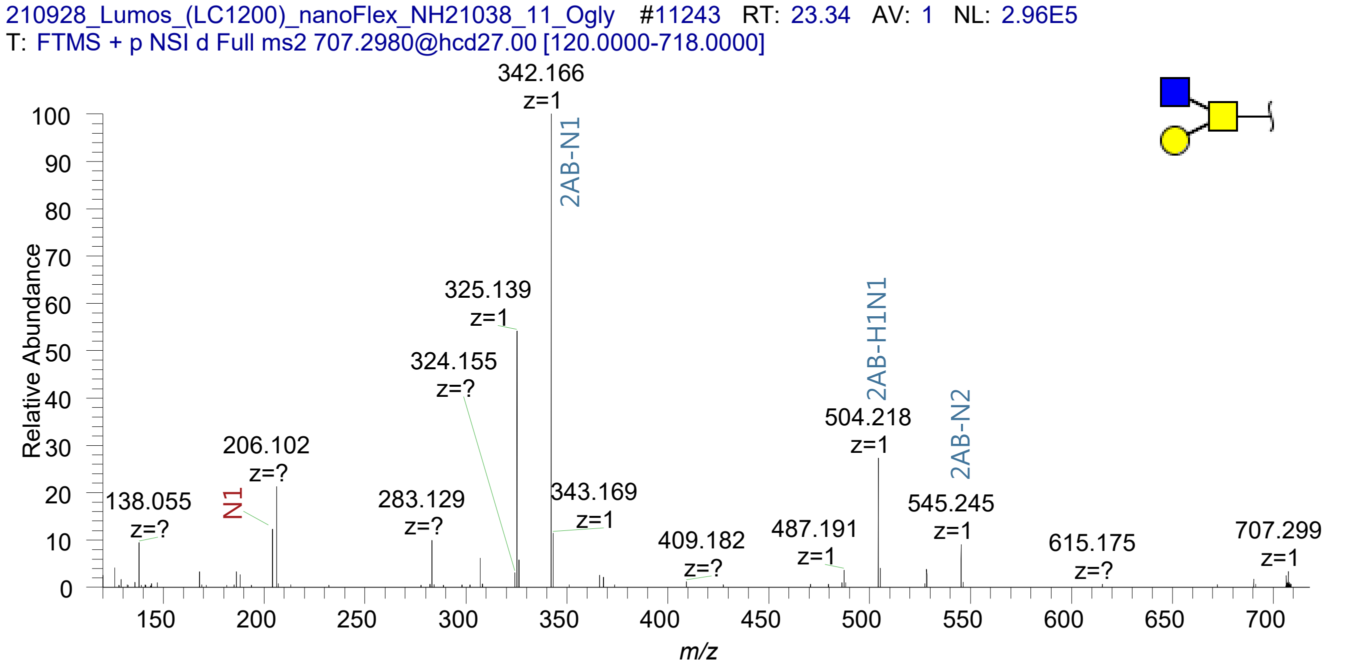

G H1N2 (b)

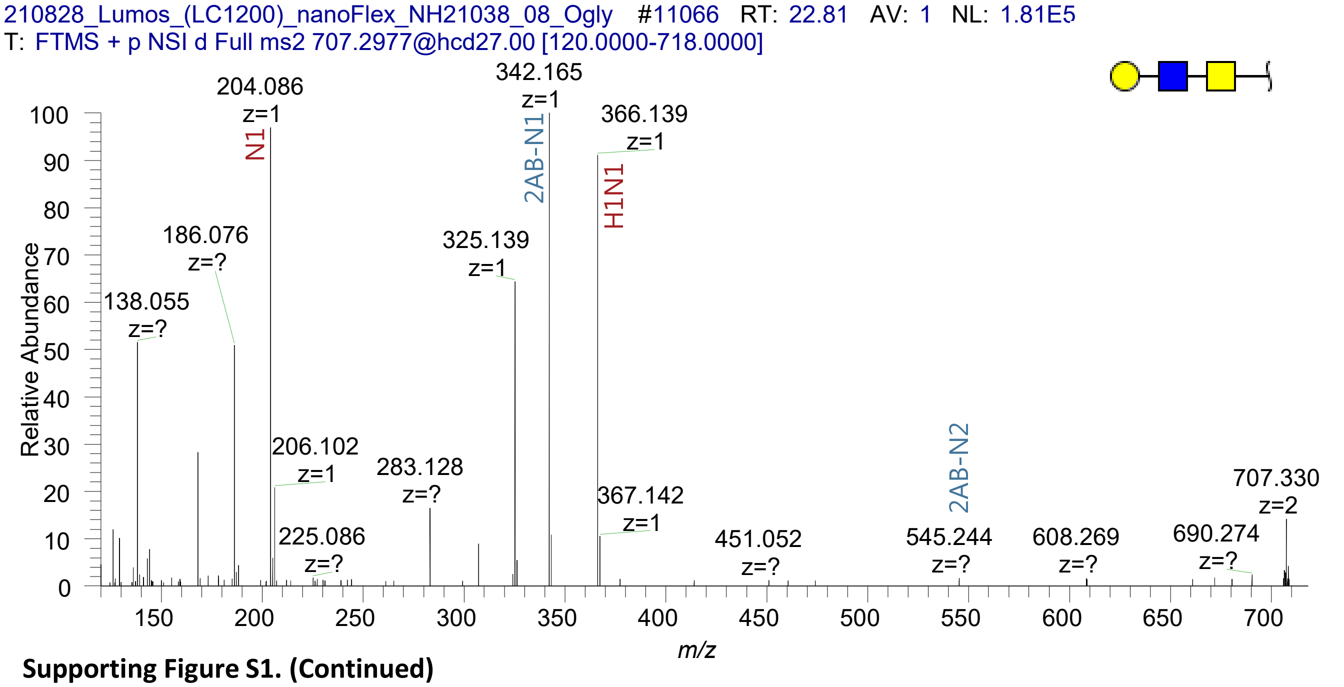

Supporting Figure S1. (Continued)

H H1N2 (c)

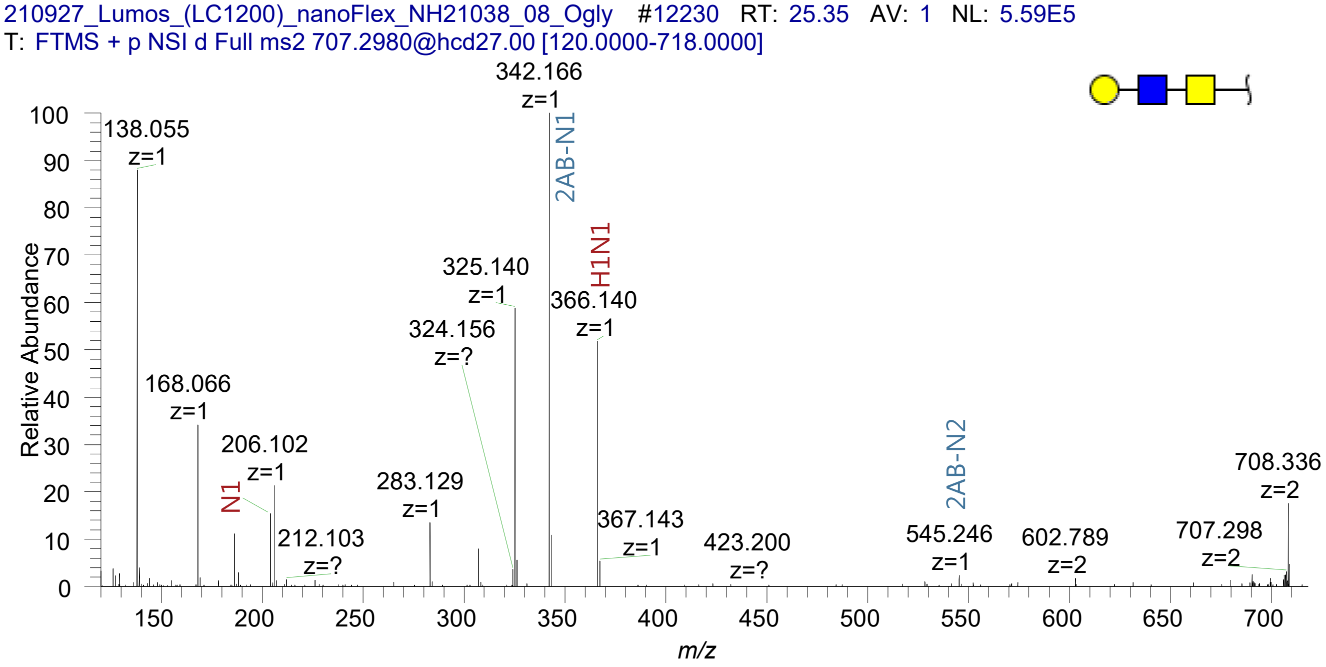

Supporting Figure S1. (Continued)

I H1N1S1 (a)

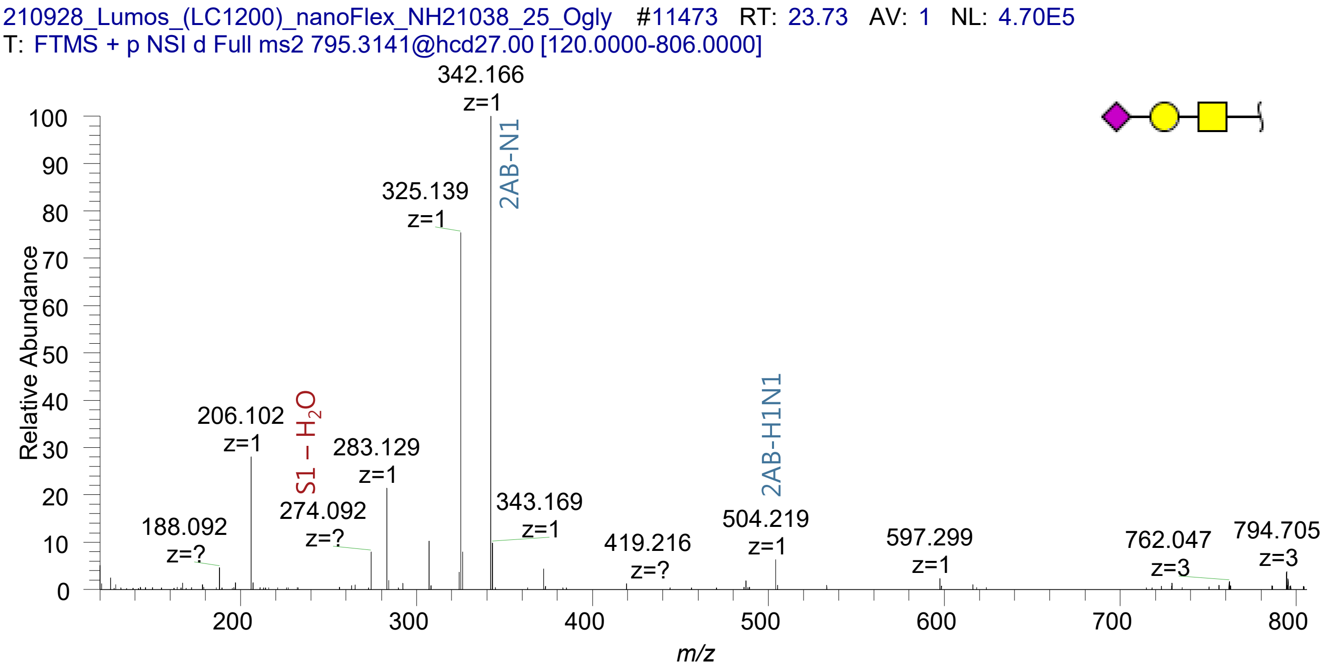

J H1N1S1 (b)

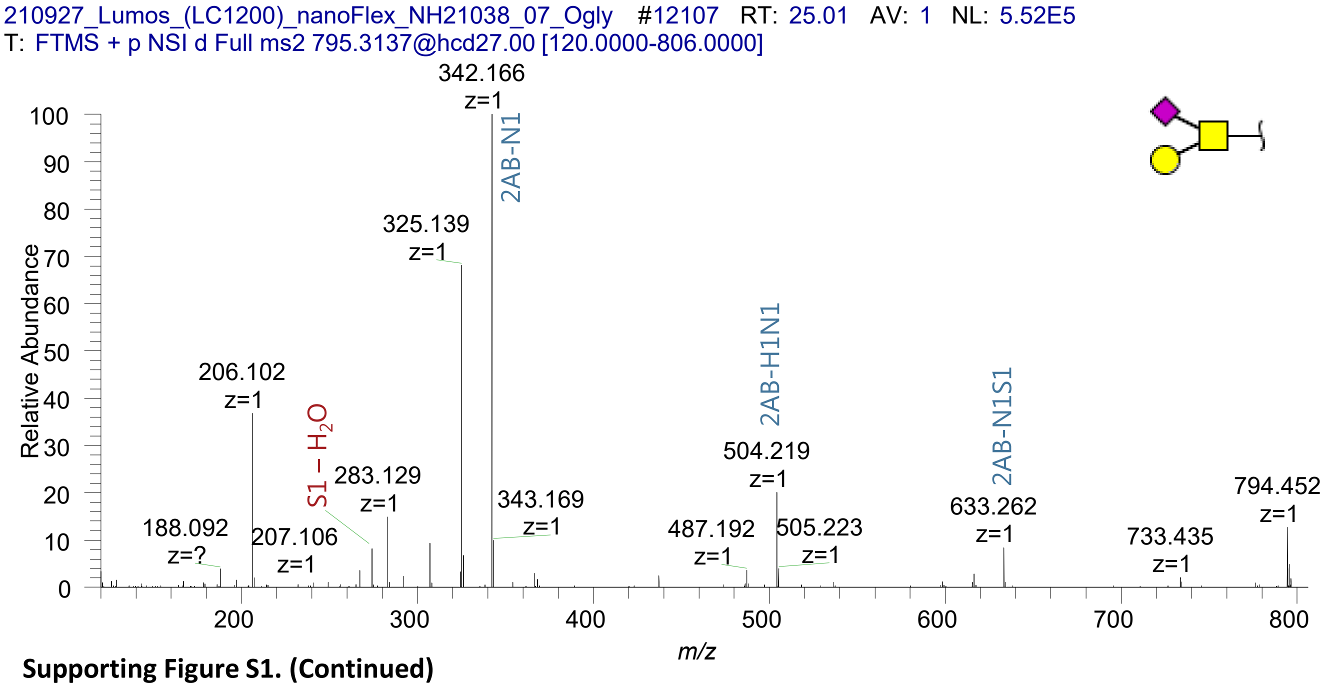

Supporting Figure S1. (Continued)

K H1N2F1

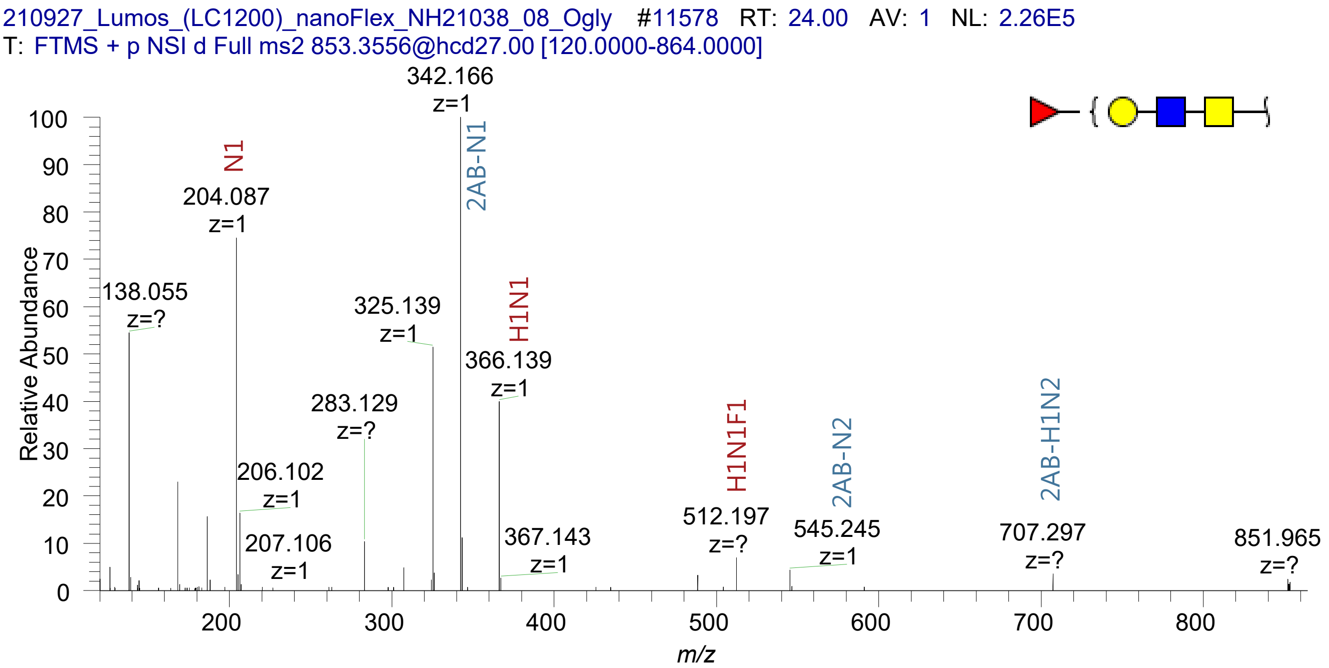

L H2N2 (b)

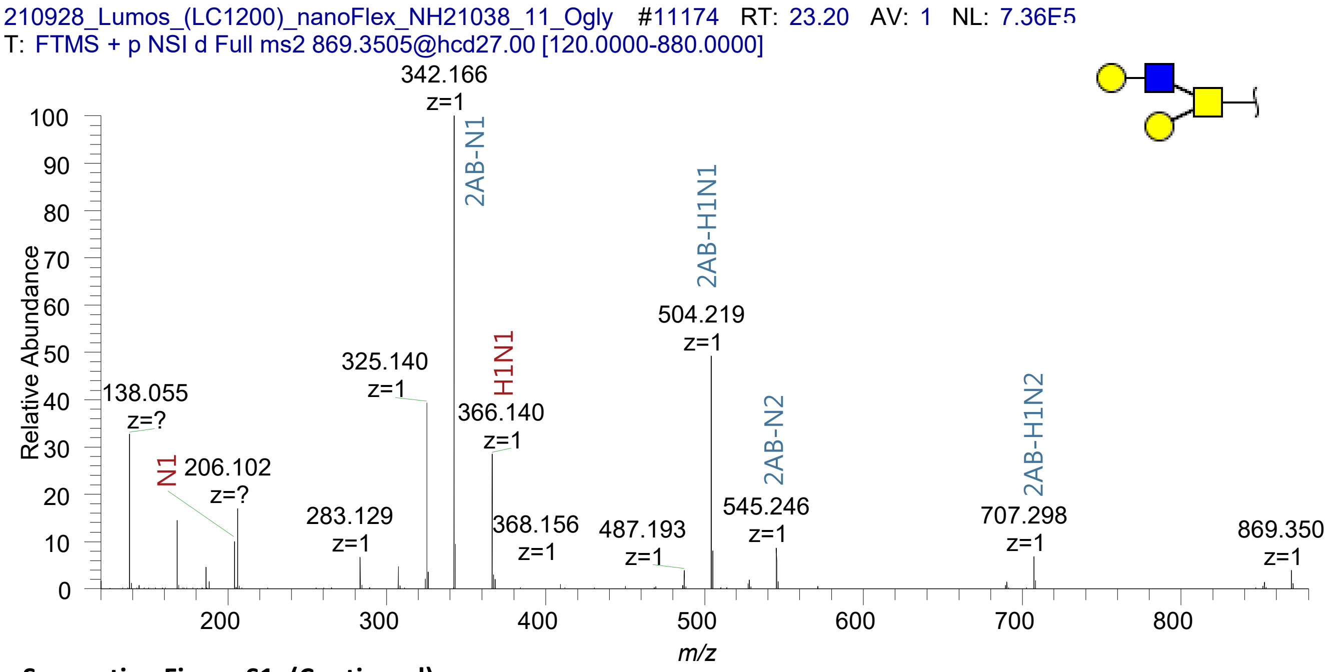

Supporting Figure S1. (Continued)

M H1N2S1 (a)

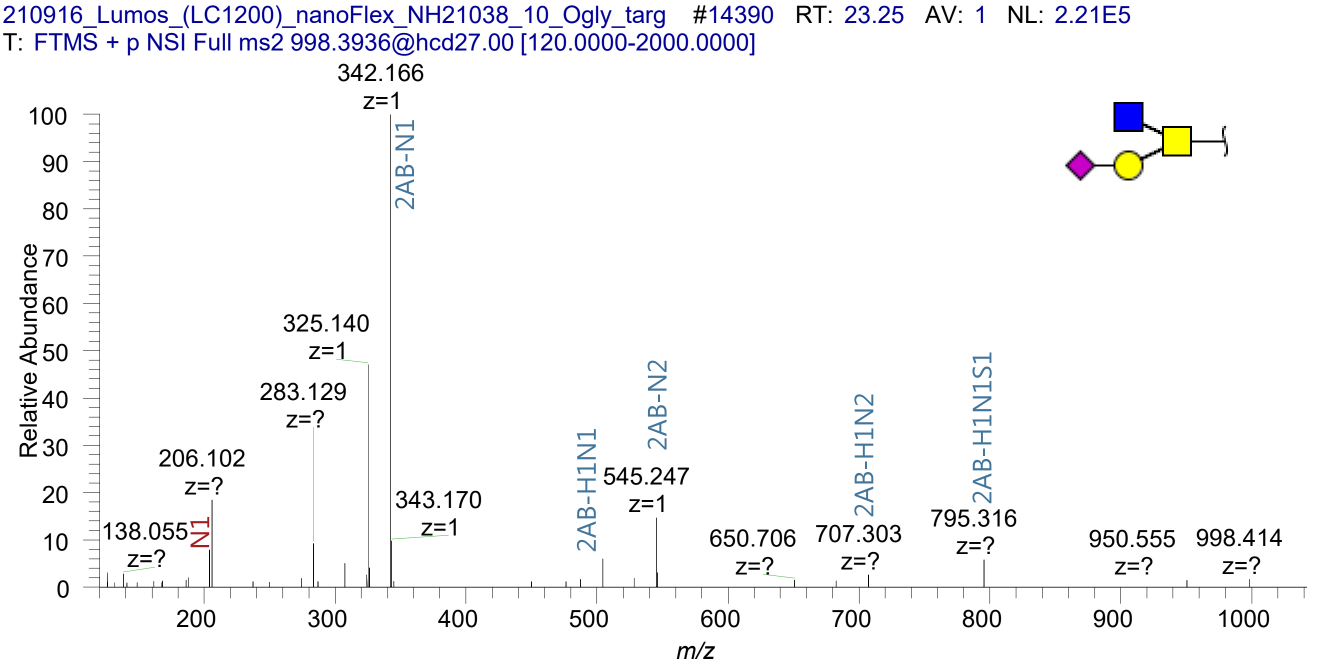

N H1N2S1 (b)

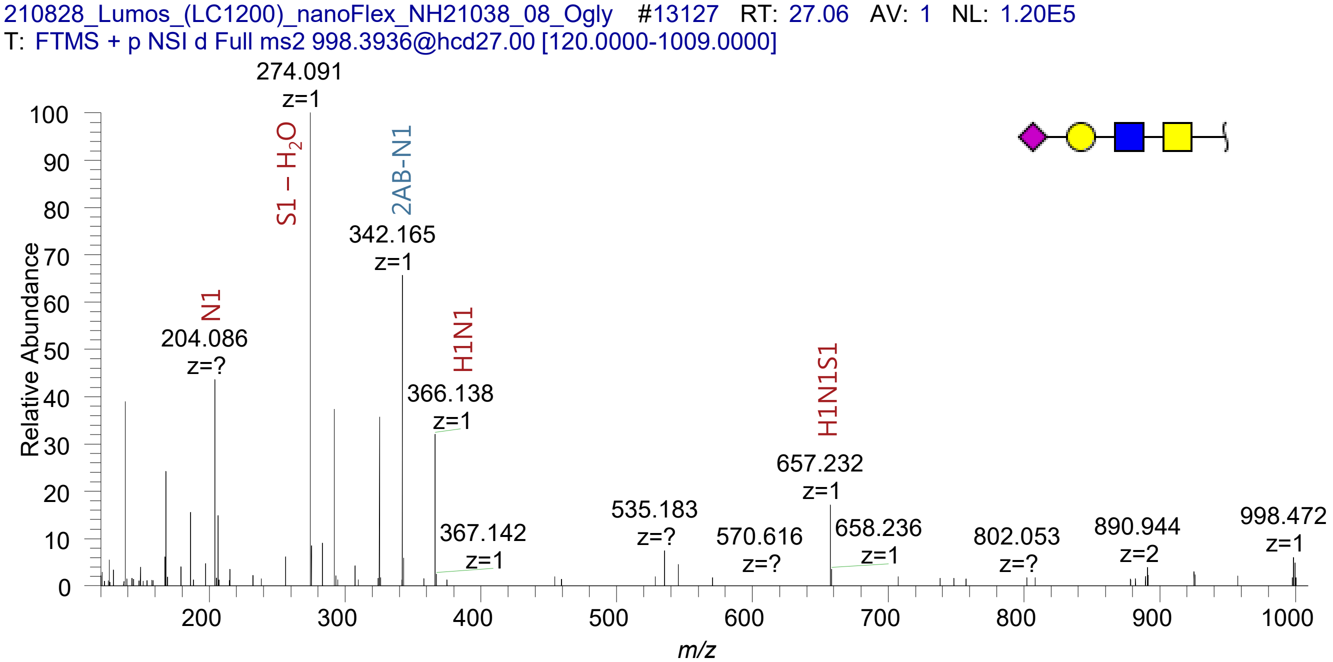

Supporting Figure S1. (Continued)

O H1N1S2

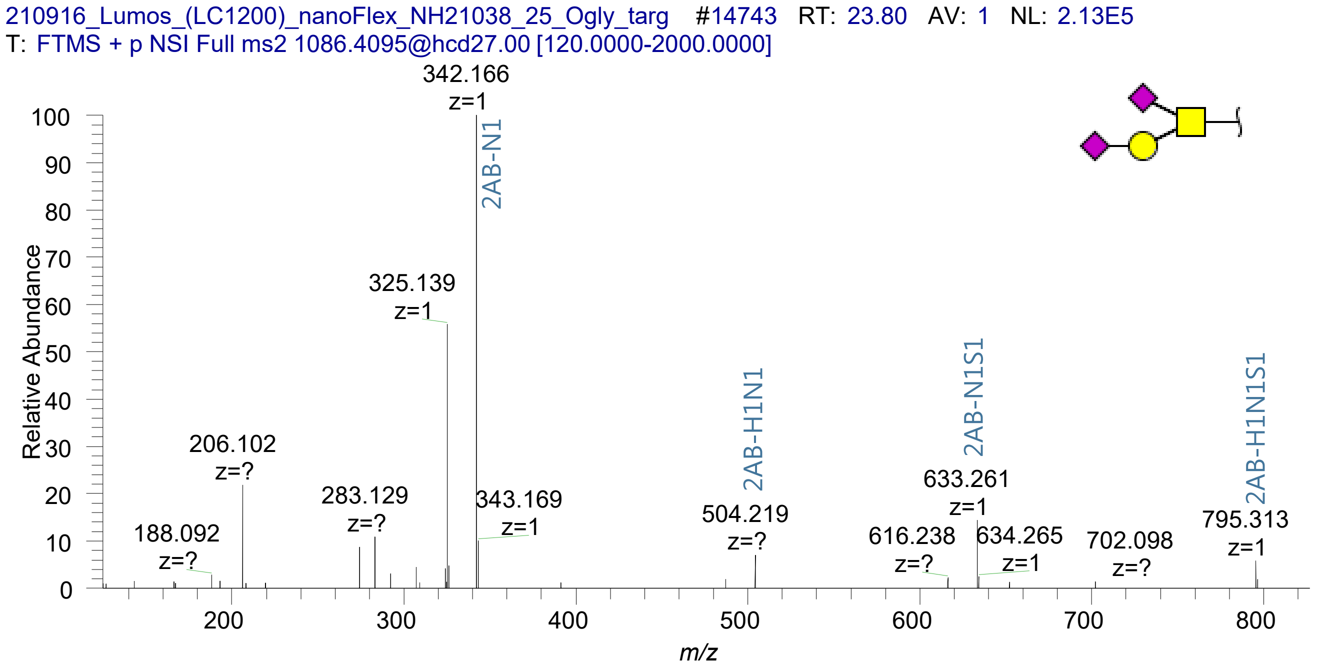

P H2N2S2 (a)

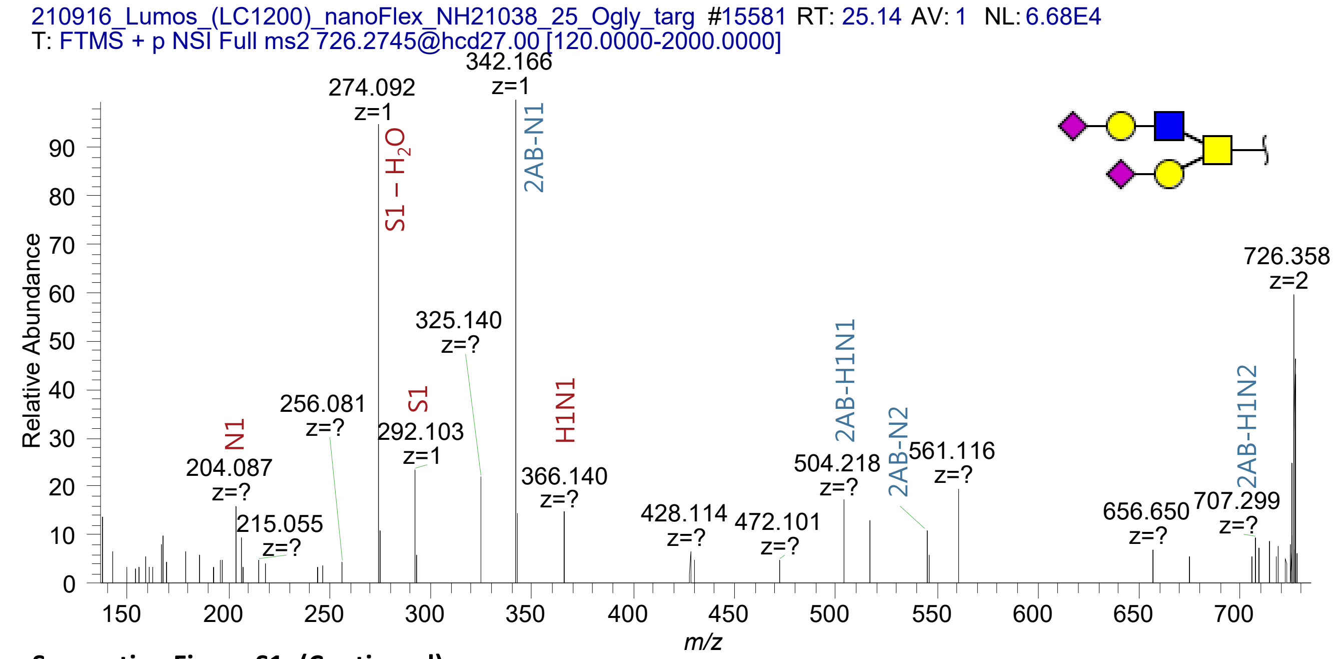

Supporting Figure S1. (Continued)

A N1 (a)

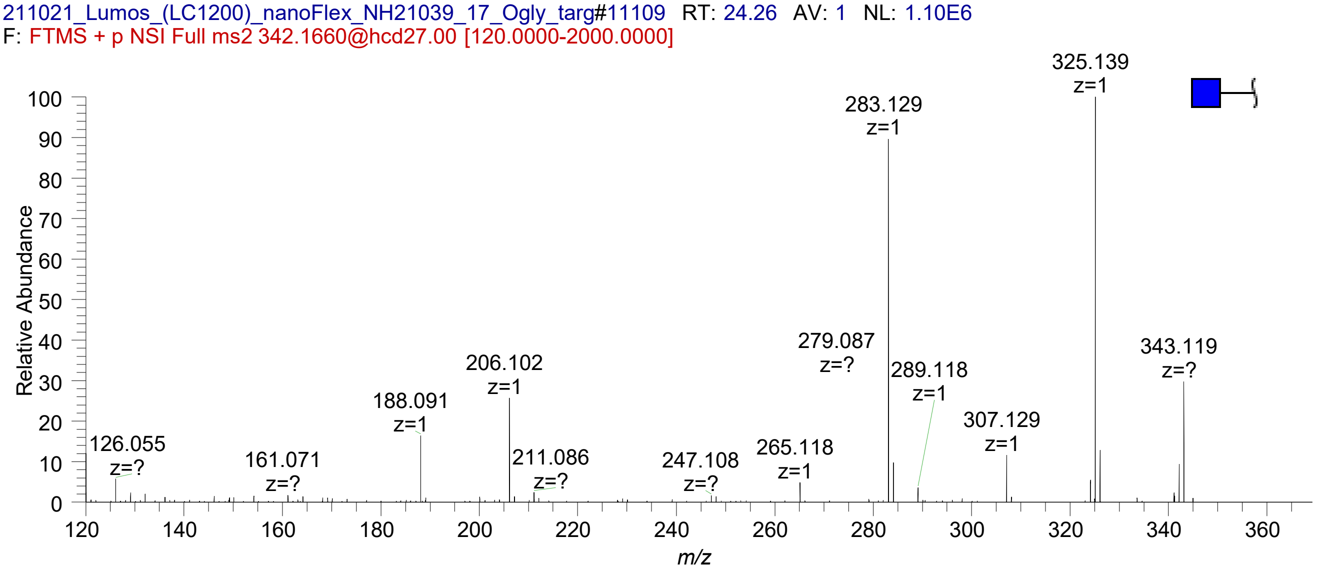

B N1 (b)

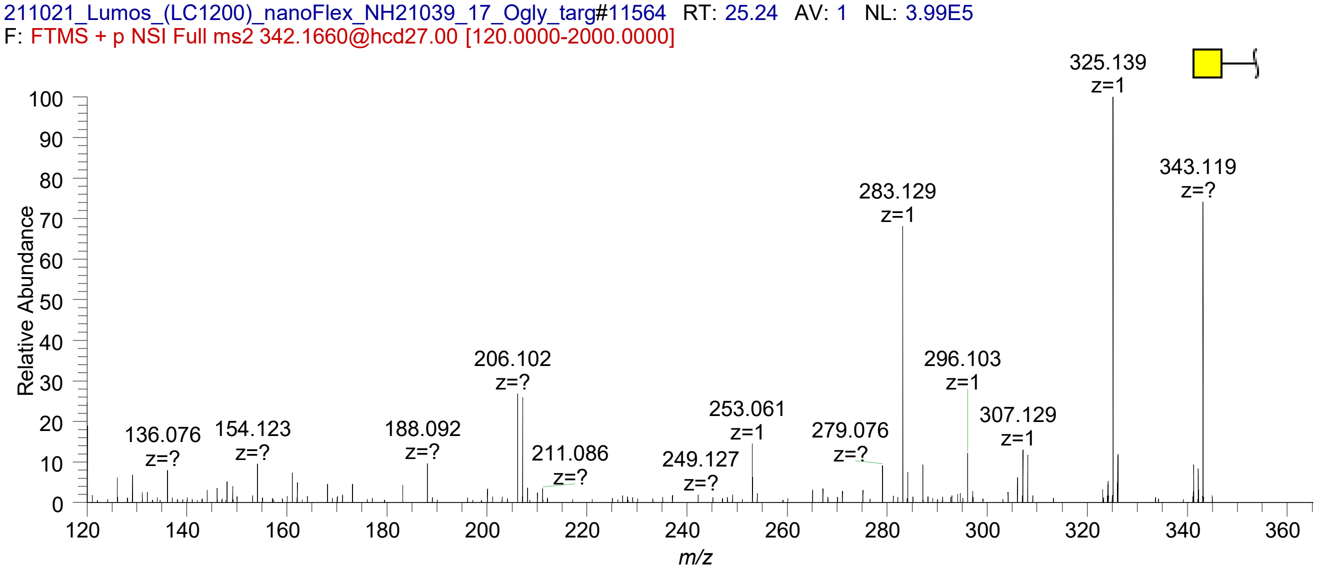

Supporting Figure S2. HCD-MS/MS of keratinocyte *O*-glycans. Y-ions are indicated by compositions in blue, oxonium ions in red. H: Hexose, N: *N*-acetylhexosamine, F: fucose, S: *N*-acetylneuraminic acid, 2-AB: reducing end label. **Supporting Table S1** lists the ions used for annotation of the proposed structures. The depiction of the glycans indicates monosaccharide identity and connectivity, but not linkage. Additional evidence for the proposed depictions can be found in **Supporting Table S3 and S4**.

C H1X1

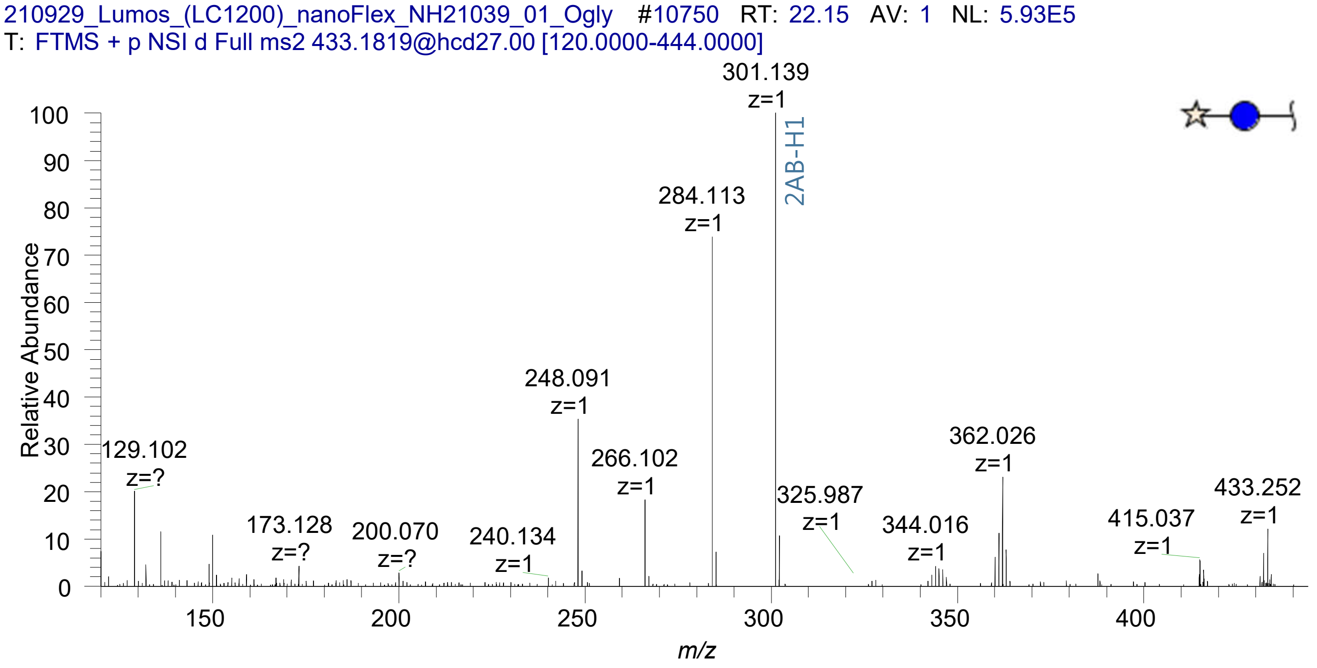

D H1F1

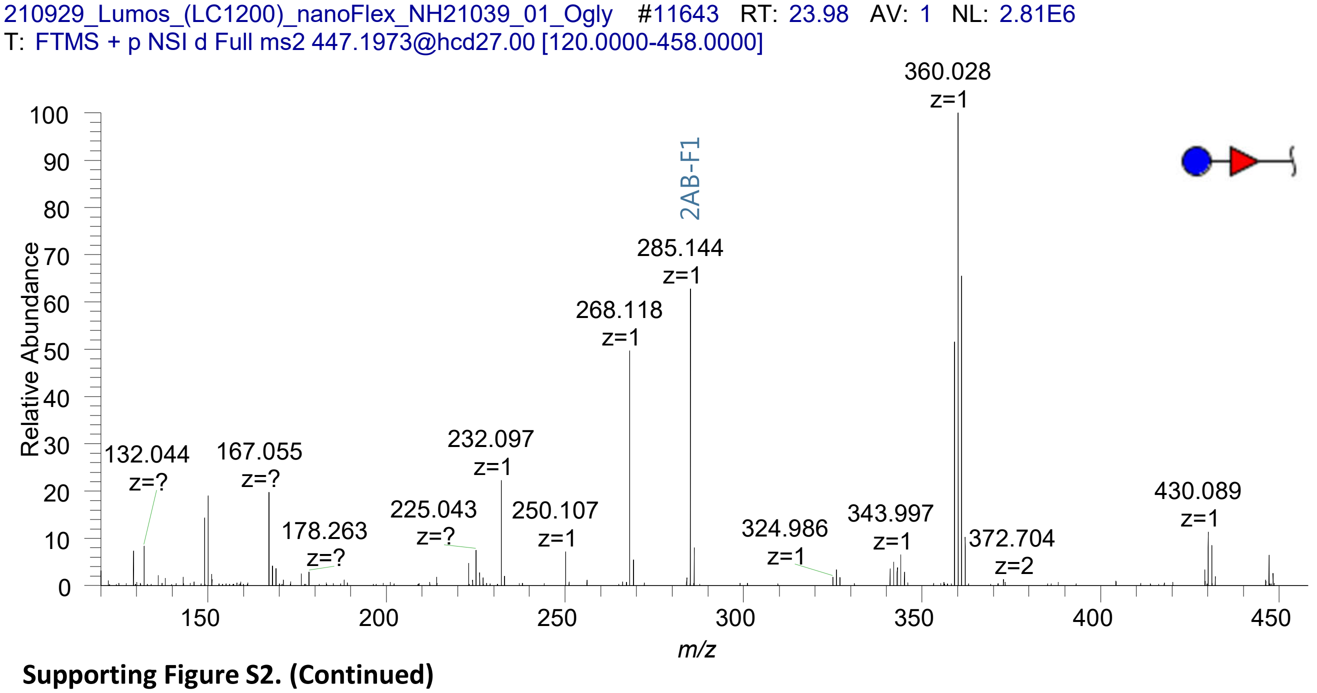

Supporting Figure S2. (Continued)

E H1N1 (a)

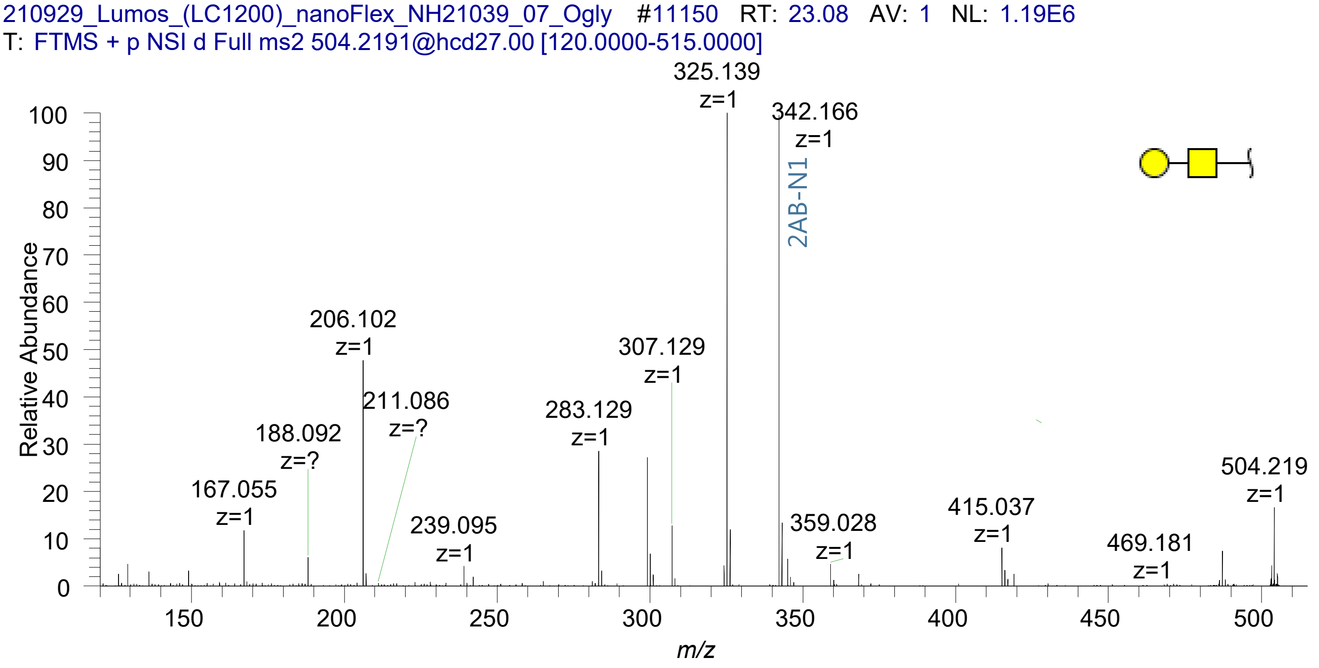

F H1N1 (b)

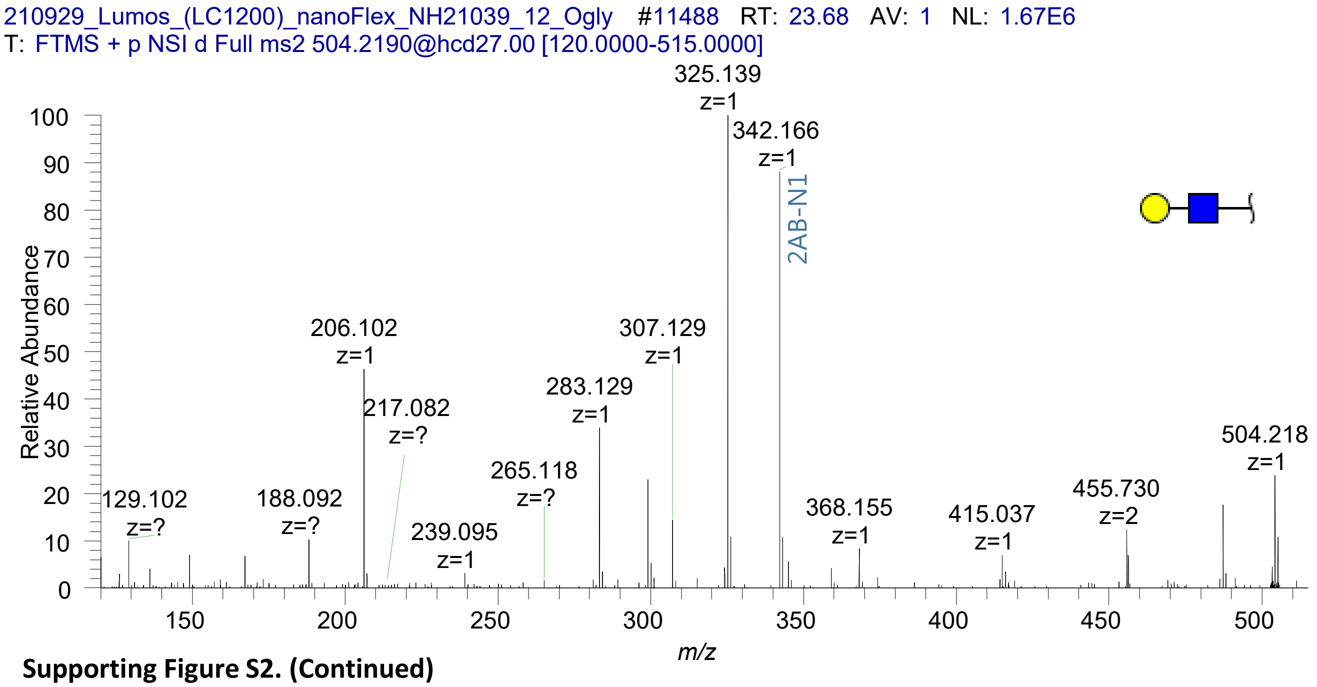

Supporting Figure S2. (Continued)

G N2

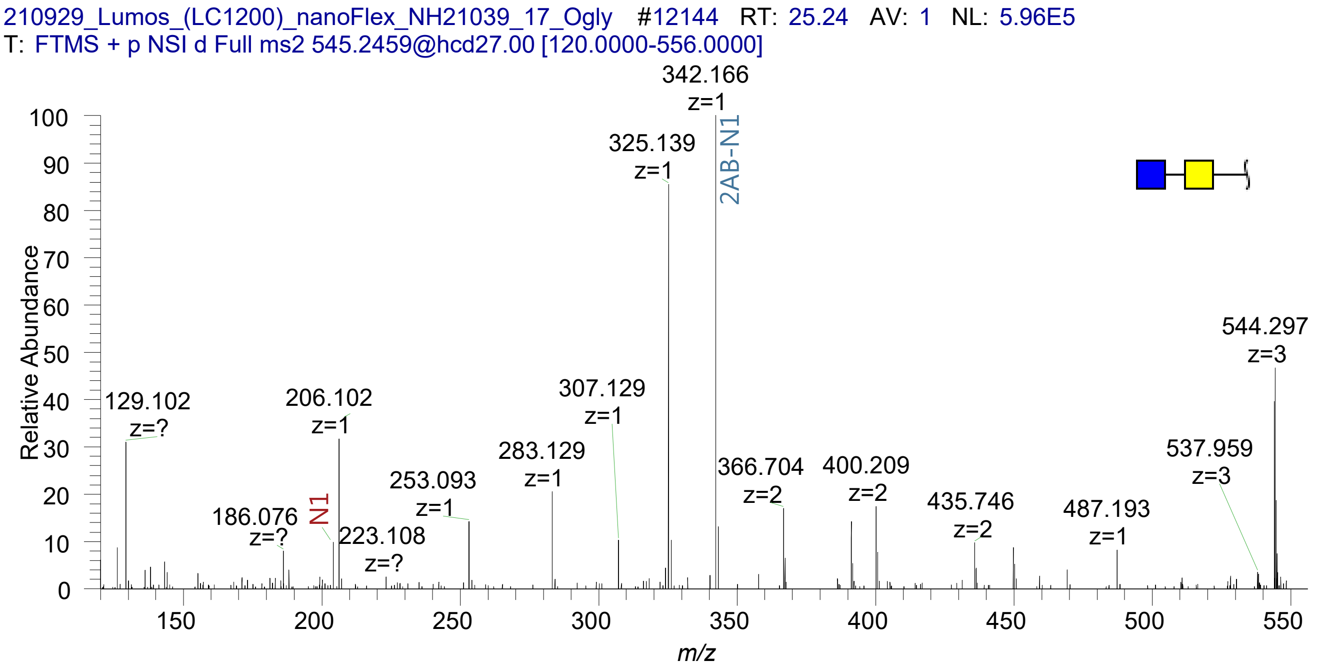

H H1X2

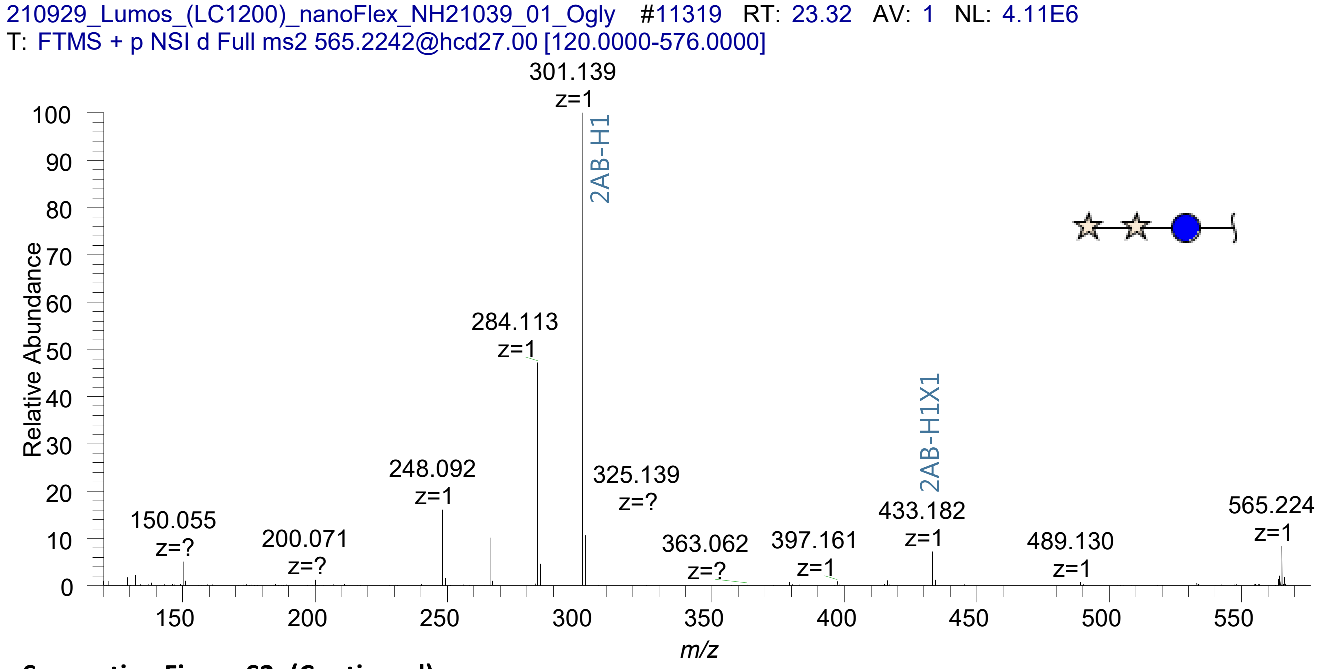

Supporting Figure S2. (Continued)

I H1S1

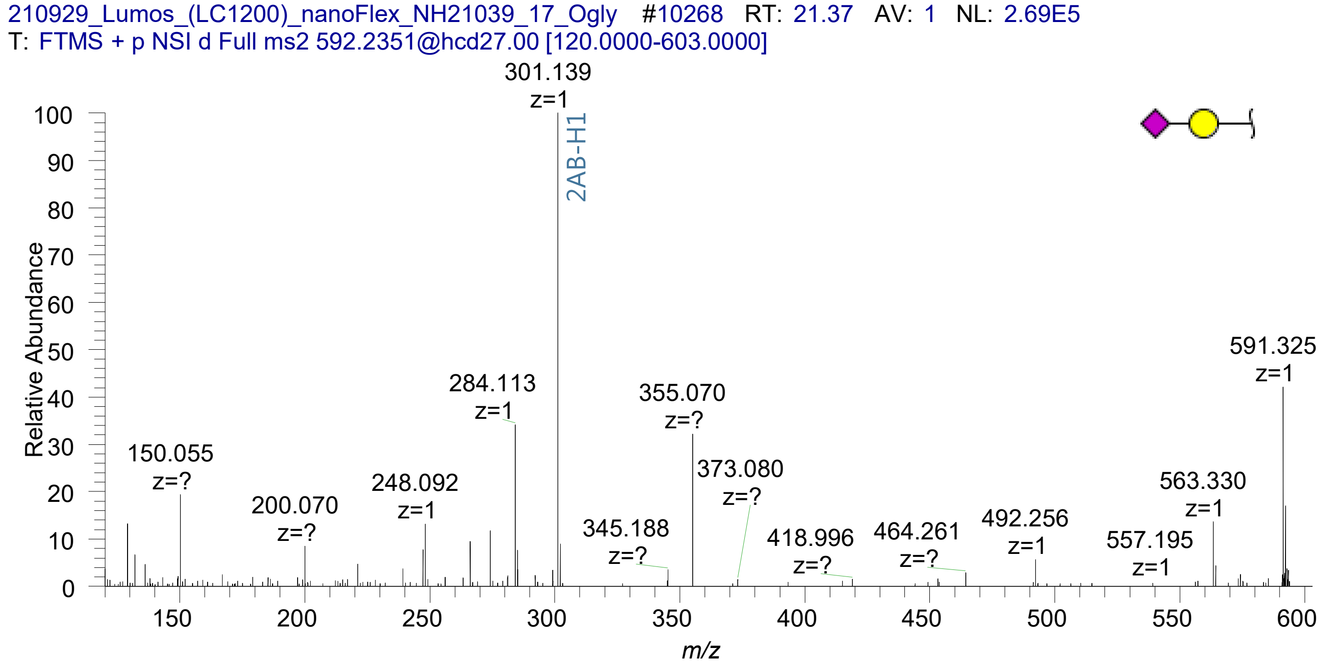

J H2F1

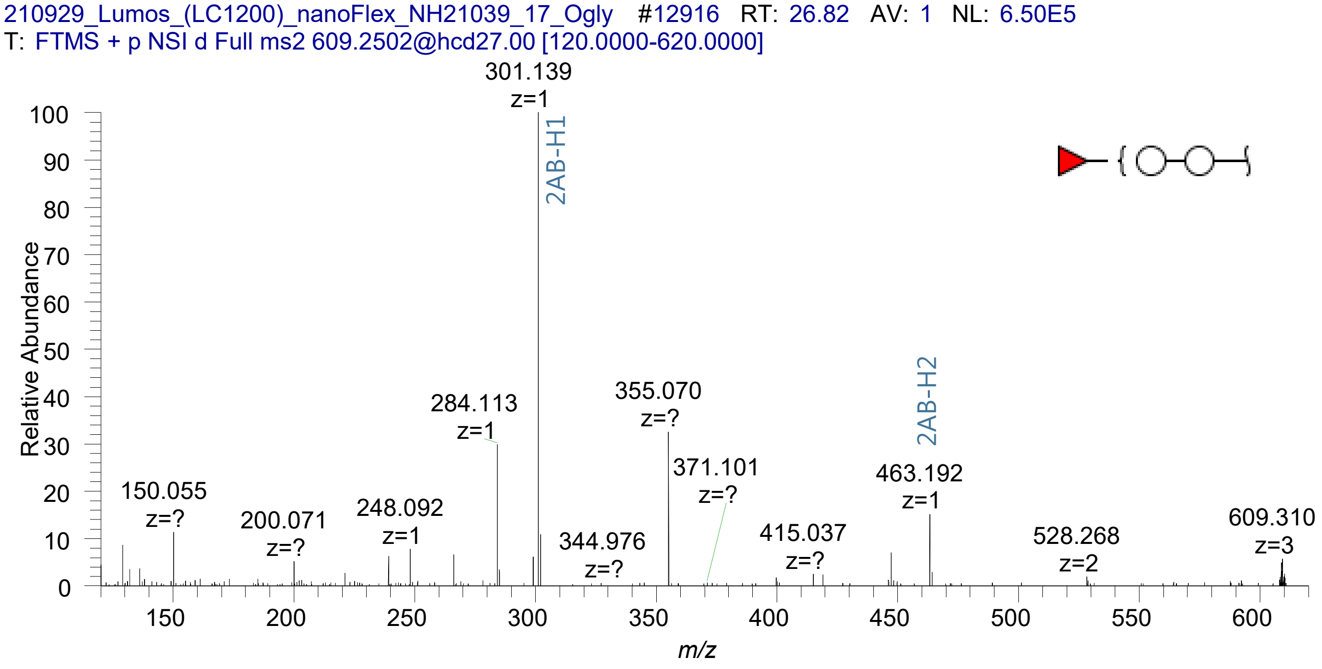

Supporting Figure S2. (Continued)

K N1S1

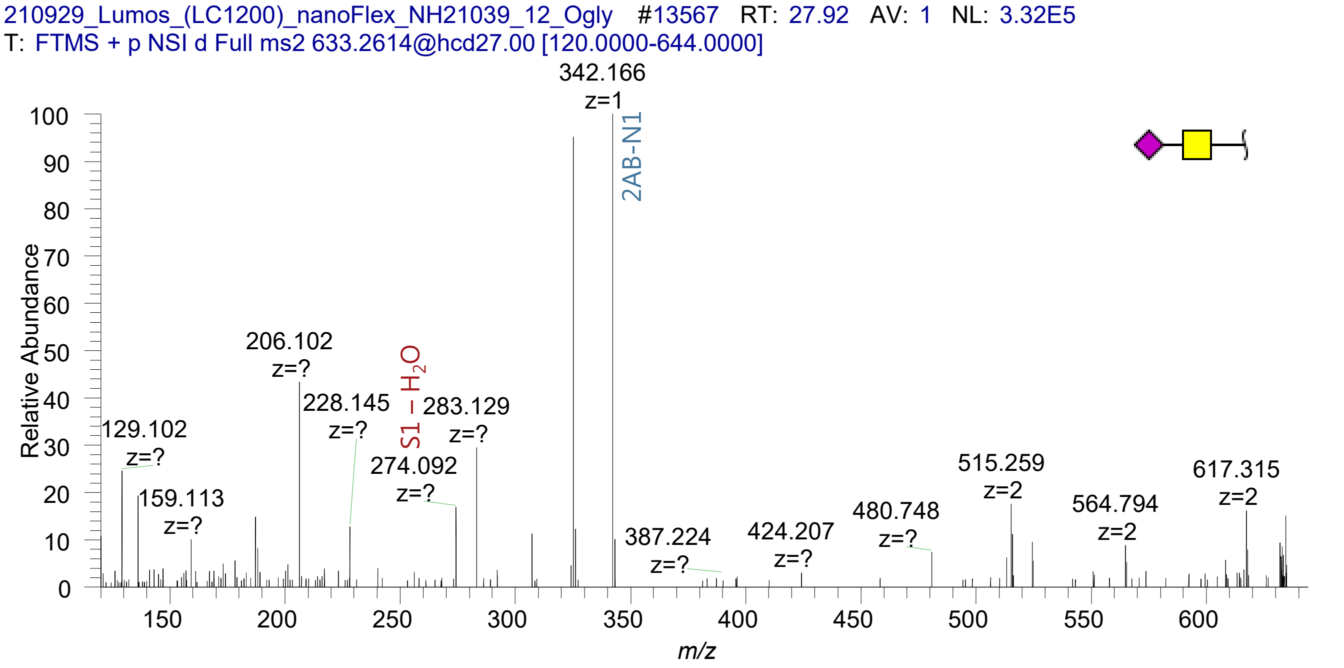

Supporting Figure S2. (Continued)

L H1N1F1 (a)

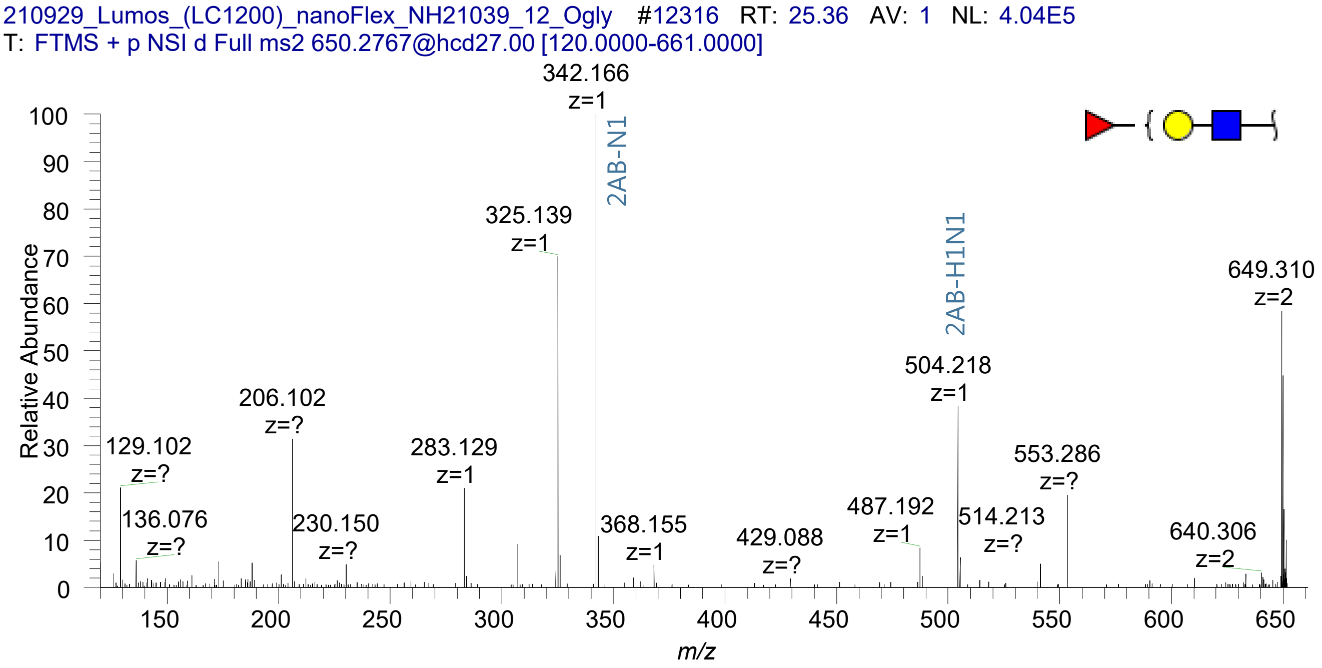

M H1N1F1 (b)

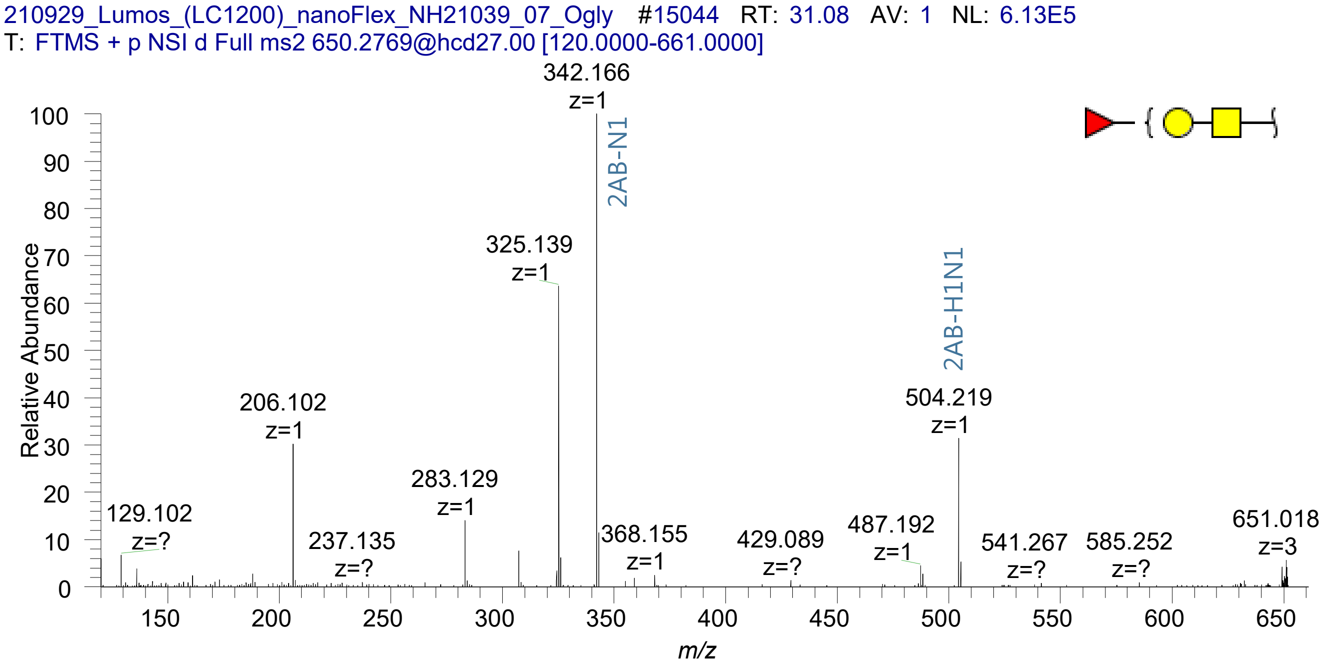

Supporting Figure S2. (Continued)

N H2N1 (a)

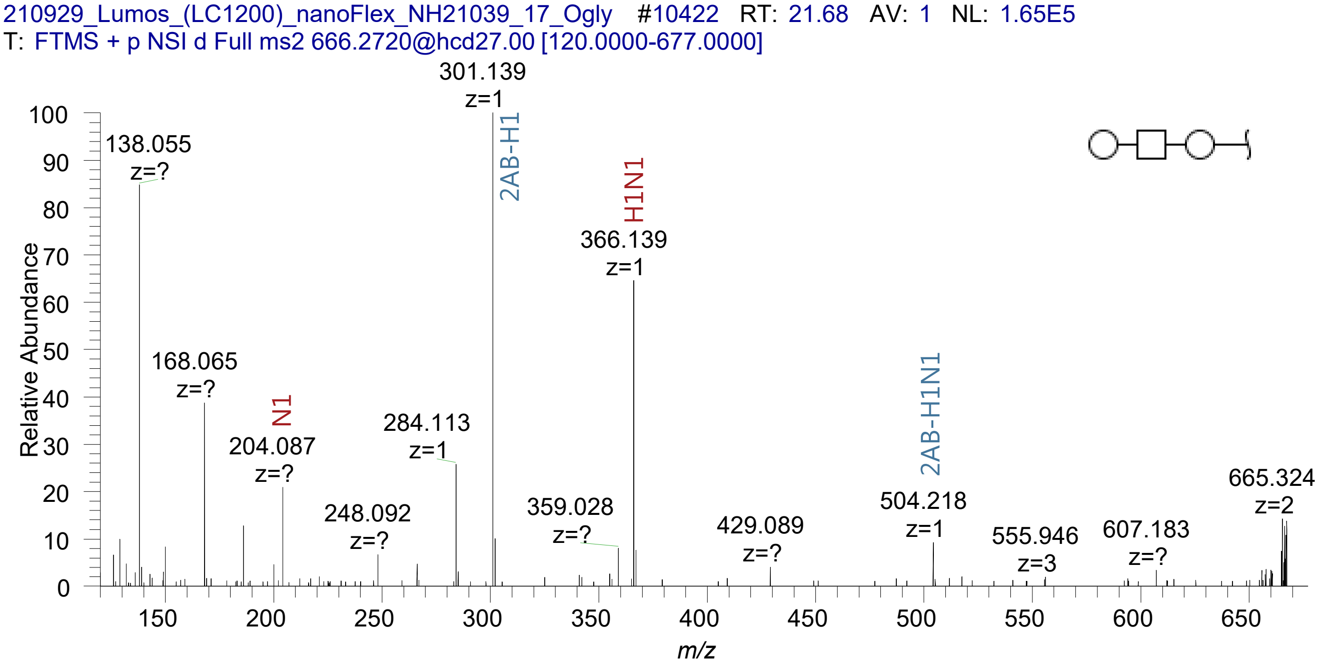

O H2N1 (c)

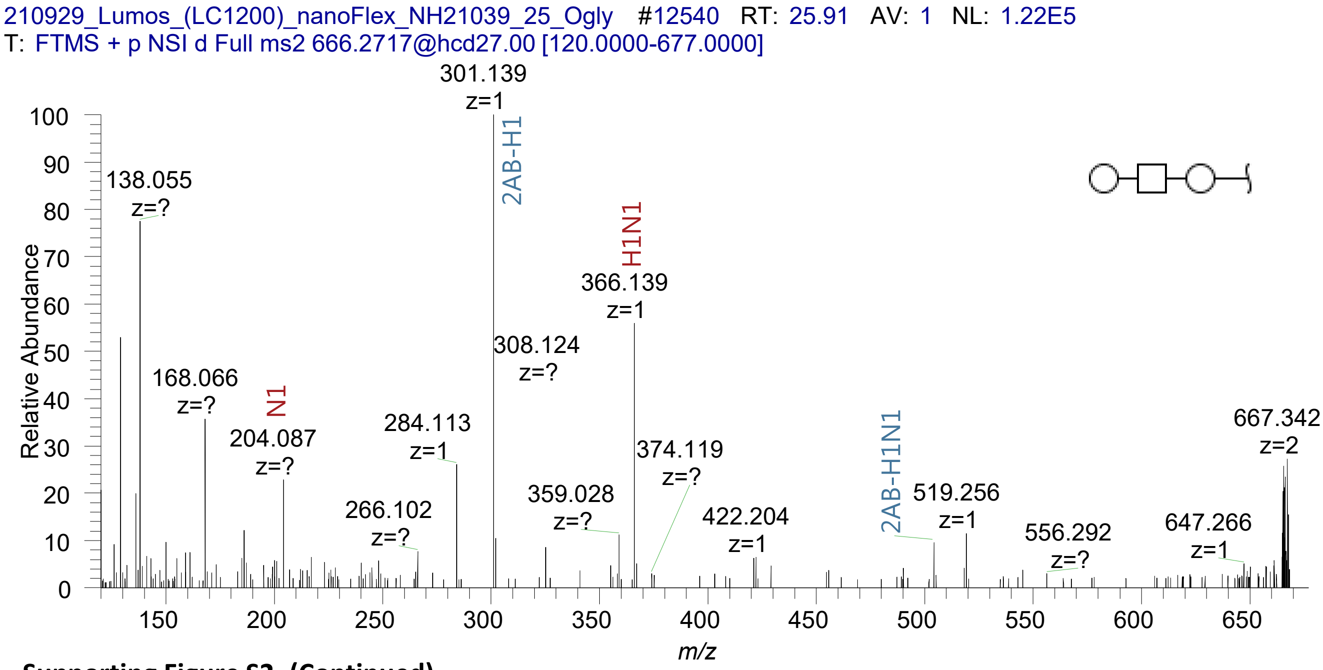

Supporting Figure S2. (Continued)

P H1N2 (a)

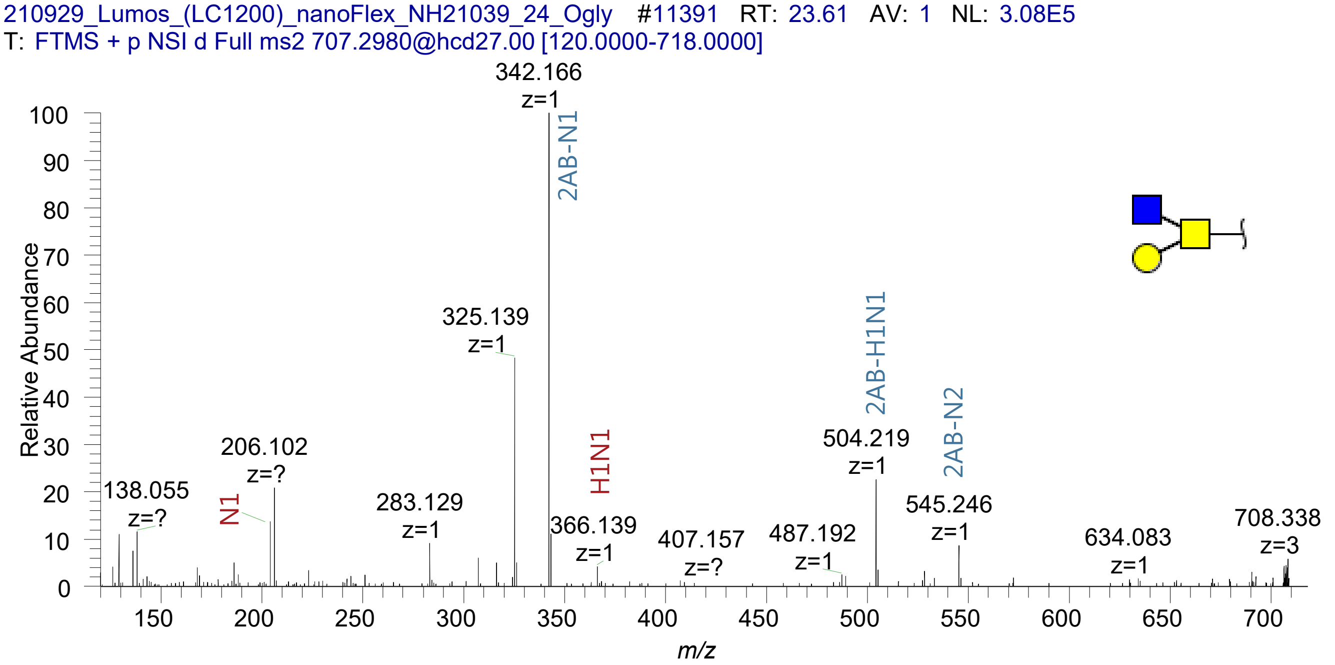

Q H1N2 (c)

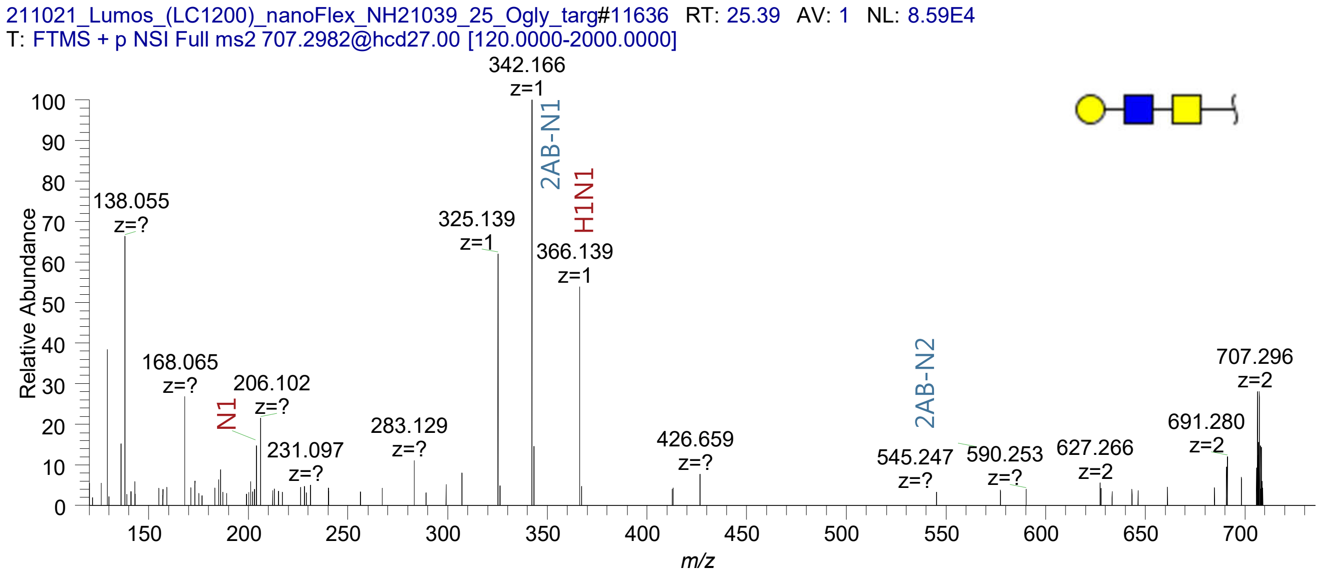

Supporting Figure S2. (Continued)

R H1N1S1 (a)

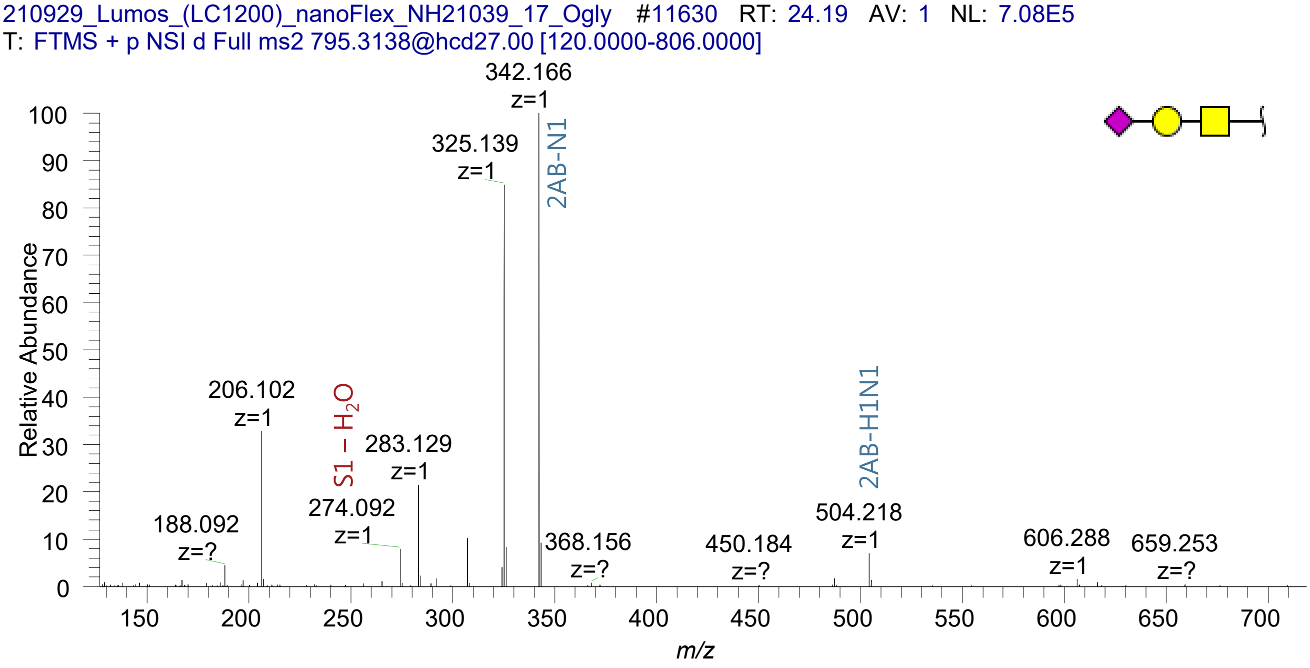

S H1N1S1 (b)

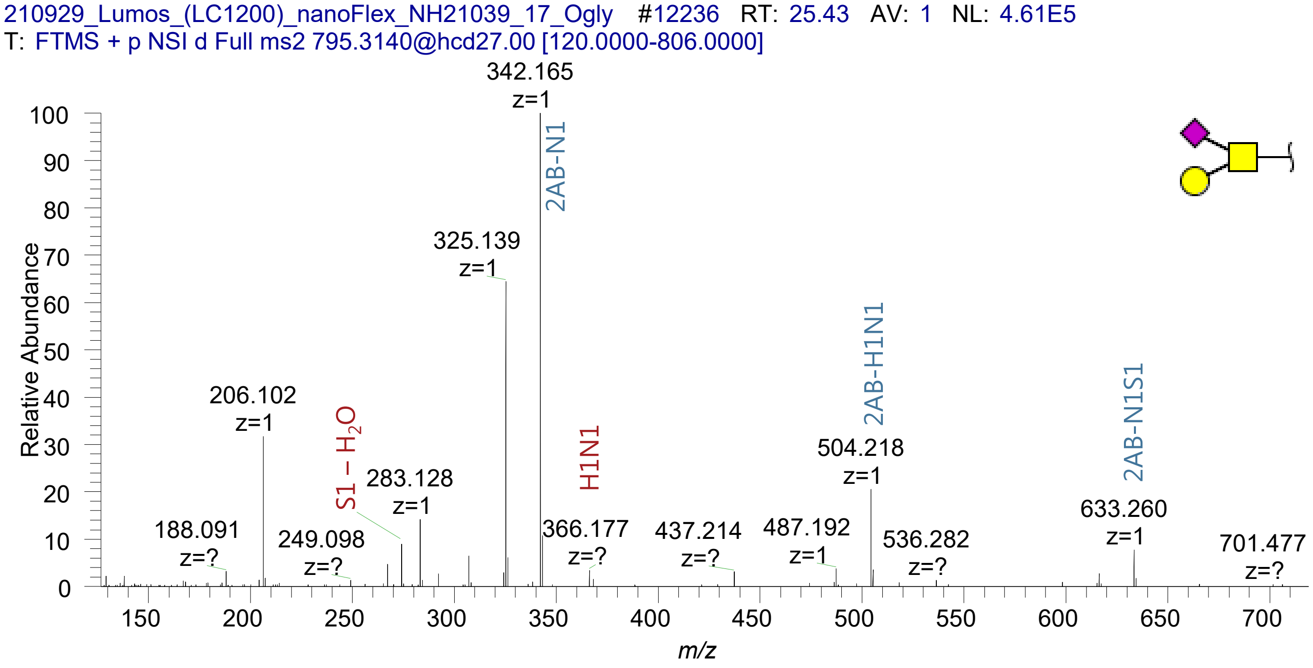

Supporting Figure S2. (Continued)

T H1N1S1 (c)

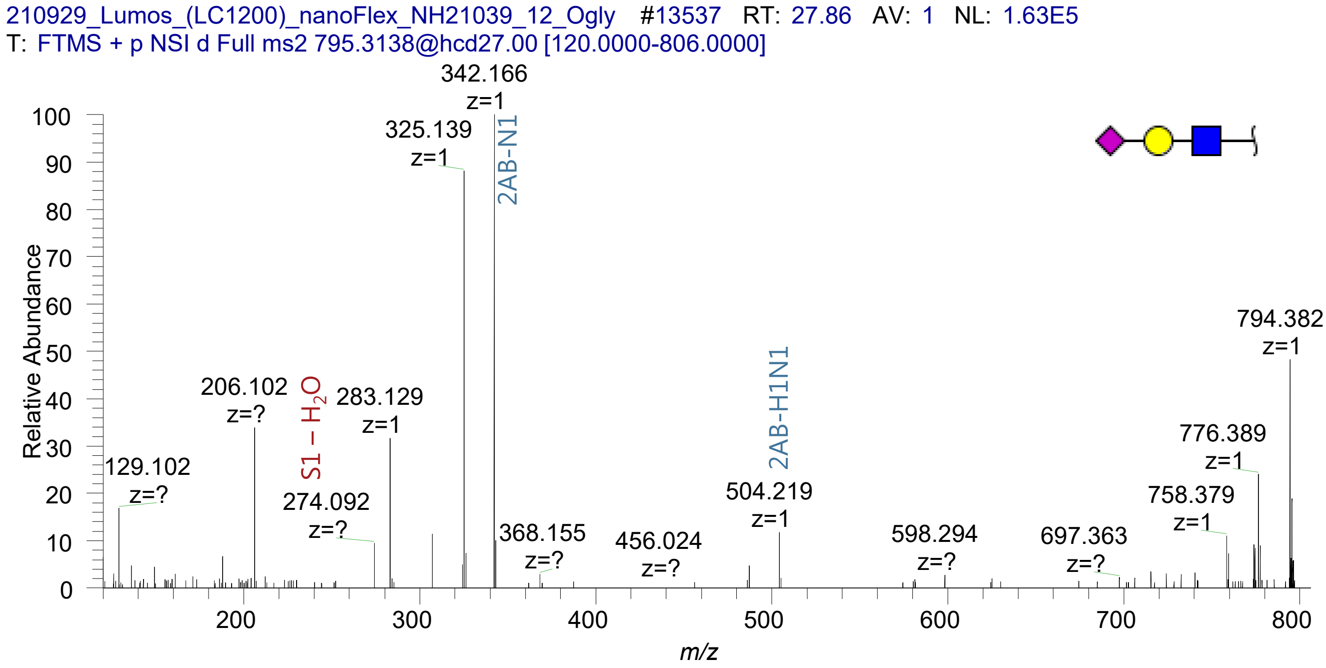

U H2N1F1

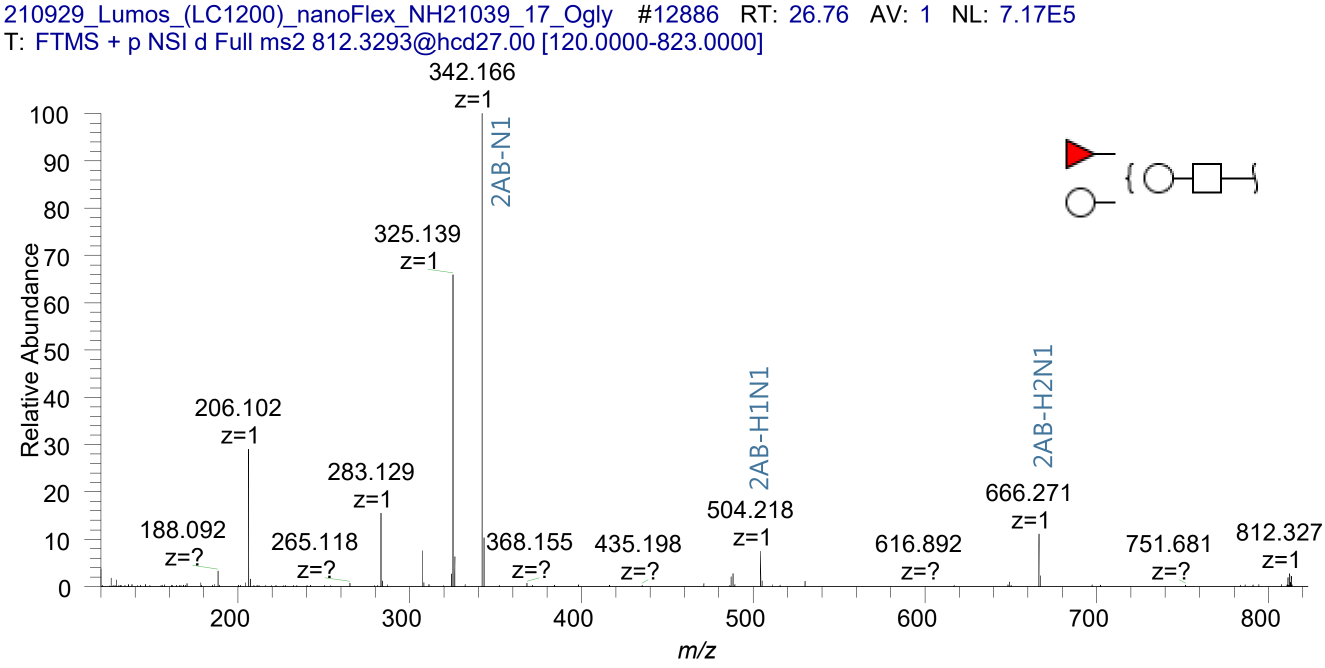

Supporting Figure S2. (Continued)

V N2S1

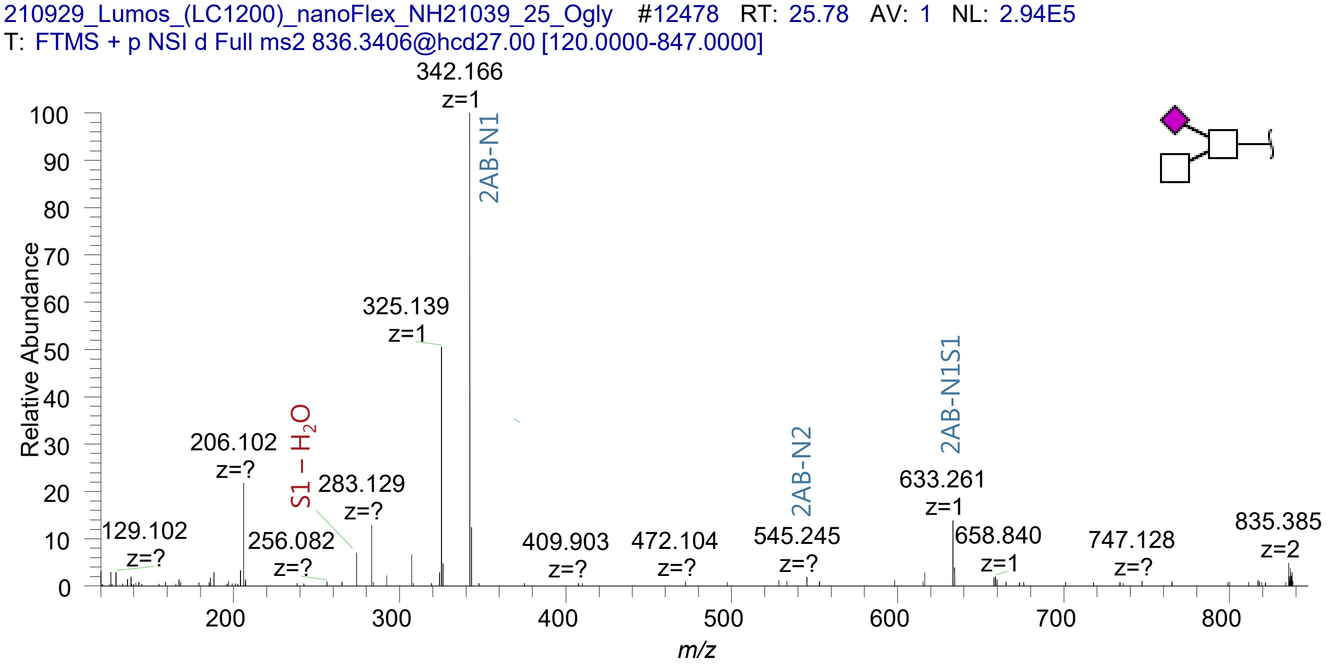

Supporting Figure S2. (Continued)

W H2N2 (a)

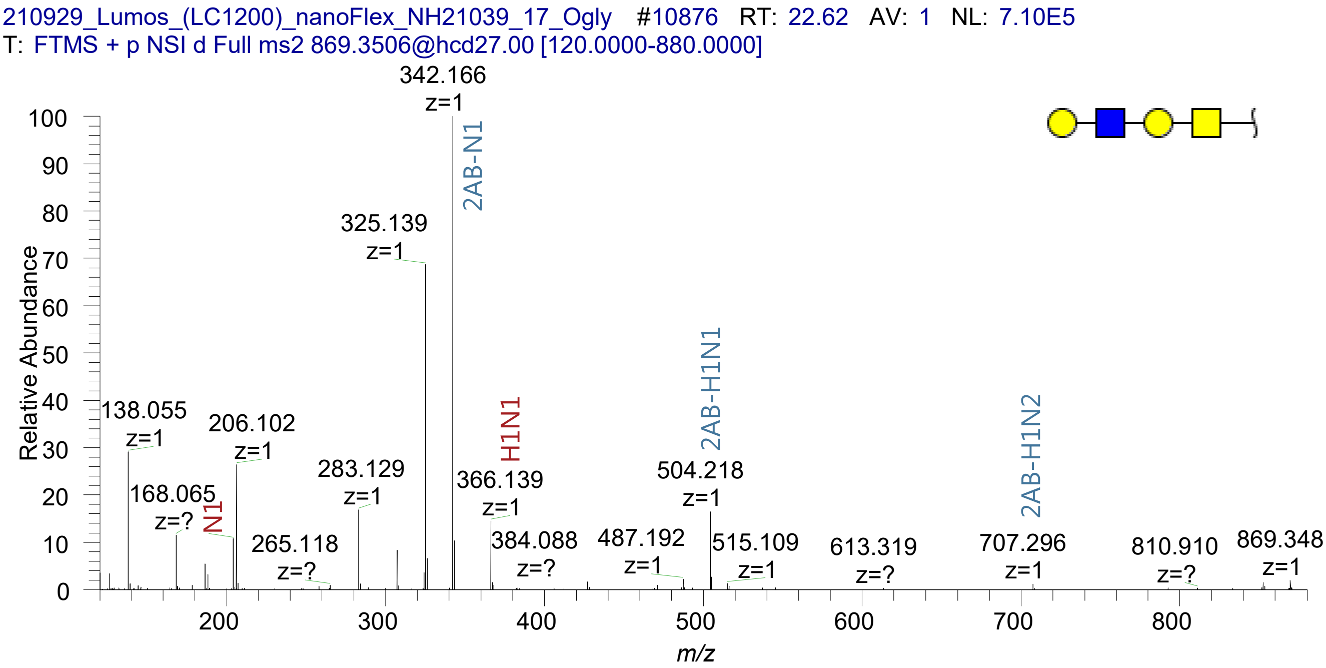

X H2N2 (b)

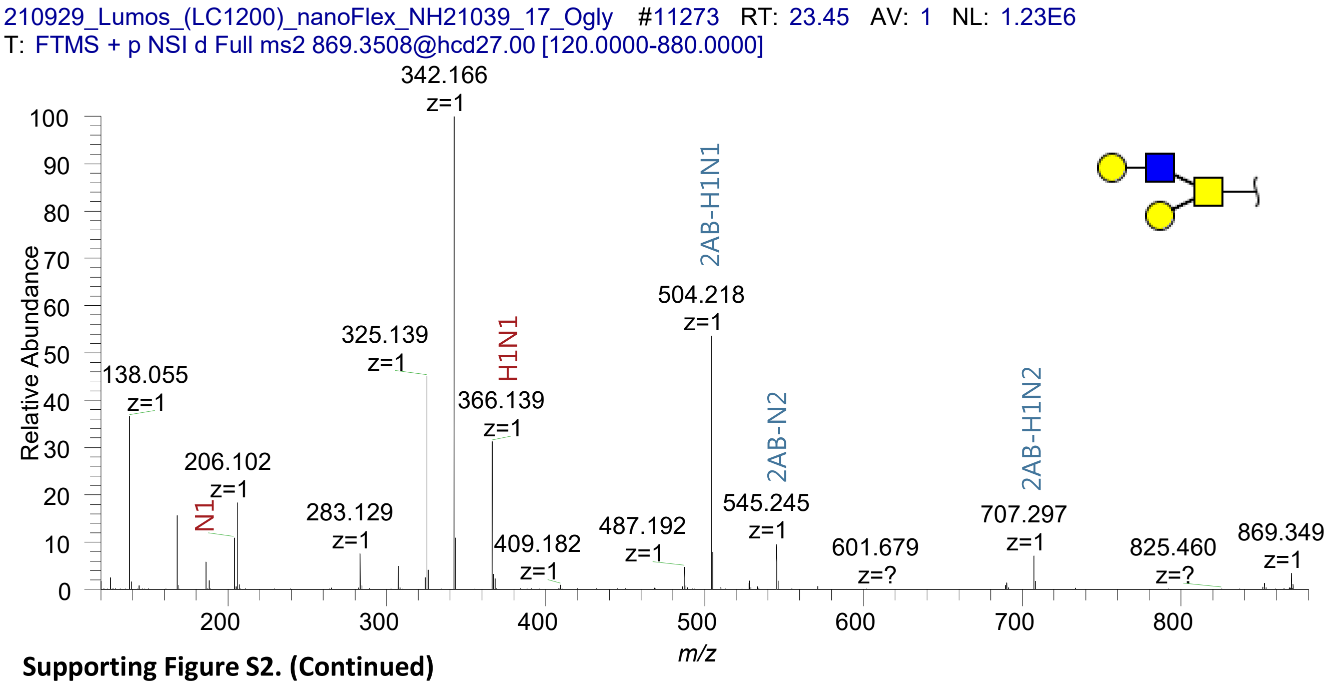

Supporting Figure S2. (Continued)

Y H2N1S1

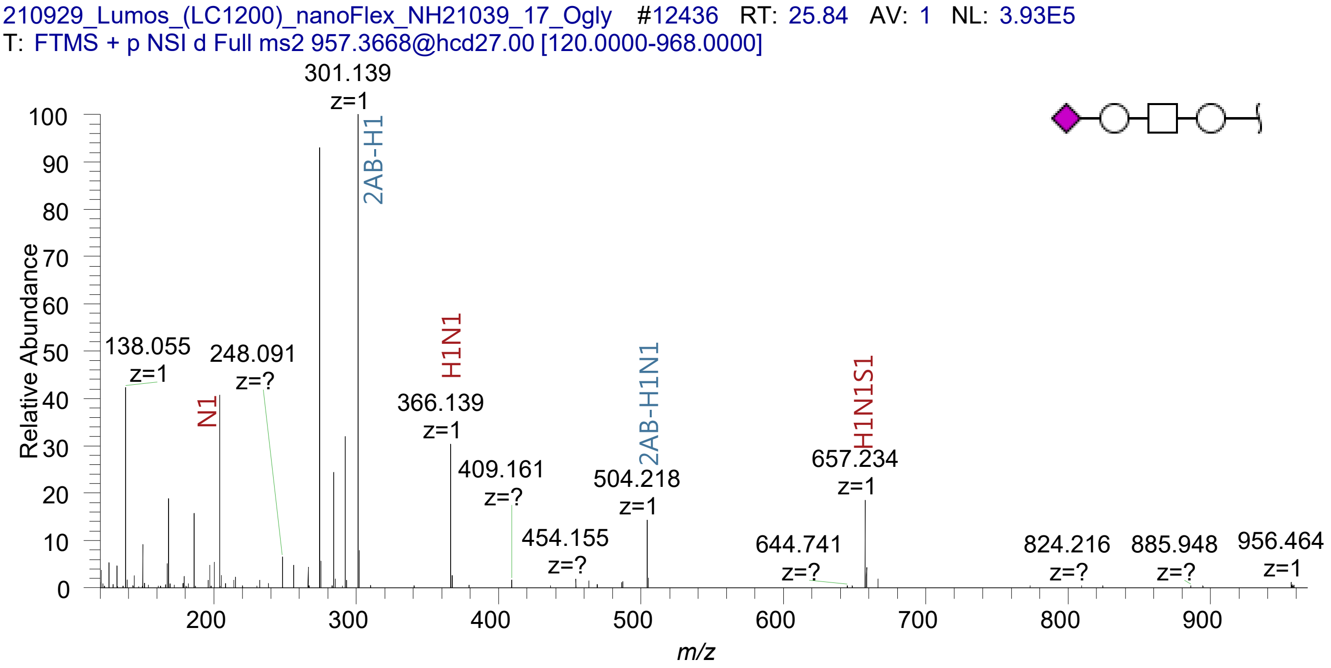

Z H2N2F1 (a)

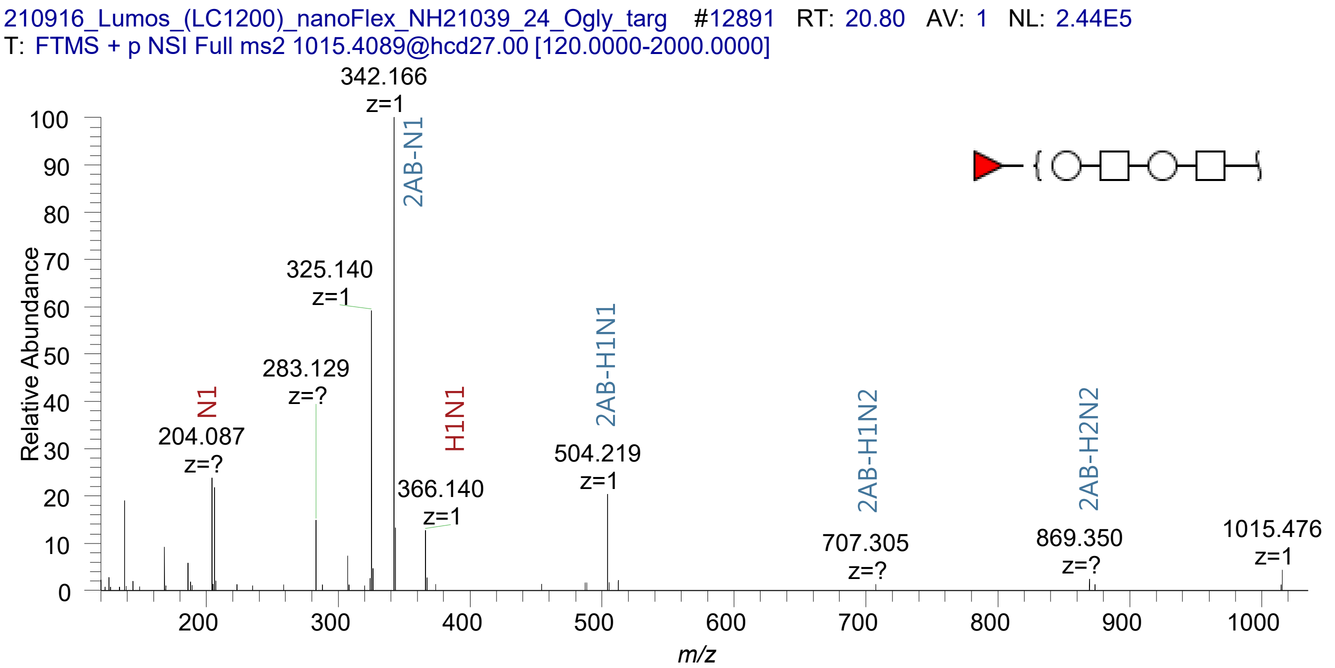

Supporting Figure S2. (Continued)

AA H2N2F1 (b)

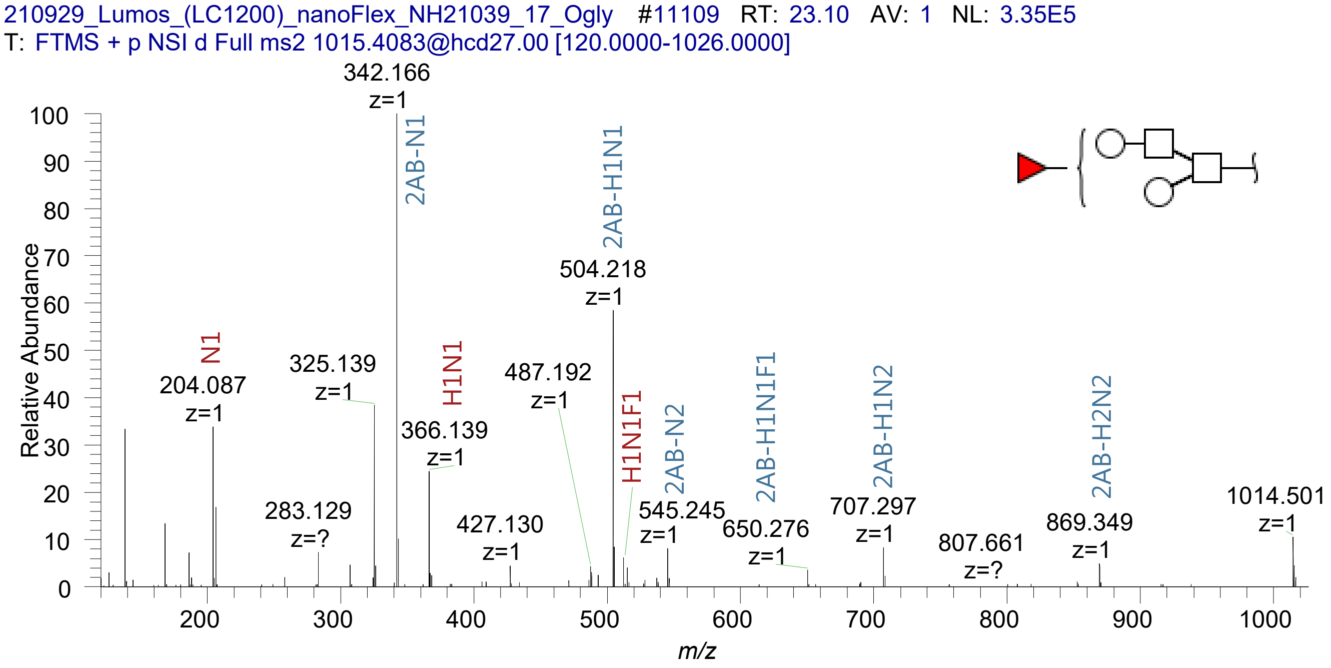

AB H2N2F1 (c)

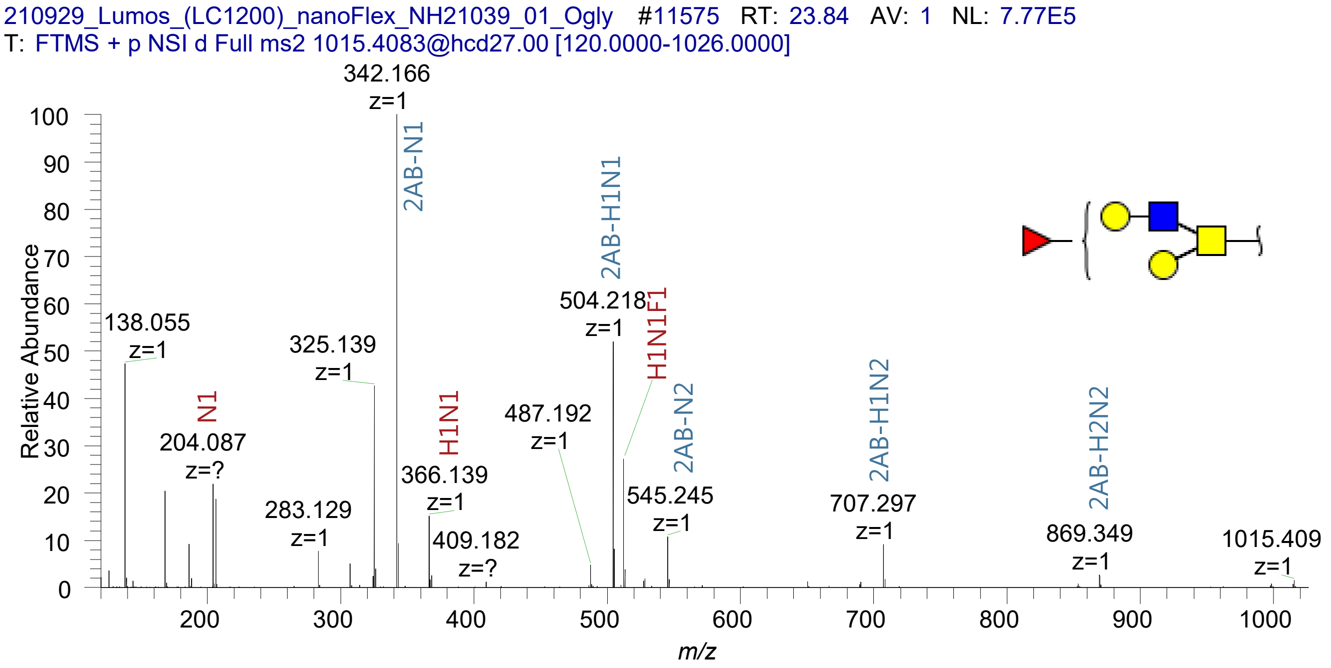

Supporting Figure S2. (Continued)

AC H1N1S2

210929_Lumos_(LC1200)_nanoFlex_NH21039_13_Ogly #11824 RT: 24.50 AV: 1 NL: 1.17E6
T: FTMS + p NSI d Full ms2 1086.4084@hcd27.00 [120.0000-1097.0000]

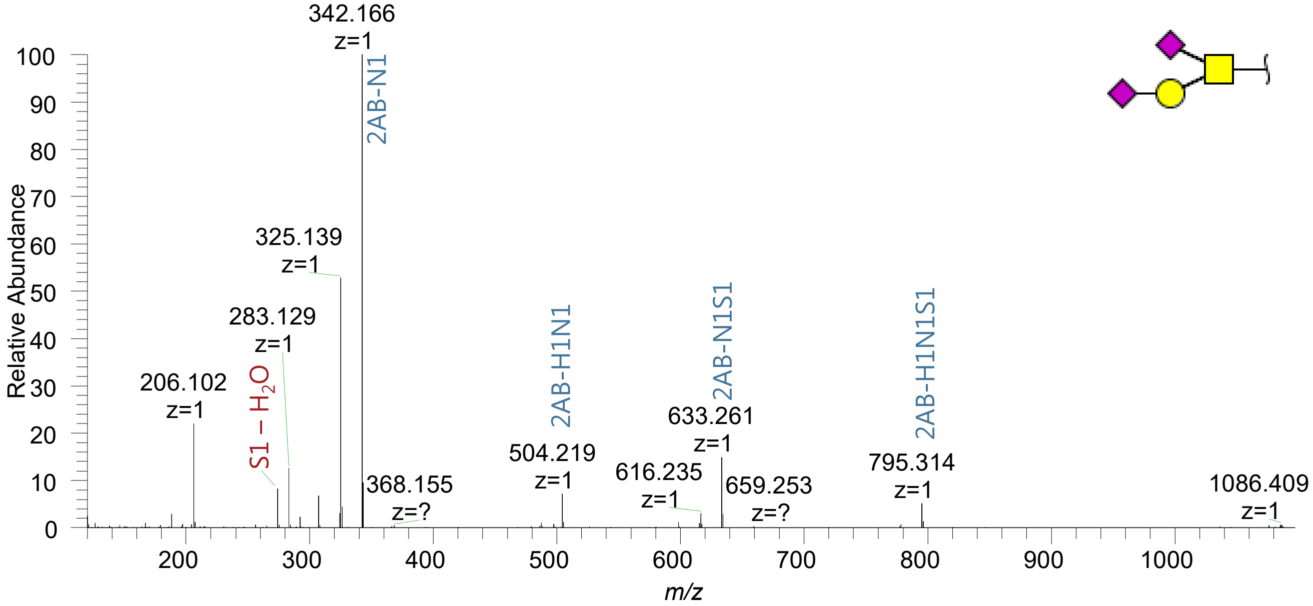

AD H2N2S1 (a)

210929_Lumos_(LC1200)_nanoFlex_NH21039_17_Ogly #11462 RT: 23.84 AV: 1 NL: 1.31E5
T: FTMS + p NSI d Full ms2 580.7272@hcd27.00 [120.0000-1172.0000]

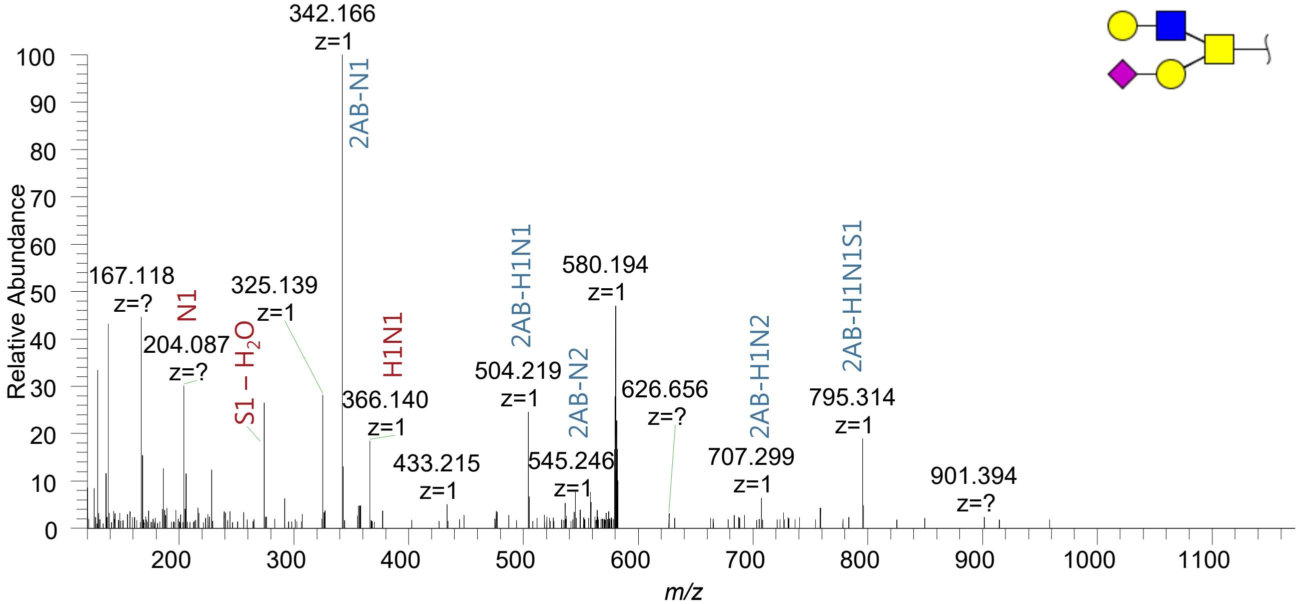

Supporting Figure S2. (Continued)

AE H2N2S1 (b)

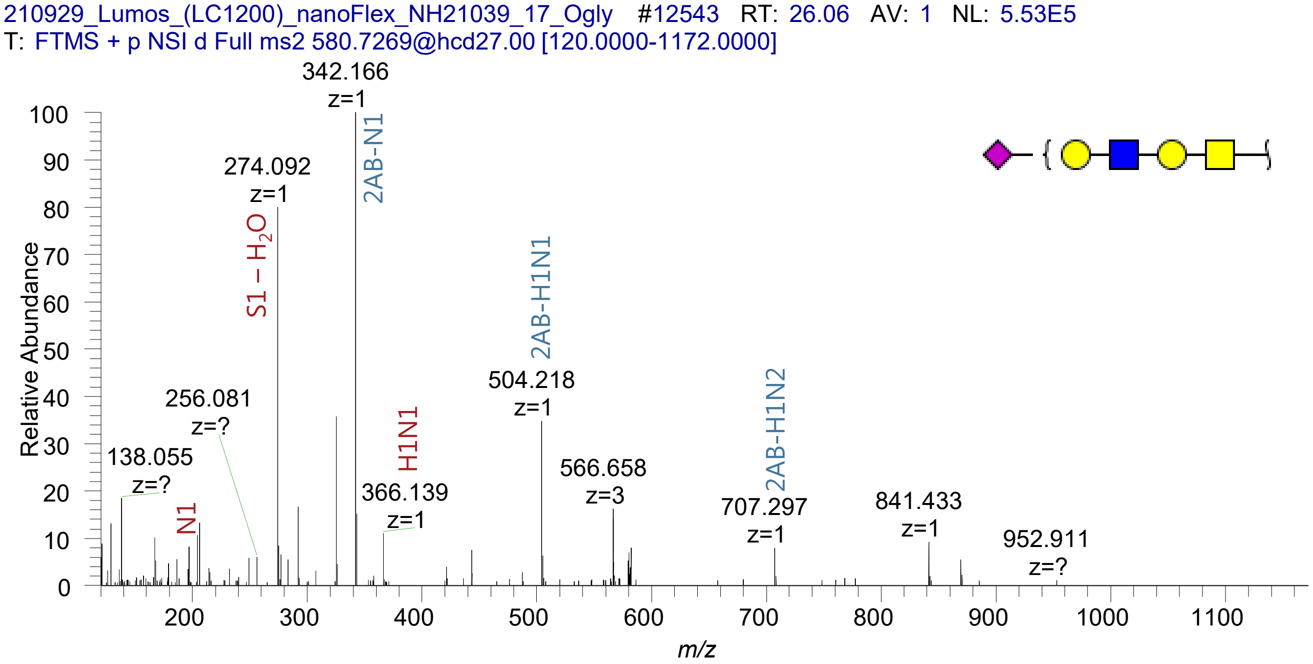

AF H2N2S1 (c)

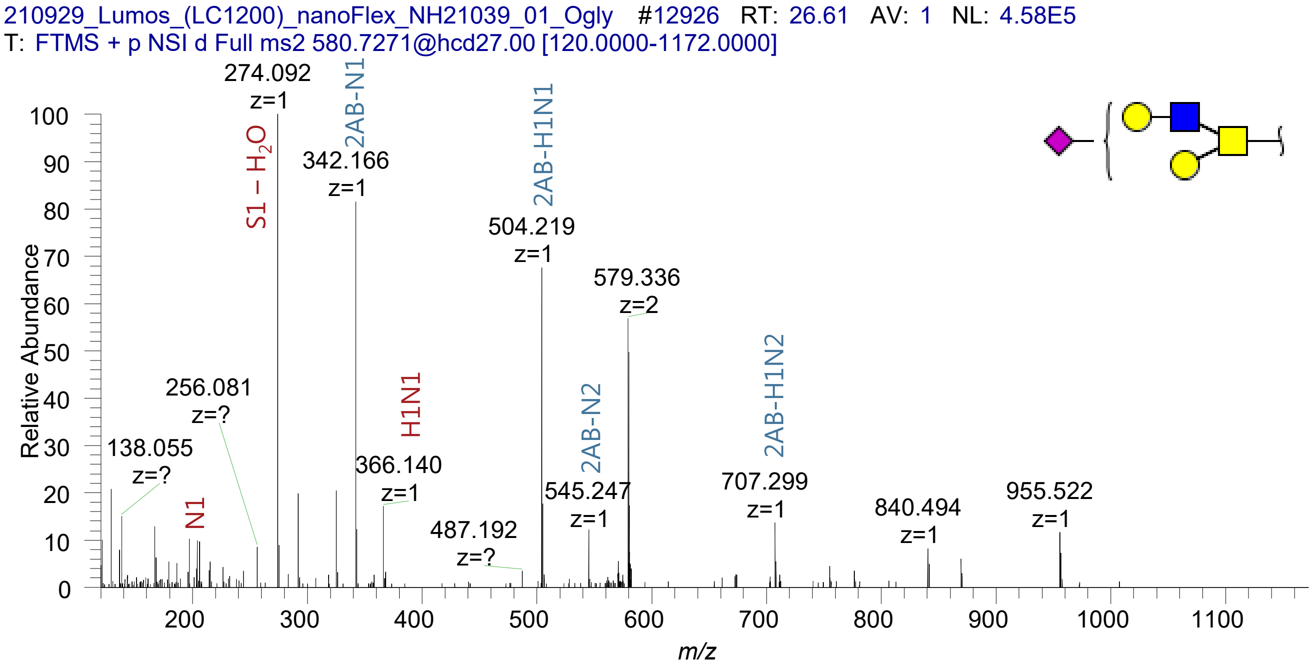

Supporting Figure S2. (Continued)

AG H3N3 (a)

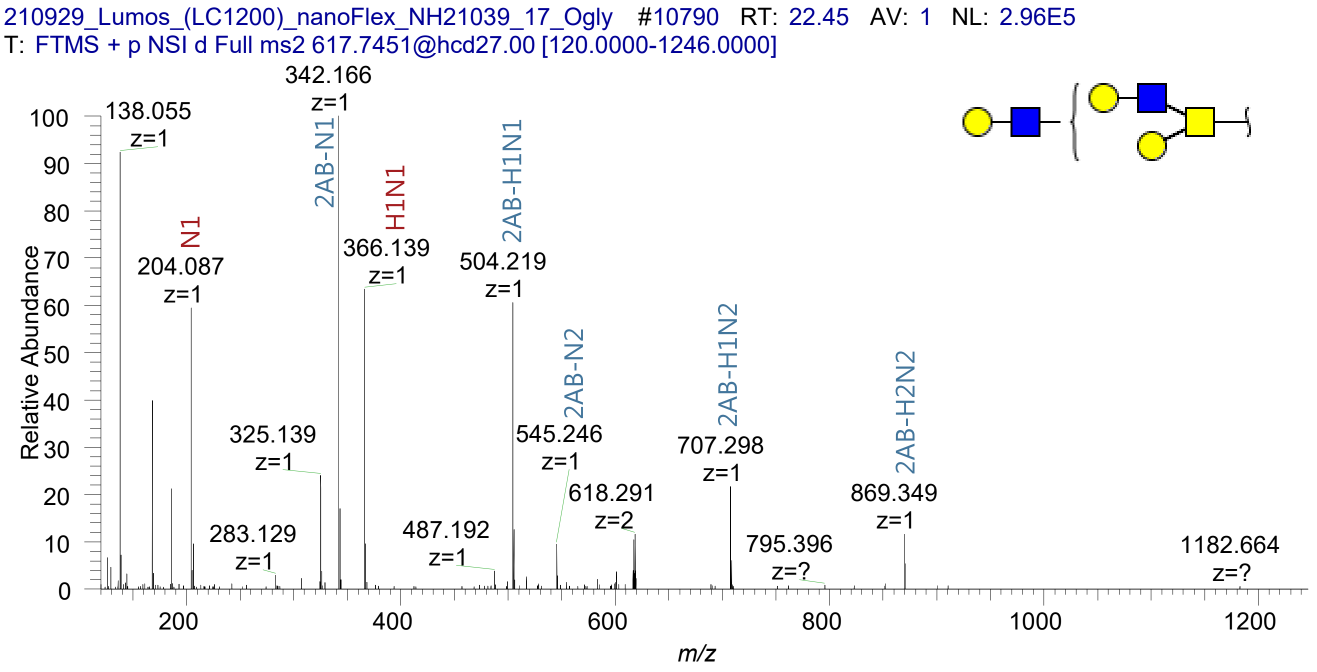

AH H3N3 (b)

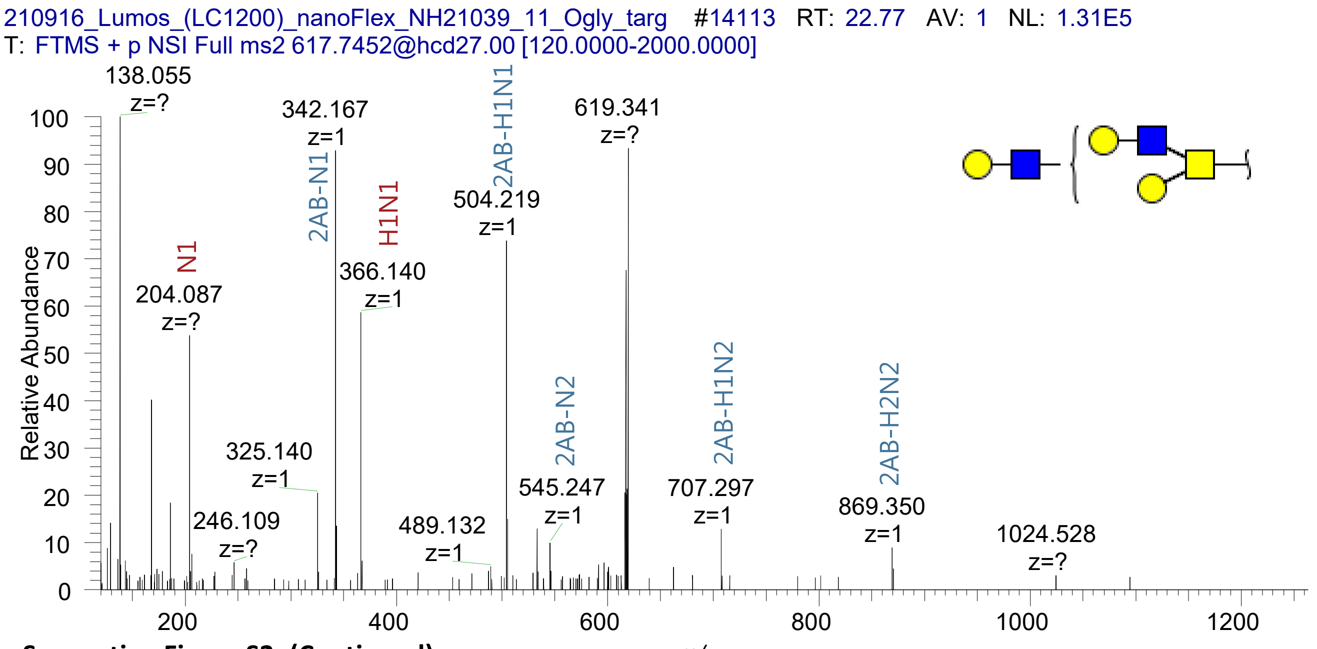

Supporting Figure S2. (Continued)

AI H2N2F1S1 (a)

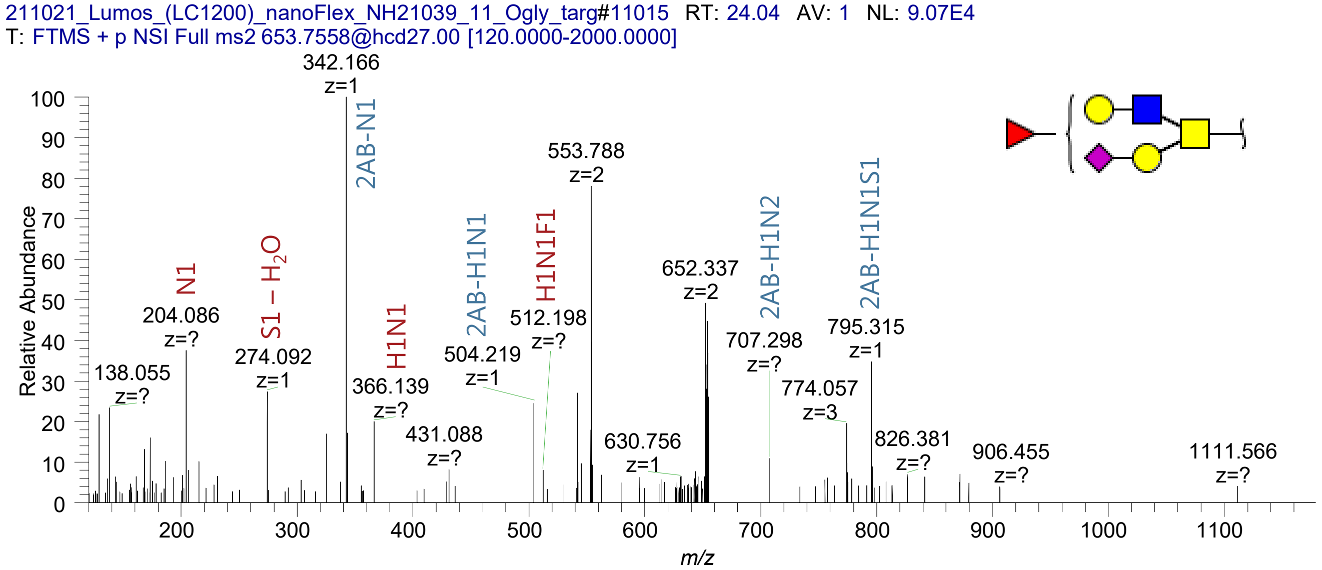

AJ H2N3S1

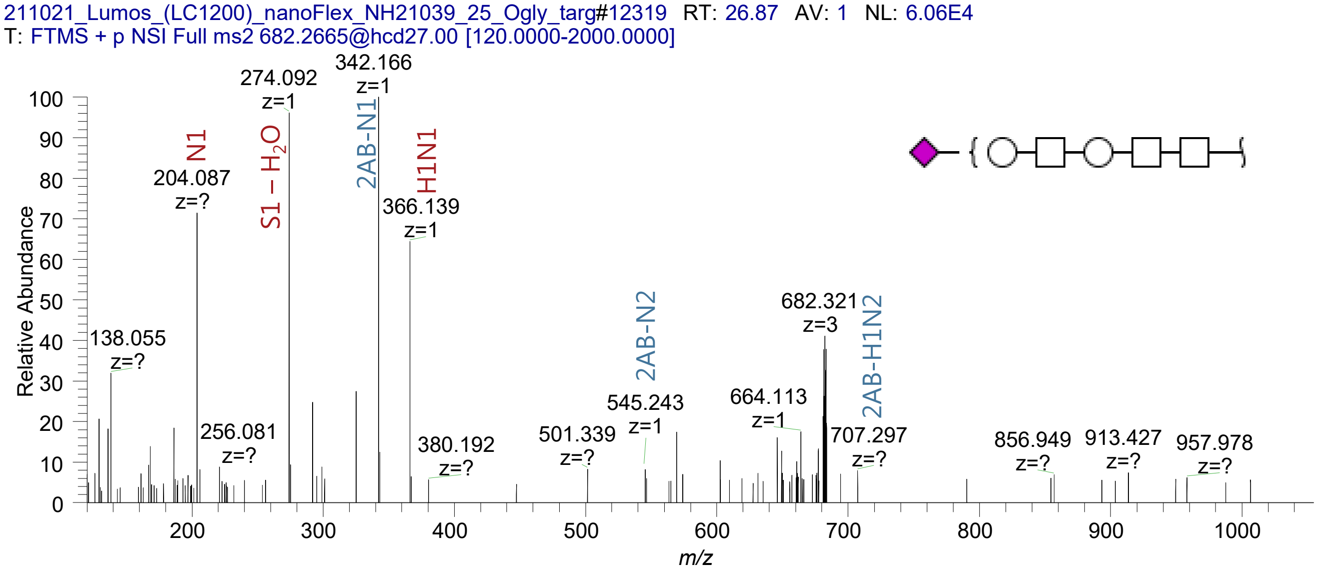

Supporting Figure S2. (Continued)

AK H2N2S2 (a)

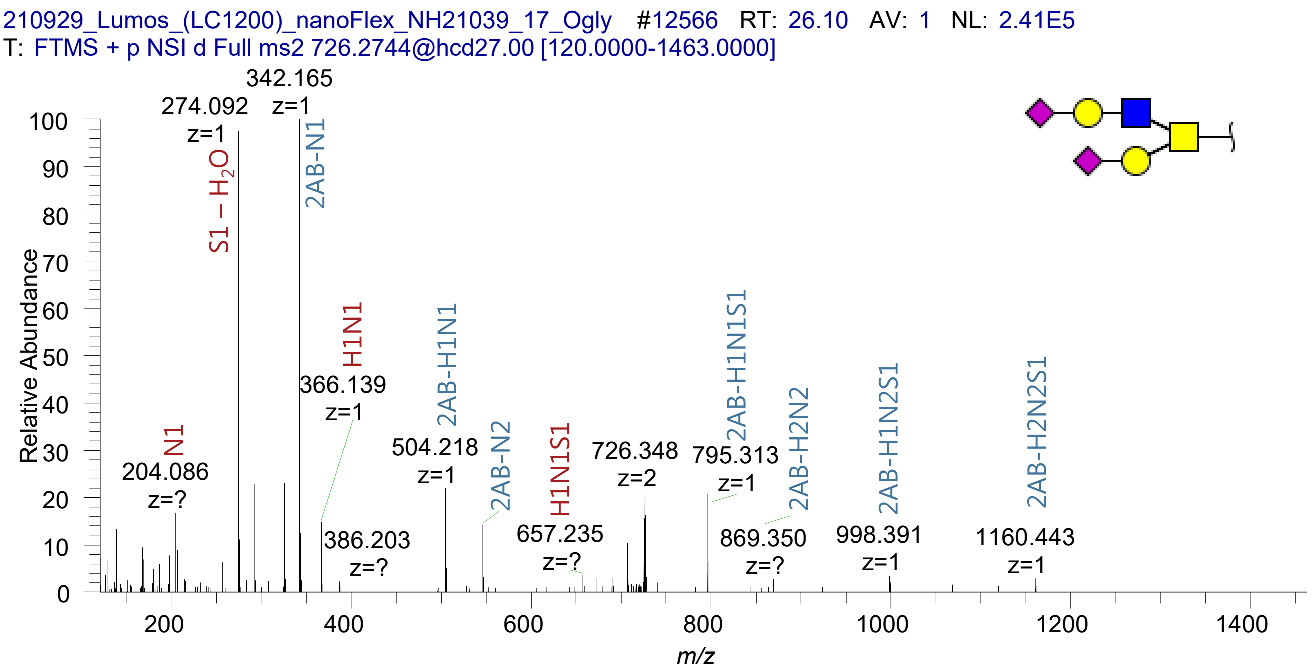

AL H2N2S2 (b)

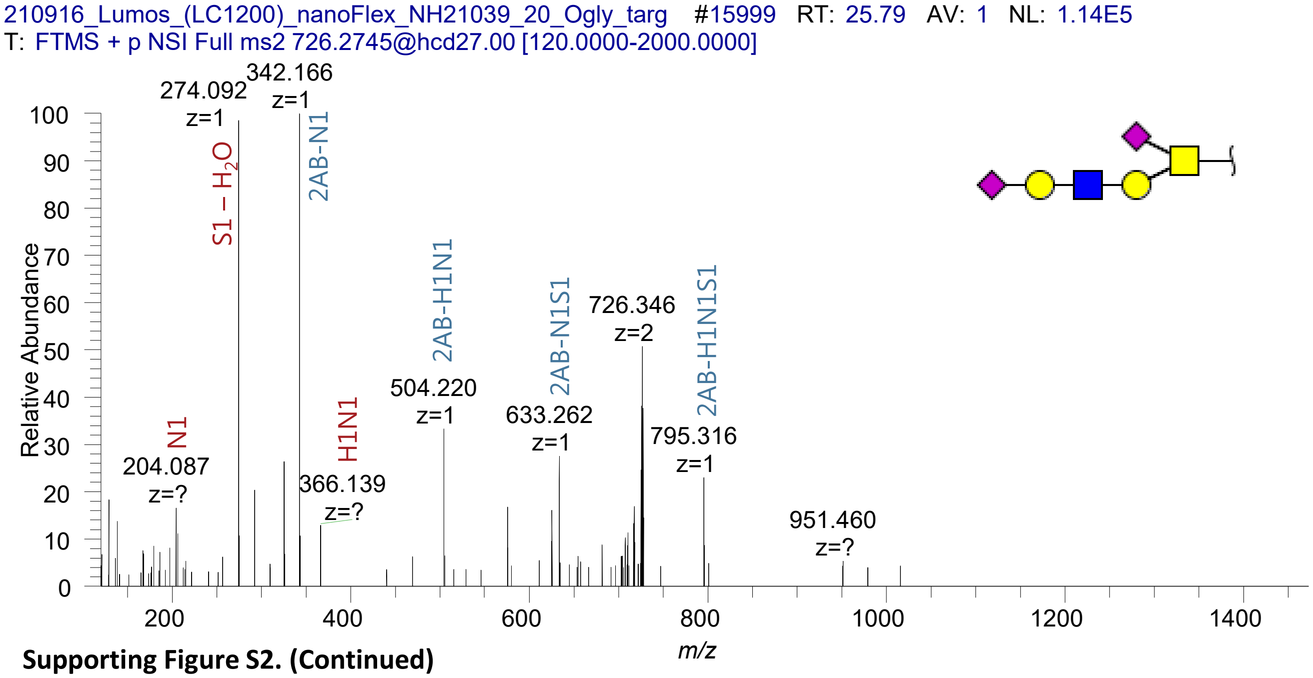

Supporting Figure S2. (Continued)

AM H3N3S1 (a)

210929_Lumos_(LC1200)_nanoFlex_NH21039_17_Ogly #11993 RT: 24.93 AV: 1 NL: 1.73E5
T: FTMS + p NSI d Full ms2 763.7943@hcd27.00 [120.0000-1538.0000]

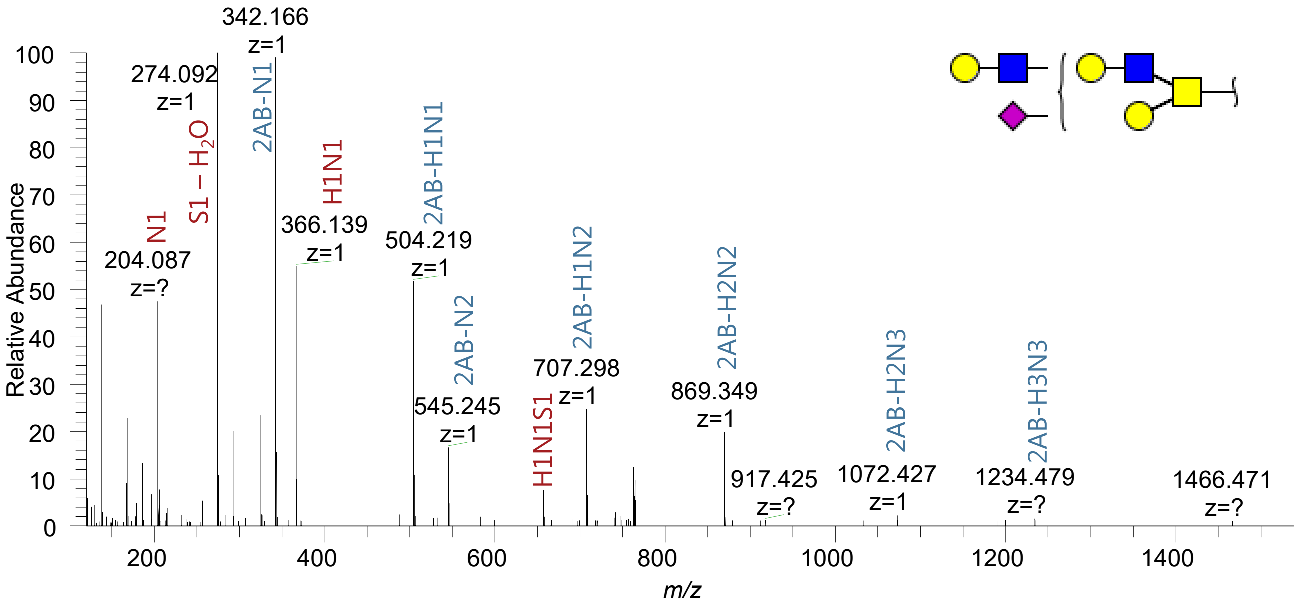

AN H3N3S1 (b)

210929_Lumos_(LC1200)_nanoFlex_NH21039_17_Ogly #12263 RT: 25.48 AV: 1 NL: 3.09E5
T: FTMS + p NSI d Full ms2 763.2927@hcd27.00 [120.0000-1537.0000]

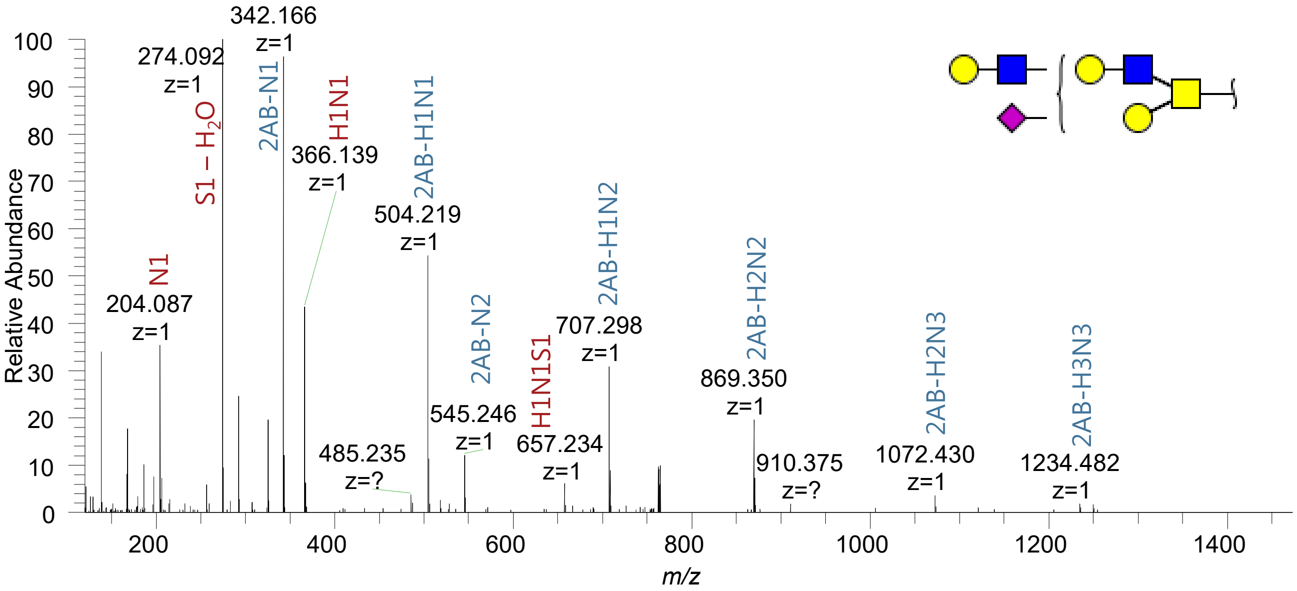

Supporting Figure S2. (Continued)

AO H3N3S2 (a)

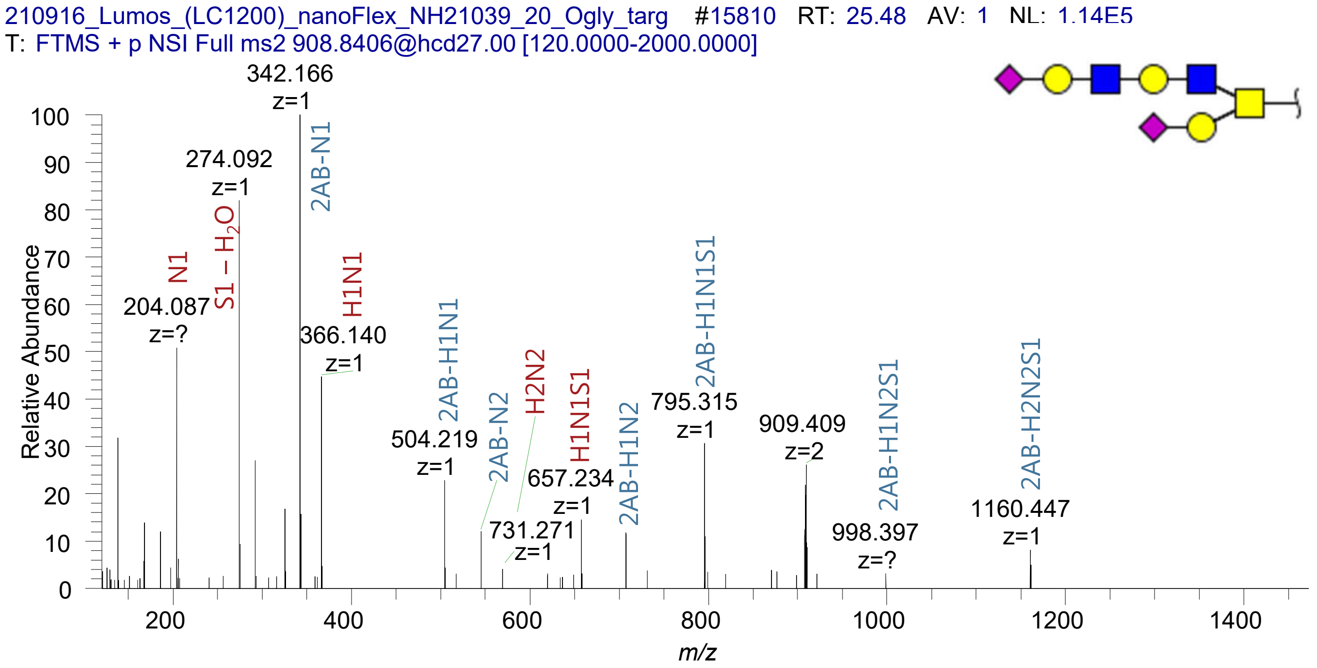

AP H2N3S2 (b)

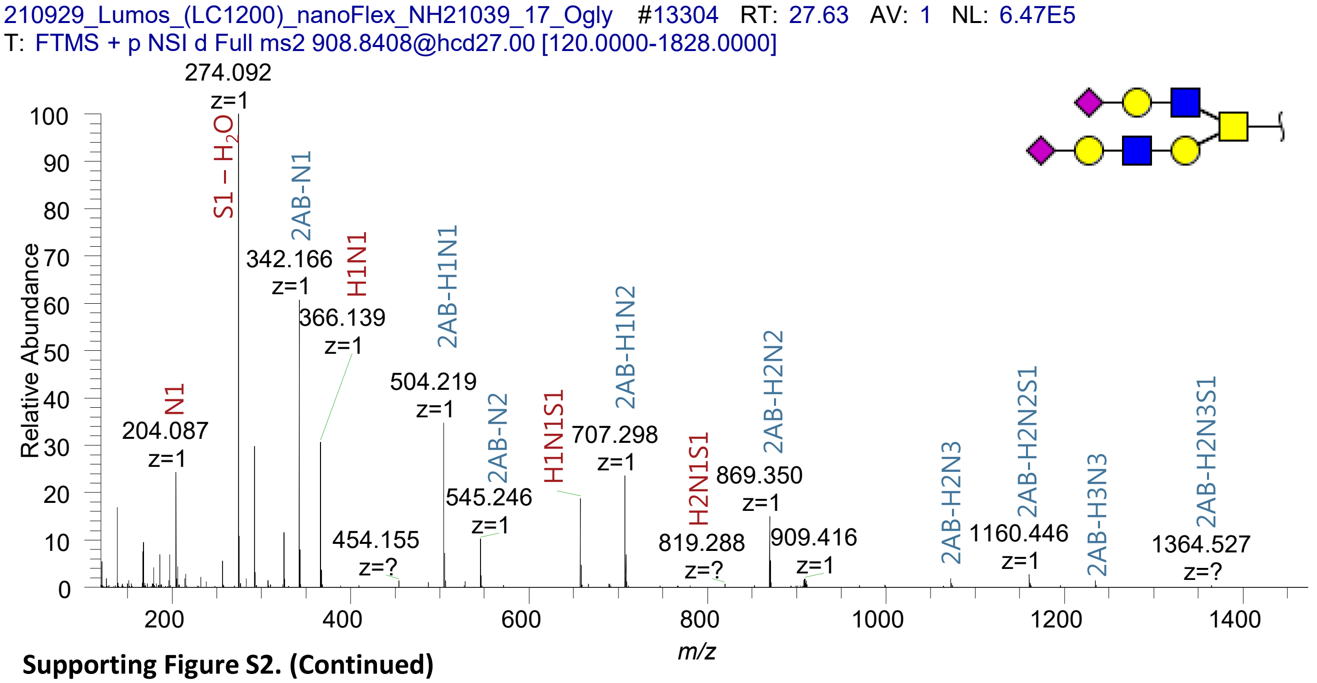

Supporting Figure S2. (Continued)

AQ H4N4S2 (a)

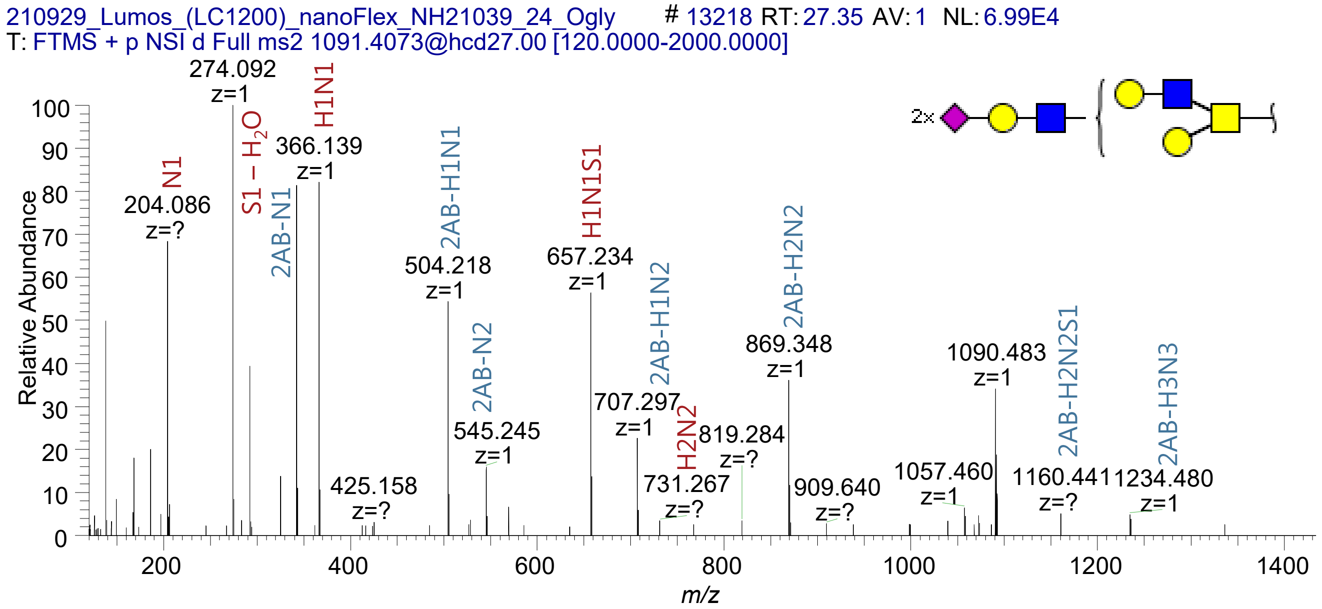

AR H4N4S2 (b)

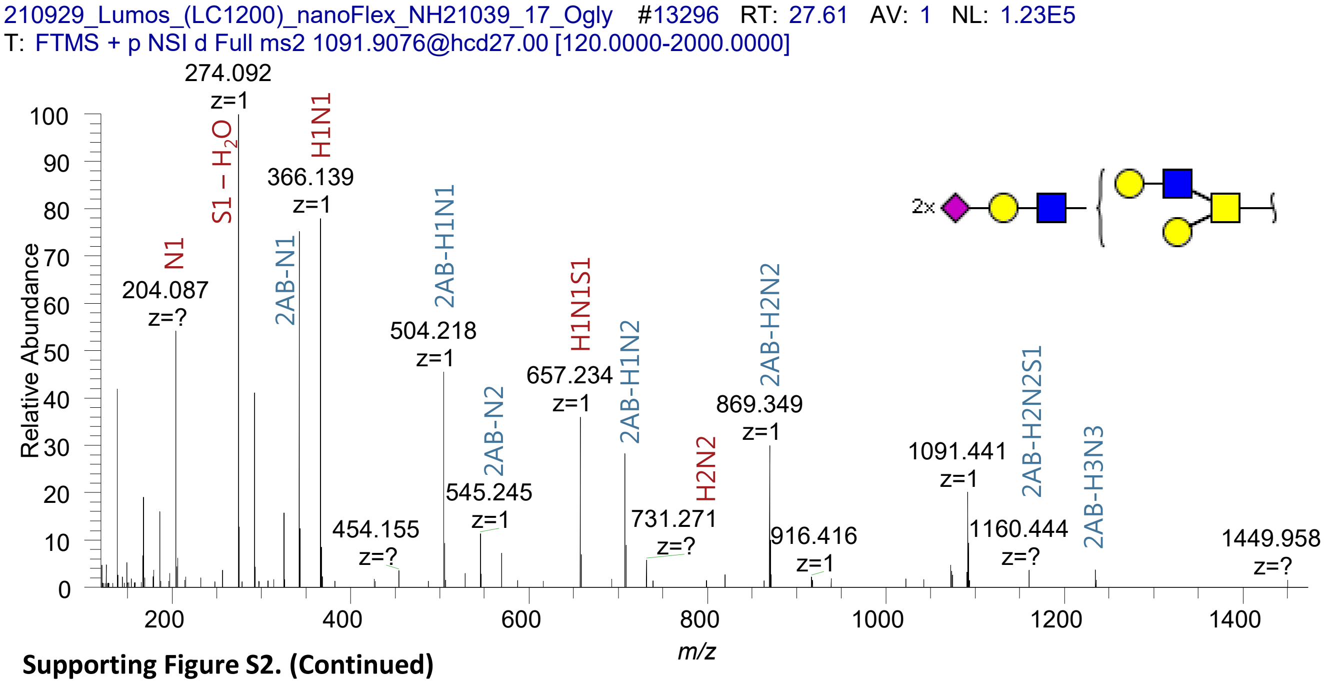

Supporting Figure S2. (Continued)

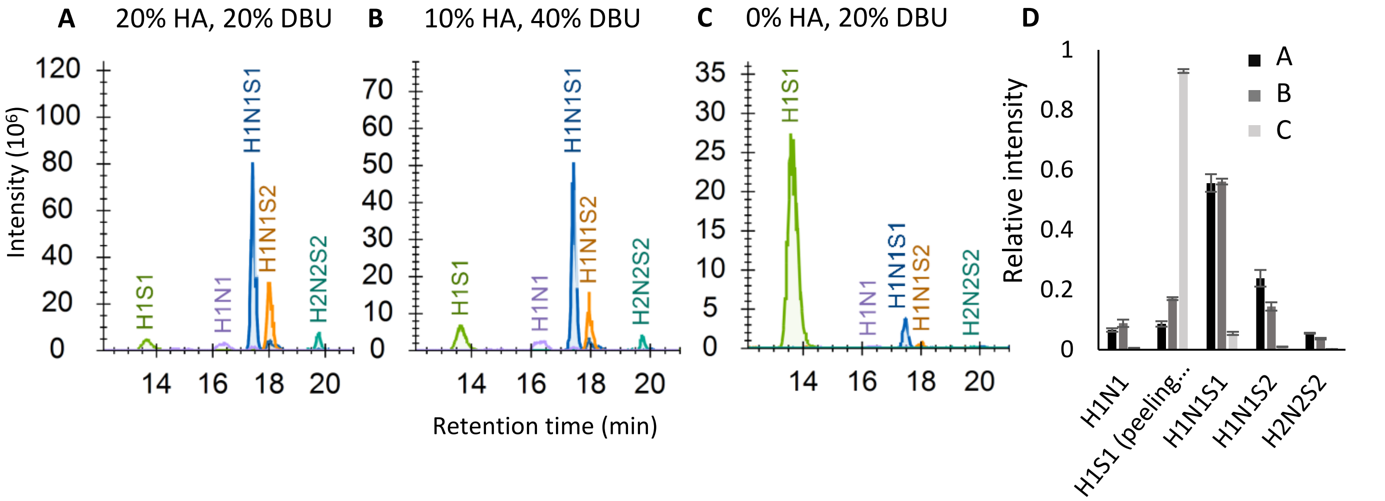

Supporting Figure S3. *O*-Glycans released from fetuin using different concentrations of hydroxylamine. Shown are the extracted ion chromatograms (EICs) of the top 4 highest abundant *O*-glycans on fetuin and the peeling product H1S1. **A**, **B**, and **C**) Representative EICs for the different conditions. **D**) Average relative intensities for the triplicate measurements per condition, with error bars representing the standard deviation. H: Hexose, N: *N*-acetylhexosamine, F: fucose, S: *N*-acetylneuraminic acid.

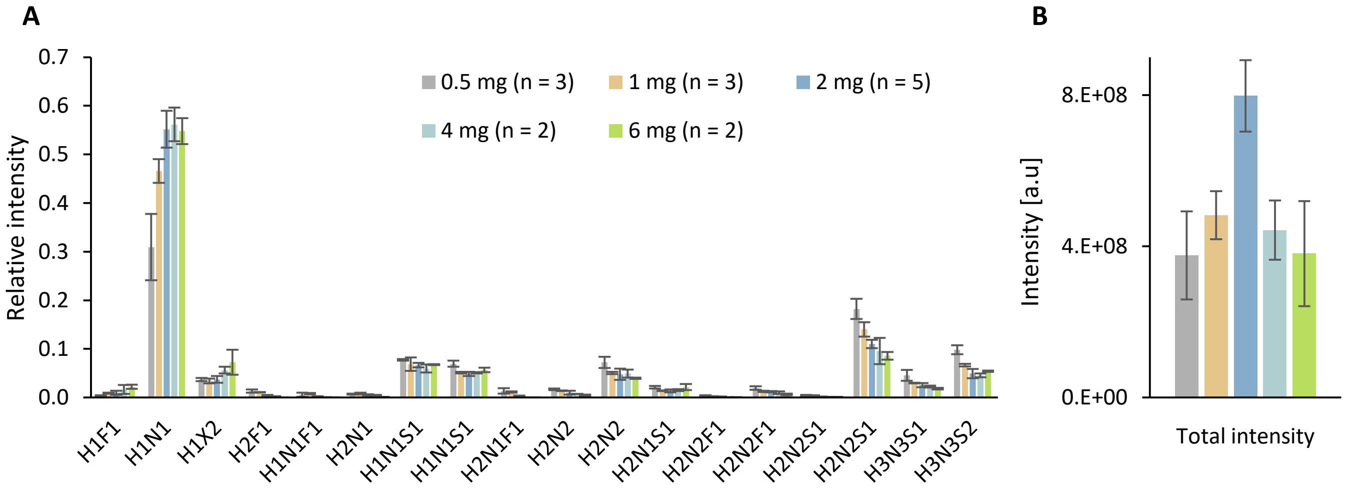

Supporting Figure S4. *O*-Glycans released from approx. 5*10^5 HaCaT^{WT} cells purified using different amounts of hydrazide beads. Between 0.5 to 6 mg hydrazide beads were used, resulting in a maximized recovery using 2 mg. **A**) Average relative intensities of the individual glycans for the technical replicates per condition, with error bars representing the standard deviation. **B**) Average total intensities of the integrated glycan signals for the technical replicates per condition, with error bars representing the standard deviation. Note: HexNAc signals were excluded from analysis, due to sample contamination in these particular experiments. H: Hexose, N: *N*-acetylhexosamine, F: fucose, S: *N*-acetylneuraminic acid.

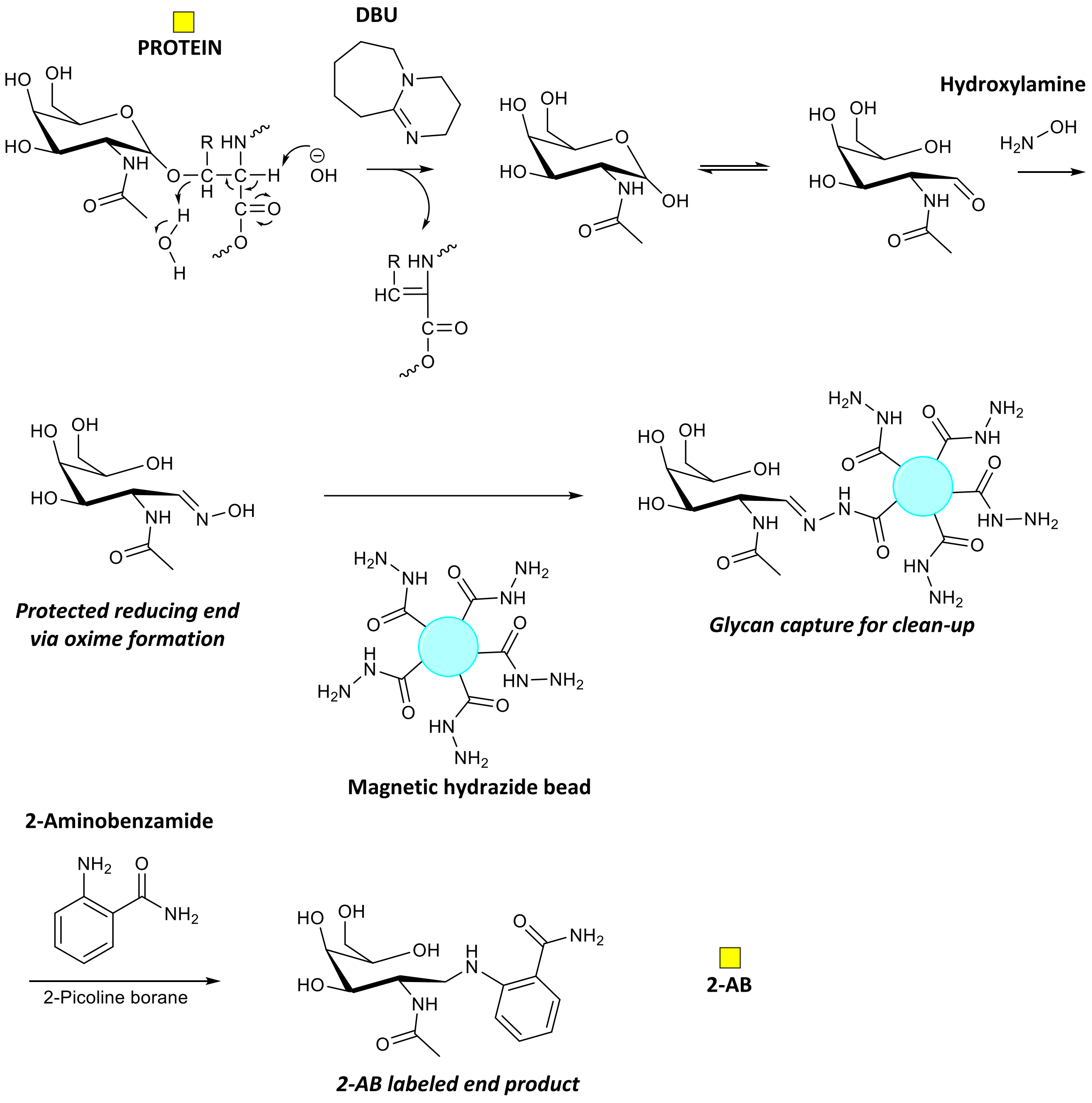

Supporting Figure S5. Scheme representing the *O*-glycan release, clean-up and labeling. Glycan release occurs under basic conditions *via* β-elimination. Due to the presence of hydroxylamine during the release, the released glycans are largely protected from peeling by oxime formation. The released and protected glycans are reversibly bound to hydrazide beads *via* hydrozone formation. After the release-chemicals are washed away, glycans are eluted from the beads and labeled with 2-aminobenzamide *via* reductive amination. *N*-Acetylgalactosamine (GalNAc, yellow square) is displayed as example glycan.

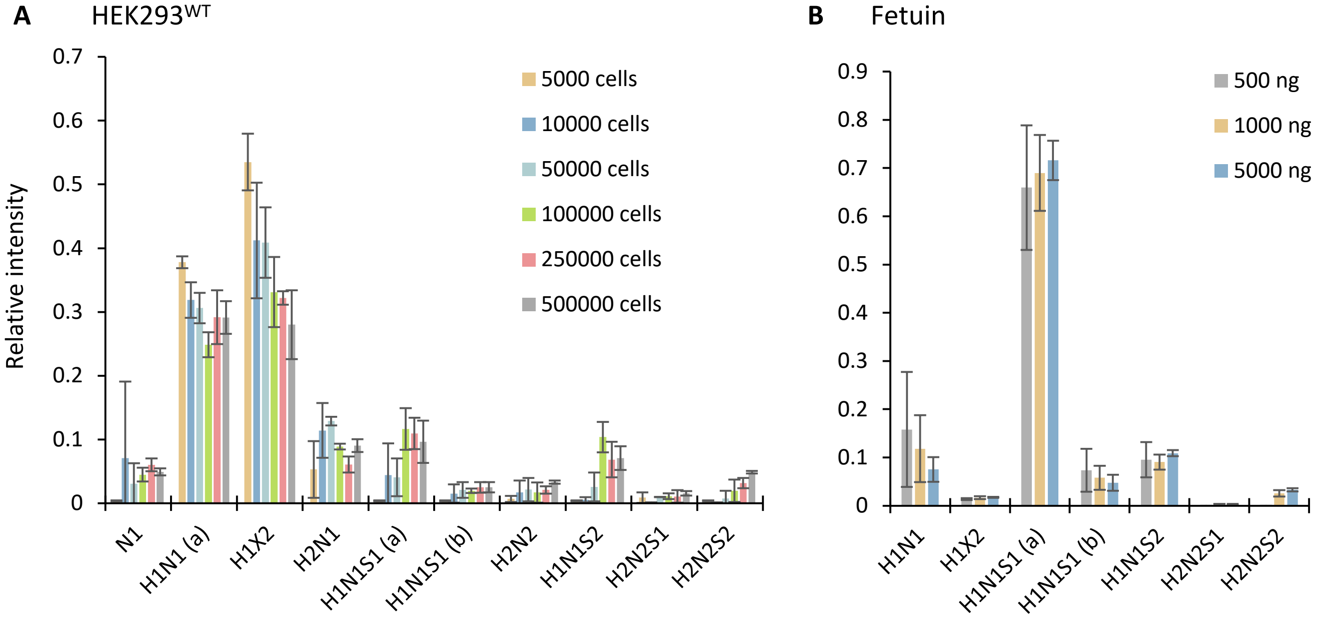

Supporting Figure S6. *O*-glycans released from different amounts of input material. A) Relative abundance of the *O*-glycans released form $5*10^3$ to $5*10^5$ HEK293^{WT} cells (n = 3 for each condition). While the highest abundant glycans were still detected using 5000 cells, non-skewed, repeatable data were obtained using $1*10^5$ cells or more. **B)** Relative abundance of the *O*-glycans released form 500 ng to 5 µg of bovine fetuin (n = 3 for each condition). While the highest abundant glycans were still detected using 500 ng input material, non-skewed, repeatable data were obtained using minimum 1 µg of protein. As fetuin (~50 kDa) contains 4 highly occupied glycosylation sites and the lowest abundant glycan is 0.1% of the total, this corresponds to approx. 0.1 pmol of an individual glycan as starting material to obtain a quantifiable signal.

All samples were prepared and dissolved in 20 µL water as described in the method section. For the samples derived from $5*10^3$, $1*10^4$ and $5*10^4$ cells, 10 µL was injected for LC-MS/MS analysis, for the $1*10^5$ cells, 5 µL was injected and for the samples containing 2.5 or $5*10^5$ cells, 2 µL was injected. For the fetuin samples 2, 2, and 5 µL was injected for the samples derived from 5000, 1000 and 500 ng, respectively. A 30 min LC method was used with a gradient from 3% to 45% of solvent B in 15 min, from 45% to 100% B in the next 5 min and 100% B for the last 10 min at 200 nL/min (solvent A: 0.1% formic acid in water; solvent B: 0.1% formic acid in 80% ACN).

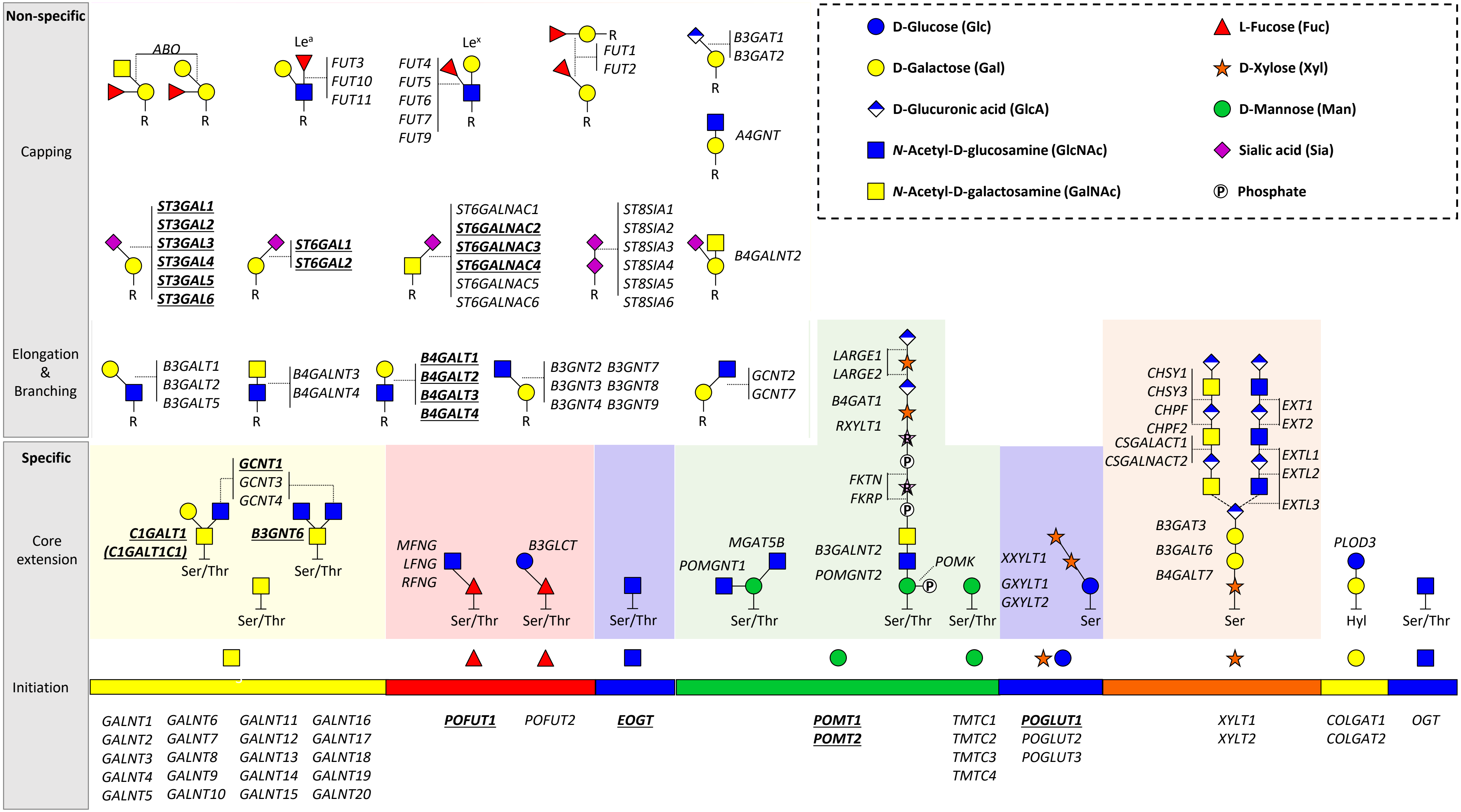

Supporting Figure S7. Schematic overview of *O*-glycan biosynthesis. Shown are the major structural elements of *O*-glycans and the assigned biosynthetic roles of the glycosyltransferases (indicated by the genes encoding these enzymes). The known *O*-glycosylation pathways are organized into the major biosynthetic steps specific for the pathways (initiation and core extension) and those that are non-specific (elongation and branching, and capping). Highlighted in bold are the enzymes targeted in the glycoengineered cell material used in this study.

This overview is based on Schjoldager, K.T., et al., Global view of human protein glycosylation pathways and functions. Nat Rev Mol Cell Biol, 2020. 21(12): p. 729-749.

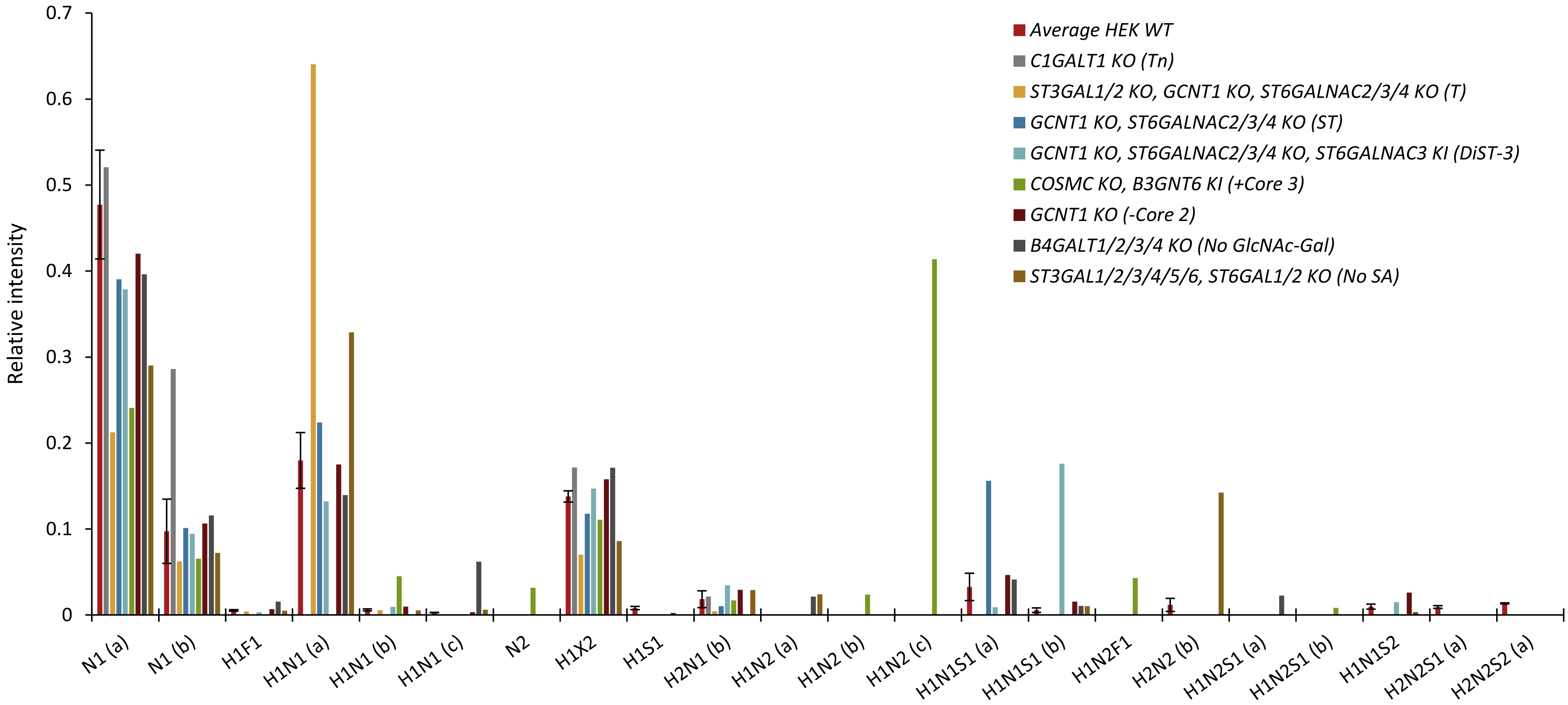

Supporting Figure S8. Relative intensities of HEK293 *O*-glycans. For the WT, average relative intensities for the triplicate measurements per condition are shown, with error bars representing the standard deviation. H: hexose, N: *N*-acetylhexosamine, F: fucose, S: *N*-acetylneuraminic acid. Proposed structures for the glycan compositions can be found in **Supporting Table S5**.

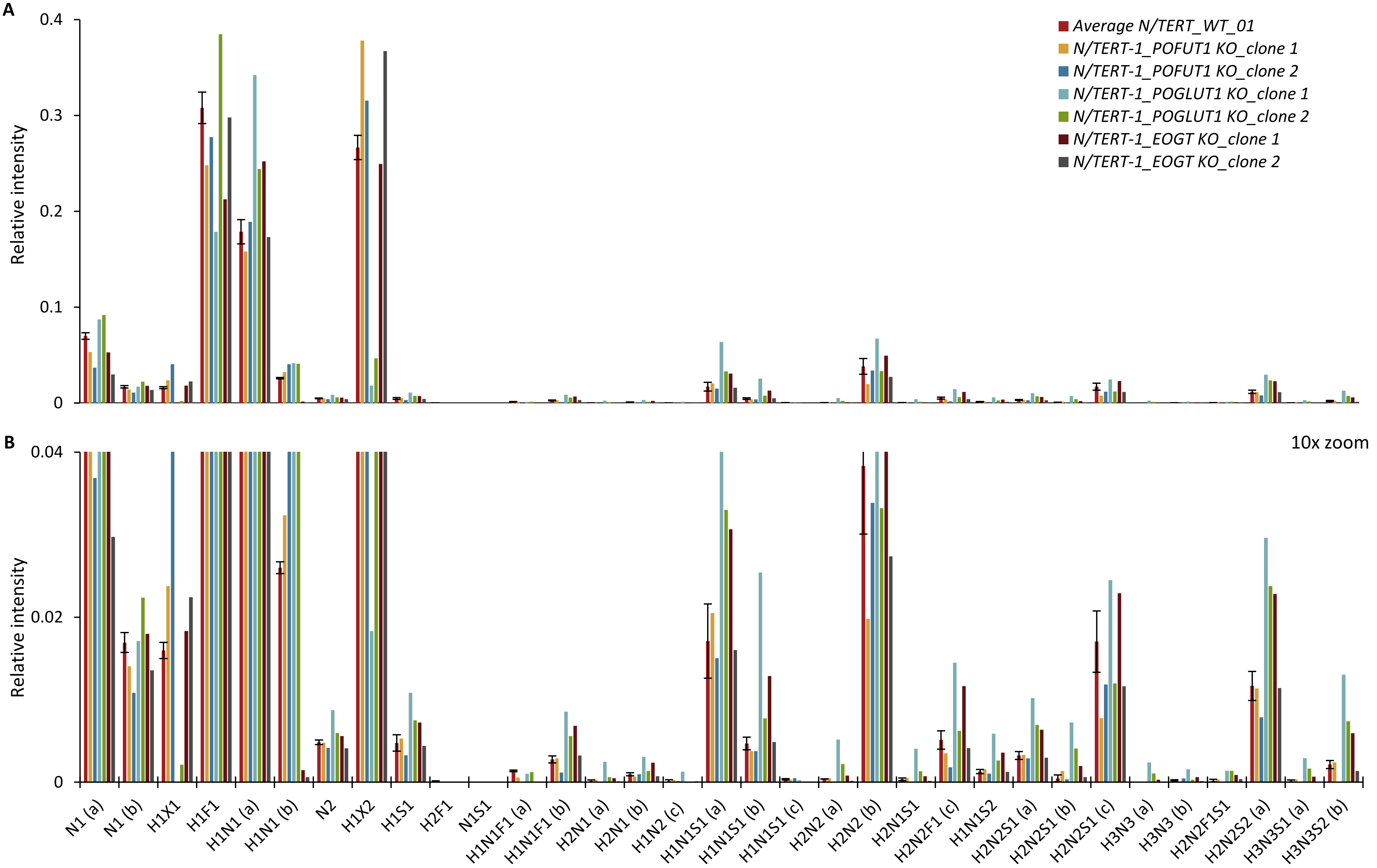

Supporting Figure S9. Relative intensities of N/TERT-1 *O*-glycans. For the WT, average relative intensities for the triplicate measurements per condition are shown, with error bars representing the standard deviation. H: hexose, N: *N*-acetylhexosamine, F: fucose, S: *N*-acetylneuraminic acid. Proposed structures for the glycan compositions can be found in **Supporting Table S6**.

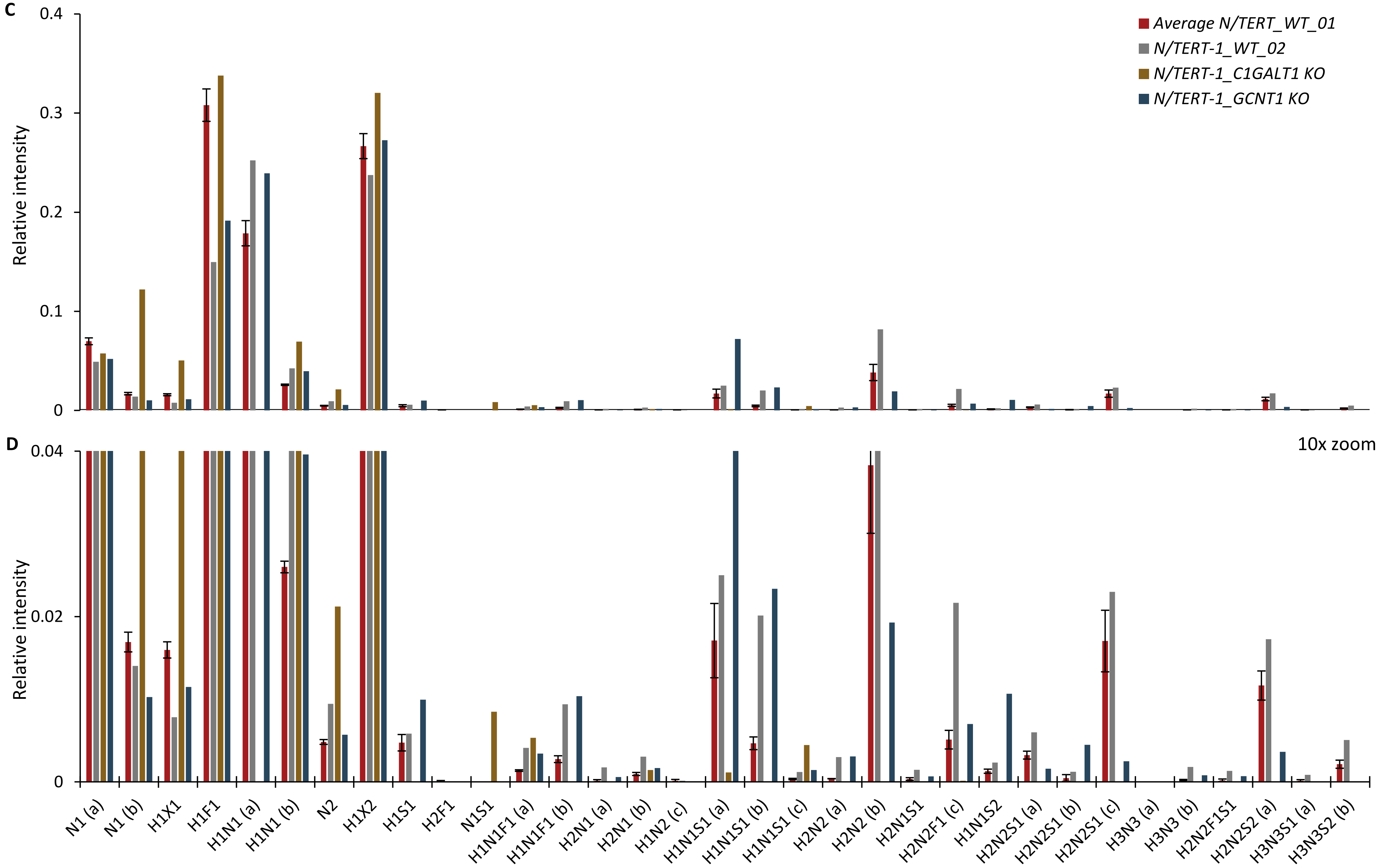

Supporting Figure S9. Relative intensities of N/TERT-1 *O*-glycans. (Continued)

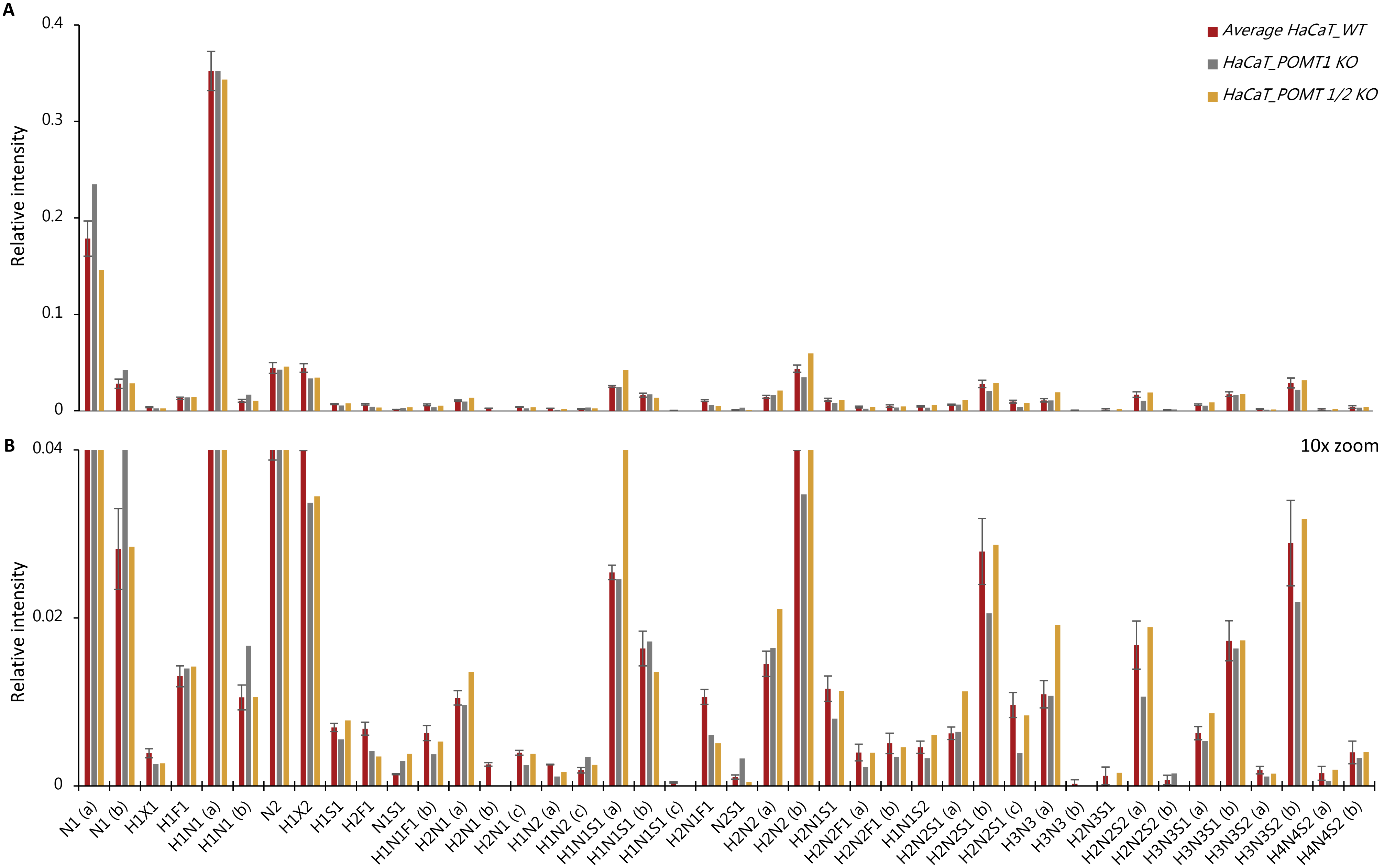

Supporting Figure S10. Relative intensities of HaCaT *O*-glycans. For the WT, average relative intensities for the triplicate measurements per condition are shown, with error bars representing the standard deviation. H: hexose, N: *N*-acetylhexosamine, F: fucose, S: *N*-acetylneuraminic acid. Proposed structures of the glycan compositions can be found in **Supporting Table S6**.

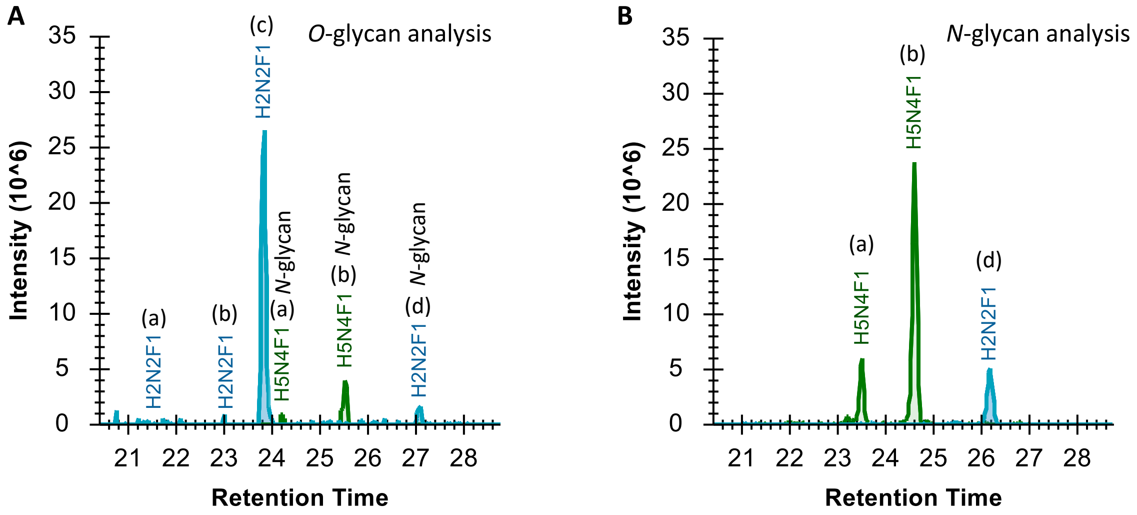

Supporting Figure S11. Examples of isomeric *N*- and *O*-glycan compositions detected in N/TERT-1^{WT}. Shown are the extracted ion chromatograms for H2N2F1 (blue) and H5N4F1 (green) in the (**A**) *O*-glycan and the (**B**) *N*-glycan analysis of the same sample. As *N*-glycans are released first, the *N*-glycan analysis is free of *O*-glycans. However, the *O*-glycan analysis may contain traces of *N*-glycans. Co-analyzing 2-AB labeled *N*- and *O*-glycans from the same samples helps to identify potential *N*-glycan contaminations in the *O*-glycan analysis. Here, two isomers (a and b) with the composition H5N4F1 were detected in the *O*-glycan analysis (**A**). As both (matched on retention time and fragmentation pattern) were also found in the *N*-glycan analysis (**B**), these compositions were assigned to be *N*-glycans (carrying their fucose either on the core (b) or on the antennae (a)). Furthermore, four isomers (a, b, c and d) with the composition H2N2F1 were detected in the *O*-glycan analysis (**A**), of which only (d) was found in the *N*-glycan analysis (**B**). H2N2F1 (a), (b) and (c) were assigned as *O*-GalNAc glycans, which also matched their MS/MS analysis (**Supporting Figure S2**). H: Hexose, N: *N*-acetylhexosamine, F: fucose, S: *N*-acetylneuraminic acid.
